# Supplementary material for: Number transcoding in bilinguals—A transversal developmental study
Source: PLoS One. 2022 Aug 29;17(8):e0273391. doi: 10.1371/journal.pone.0273391 (PMC9423630; doi:10.1371/journal.pone.0273391)
Supplement: S1 File — (DOCX) [file pone.0273391.s001.docx]

# Reading aloud task

## Reaction Times (in ms)

| S1 Table 1: Reading aloud task, reaction times (ms) | | | | | |
| --- | --- | --- | --- | --- | --- |
| Language | Decades | Age | | | |
|  |  | 5^th^ grade | 8^th^ grade | 11^th^ grade | Adults |
| French | ‘30s, ‘40s, ‘50s | 1233.39(55) | 833.16(18) | 817.43(19) | 855.61(35) |
|  | ‘70s, ‘80s, ‘90s | 1516.37(81) | 1053.10(30) | 1006.2(23) | 1092.40(34) |
| German | ‘30s, ‘40s, ‘50s | 722.48(18) | 655.56(13) | 655.83(13) | 697.26(20) |
|  | ‘70s, ‘80s, ‘90s | 732.18(17) | 659.61(11) | 665.52(14) | 688.69(15) |
| Total mean | | 978.67 (25) | 788.65 (11) | 777.93 (10) | 822.59 (15) |
| Note: standard errors in parenthesis | | | | | |

## Correct Responses (in %)

| S1 Table 2: Reading aloud task, correct responses (%) | | | | | |
| --- | --- | --- | --- | --- | --- |
| Language | Decades | Age | | | |
|  |  | 5^th^ grade | 8^th^ grade | 11^th^ grade | Adults |
| French | ‘30s, ‘40s, ‘50s | 91(3) | 99(1) | 98(1) | 97(2) |
|  | ‘70s, ‘80s, ‘90s | 78(4) | 95(2) | 98(1) | 95(2) |
| German | ‘30s, ‘40s, ‘50s | 99(1) | 99(1) | 99(1) | 99(1) |
|  | ‘70s, ‘80s, ‘90s | 98(1) | 100(0) | 99(1) | 99(1) |
| Total mean | | 92(1) | 98(1) | 98(1) | 98(1) |
| Note: standard errors in parenthesis | | | | | |

# Verbal-Visual matching task

## Reaction Times (in ms)

| S1 Table 3: Verbal-visual matching task, reaction times (ms) | | | | | |
| --- | --- | --- | --- | --- | --- |
| Language | Decades | Age | | | |
|  |  | 5^th^ grade | 8^th^ grade | 11^th^ grade | Adults |
| French | ‘30s, ‘40s, ‘50s | 1924.54(93) | 1066.44(34) | 953.01(28) | 879.52(32) |
|  | ‘70s, ‘80s, ‘90s | 2606.68(135) | 1406.40(66) | 1155.80(40) | 1098.50(51) |
| German | ‘30s, ‘40s, ‘50s | 1554.04(51) | 974.10(33) | 824.88(23) | 795.32(30) |
|  | ‘70s, ‘80s, ‘90s | 1600.93(56) | 971.09(37) | 850.20(22) | 826.84(31) |
| Total mean | | 1830.83 (42) | 1095.63(23) | 938.07 (15) | 891.15 (19) |
| Note: standard errors in parenthesis | | | | | |

## Correct Responses (in %)

| S1 Table 4: Verbal-visual matching task, correct responses (%) | | | | | |
| --- | --- | --- | --- | --- | --- |
| Language | Decades | Age | | | |
|  |  | 5^th^ grade | 8^th^ grade | 11^th^ grade | Adults |
| French | ‘30s, ‘40s, ‘50s | 89(2) | 98(1) | 95(2) | 94(2) |
|  | ‘70s, ‘80s, ‘90s | 69(4) | 90(2) | 89(3) | 93(3) |
| German | ‘30s, ‘40s, ‘50s | 91(2) | 96(2) | 93(2) | 99(1) |
|  | ‘70s, ‘80s, ‘90s | 94(2) | 95(2) | 98(2) | 98(1) |
| Total mean | | 87(1) | 94(1) | 94(1) | 96(1) |
| Note: standard errors in parenthesis | | | | | |
