# Supplementary material for: Number transcoding in bilinguals—A transversal developmental study
Source: PLoS One. 2022 Aug 29;17(8):e0273391. doi: 10.1371/journal.pone.0273391 (PMC9423630; doi:10.1371/journal.pone.0273391)
Supplement: S2 File — (DOCX) [file pone.0273391.s002.docx]

# Reading aloud task

z-score transformed RT

The same linear mixed model as for RTs was applied on *z-score* transformed RTs, applied separately for each age group. This transformation aimed to reduce the variability of RT between the four age groups. Note that the same dataset was used, namely after removing post error slipping.

As expected, the transformation disrupted the main effect of Age as well as the two-way interactions and three-way interactions involving Age. Otherwise, these analyses replicated the main effects of Language, Number Size and importantly the two-way interaction between Language and Number Size remained significant (see S1 Table 1).

| S1 Table 1. Results of the reading aloud task’s z-score transformed RT’s linear model | | | | |
| --- | --- | --- | --- | --- |
|  | num *df* | den *df* | F | Pr(>F) |
| Age | 3 | 95.11 | 0.02 | 0.99 |
| Language | 1 | 78.31 | 174.58 | <0.001 |
| Number Size | 1 | 22.20 | 33.41 | <0.001 |
| Age x Language | 3 | 92.08 | 0.64 | 0.59 |
| Age x Number Size | 3 | 1845.05 | 1.26 | 0.29 |
| Language x Number Size | 1 | 21.91 | 50.01 | <0.001 |
| Age x Language x Number Size | 3 | 1845.23 | 1.57 | 0.19 |
| Note: num = numerator, den = denominator | | | | |

Follow-up analysis of the interaction between Language and Number Size confirmed both hypotheses. In French the vigesimal ’70s, ’80s, and ’90s numbers were named slower ($t(21.6)=9.44, p<.001$) than ’30s, ’40s, and ’50s numbers, in line with the expected effect of *transparency of power*. While the same comparison was not significant in German ($t\left( 21.8 \right)=0.03,n.s.$).

Secondly, comparing ’30s, ’40s, and ’50s numbers in both languages, revealed a cost for naming numbers in French (LM2) compared to German (LM1) ($t\left( 56.0 \right)= 7.42,p<.001$) (see S1 Table 2), as expected according to the hypothesis on the effect of *language of math acquisition*.

| S1 Table 2. Reading aloud task’s average z-scores | | | | | |
| --- | --- | --- | --- | --- | --- |
| Language | Size | Age | | | |
|  |  | 5^th^ grade | 8^th^ grade | 11^th^ grade | Adults |
| French | ’30s, ’40s, ’50s | 0.21 | 0.13 | 0.15 | 0.08 |
| French | ’70s, ’80s, ’90s | 0.62 | 0.89 | 0.91 | 0.82 |
| German | ’30s, ’40s, ’50s | -0.53 | -0.49 | -0.50 | -0.41 |
| German | ’70s, ’80s, ’90s | -0.52 | -0.48 | -0.46 | -0.44 |

## Subset data: four-syllable length

A second control for the length of the number words consisted in removing all number words that were longer or shorter than four syllables. This led to a reduction of the dataset from 2080 (after the exclusion of incorrect responses and post-error trials) to 1661 measurement points. The same pattern of results as with the complete dataset was found, besides the three-way interaction between Age Language and Number Size that is here not significant anymore, see S1 Table 3.

| S1 Table 3. Results of the reading aloud task’s linear model on the subset data | | | | |
| --- | --- | --- | --- | --- |
|  | num *df* | den *df* | F | Pr(>F) |
| Age | 3 | 93.12 | 14.94 | <0.001 |
| Language | 1 | 65.72 | 102.24 | <0.001 |
| Number Size | 1 | 16.61 | 22.09 | <0.001 |
| Age x Language | 3 | 90.68 | 13.99 | <0.001 |
| Age x Number Size | 3 | 1433.33 | 0.70 | 0.55 |
| Language x Number Size | 1 | 13.10 | 23.36 | < 0.001 |
| Age x Language x Number Size | 3 | 1434.28 | 0.92 | 0.43 |
| Note: num = numerator, den = denominator | | | | |

Follow-up for the two-way interaction between Age and Language indicated a significant improvement in French between 5^th^ and 8^th^ graders ($t(93.7)=5.79, p<.001$, but no significant changes in German between the four age groups$(\mathrm{all} t(94.0)<2.12, p<.22)$.

Follow-up analyses on the two-way interaction between Language and Number Size confirmed the cost, in French$\left( t\left( 11.7 \right)=5.69, p<.001 \right)$ for the vigesimal ’70s, ’80s, and ’90s numbers. The same comparison was not significant in German$\left( t\left( 20.7 \right)=0.10, p=n.s. \right)$.

Secondly, comparing ’30s, ’40s, and ’50s numbers in both languages, again confirmed the cost for naming numbers in French (LM2) compared to German (LM1) $\left( t(45.6)=6.34, p<.001 \right)$.

# Verbal-Visual Matching task

## RT z-score

The same linear mixed model as for RTs was again applied on *z-score* transformed RTs, applied separately for each age group. Confirming the raw data analyses, the main effects of Language, Number Size, and their interactions remained significant, while disrupting the main effect of Age and the interactions with this factor (see S1 Table 4).

| S1 Table 4. Results of the matching task’s z-score transformed RT’s linear model | | | | |
| --- | --- | --- | --- | --- |
|  | num *df* | den *df* | F | Pr(>F) |
| Age | 3 | 93.94 | 0.11 | 0.95 |
| Language | 1 | 55.14 | 121.07 | <.001 |
| Number Size | 1 | 21.55 | 17.82 | <.001 |
| Age x Language | 3 | 84.89 | 3.31 | 0.02 |
| Age x Number Size | 3 | 1743.89 | 0.95 | 0.42 |
| Language x Number Size | 1 | 21.73 | 65.22 | <.001 |
| Age x Language x Number Size | 3 | 1740.79 | 0.74 | 0.53 |
| Note: num = numerator, den = denominator | | | | |

Follow-up contrast analyses also replicated the results obtained with raw RTs, namely a cost for’70s, ’80s, and ’90s numbers in French ($t(21.4)=6.11,p<.001$), but not for the same numbers in German ($t\left( 21.8 \right)=0.7,p=n.s.$). Secondly, the comparison between ’30s, ’40s, and ’50s in both languages revealed a cost for French (LM2) compared to German (LM1) ($t(44.0)=4.79,p<.001$), see S1 table 5.

| S1 Table 5. Matching task’s average z-scores | | | | | |
| --- | --- | --- | --- | --- | --- |
| Language | Size | Age | | | |
|  |  | 5^th^ grade | 8^th^ grade | 11^th^ grade | Adults |
| French | ’30s, ’40s, ’50 | -0.03 | -0.09 | -0.03 | -0.07 |
| French | ’70s, ’80s, ’90 | 0.65 | 0.50 | 0.50 | 0.48 |
| German | ’30s, ’40s, ’50 | -0.41 | -0.25 | -0.37 | -0.28 |
| German | ’70s, ’80s, ’90 | -0.36 | -0.26 | -0.30 | -0.20 |

## Subset data: four-syllable length

The removal of items corresponding to number words with more or less than 4 syllables reduced the sample from 1998 to 1499 measurement points. Like with the complete dataset, the model replicated all significant main effects and interactions (all F > 10.42, p < .001)(S1 Table 6).

| S1 Table 6. Results of the matching task’s linear model on the subset data | | | | |
| --- | --- | --- | --- | --- |
|  | num *df* | den *df* | F | Pr(>F) |
| Age | 3 | 84.69 | 47.81 | <.001 |
| Language | 1 | 63.74 | 66.35 | <.001 |
| Number Size | 1 | 20.25 | 15.93 | <.001 |
| Age x Language | 3 | 82.41 | 12.31 | <.001 |
| Age x Number Size | 3 | 1248.28 | 10.22 | <.001 |
| Language x Number Size | 1 | 14.93 | 35.79 | <.001 |
| Age x Language x Number Size | 3 | 1249.01 | 10.67 | <.001 |
| Note: num = numerator, den = denominator | | | | |

Follow-up analyses on the three-way interaction between Age, Language, and Number Size revealed slightly different results than those obtained with the complete data set, in particular for the two older age. Indeed, the follow-up contrasts in French resulted in a cost for vigesimal ’70s, ’80s, and ’90s numbers compared to ’30s, ’40s, and ’50s in 5^th^ graders ($t(68.90)=8.54,p<.001$) and 8^th^ graders ($t\left( 39.77 \right)=3.31,p=.01$). This contrast was not significant for 11^th^ graders ($t(44.21)=2.50,p= .09$) and adults ($t\left( 54.29 \right)=2.11,p=.24$). Furthermore, as in previous analyses, the same comparison in German, did not lead to significant differences (all$t< .54, p= n.s.$). Our hypothesis on the cost entailed by transcoding vigesimal numbers (i.e. French ’70s, ’80s, and ’90s) was thus only confirmed for the two youngest age groups with this reduced data set.

Secondly, when comparing ’30s, ’40s, and ’50s numbers in French (LM1) and German (LM1), only 5^th^ graders showed the expected cost in French ($t\left( 127.65 \right)=4.82,p<.001$). This was not the case for 8^th^ and 11^th^ graders and adults $(\mathrm{all} t<1.43,p=n.s.$)*.* Analyses on four-syllables number words did not fully support the hypothesis on the language of math acquisition.

In conclusion, with the reduced four-syllable data set we observed the effect of number word transparency was robustly in 5^th^ and 8^th^ graders, while the LM2 cost was robust only in 5^th^ graders.
