# Supplementary material for: Number transcoding in bilinguals—A transversal developmental study
Source: PLoS One. 2022 Aug 29;17(8):e0273391. doi: 10.1371/journal.pone.0273391 (PMC9423630; doi:10.1371/journal.pone.0273391)
Supplement: S3 File — Number of syllables for each number word in German and in French as used to control for word length. (DOCX) [file pone.0273391.s003.docx]

| **Digit** | **German** | **Syllables** | **PhonoL** | **French** | **Syllables** | **PhonoL** | **Task** |
| --- | --- | --- | --- | --- | --- | --- | --- |
| 34 | vierunddreissig | 4 | 11 | trentequatre | 4 | 8 | Read |
| 35 | fünfunddreissig | 4 | 12 | trentecinq | 3 | 7 | Read |
| 36 | sechsunddreissig | 4 | 12 | trentesix | 3 | 7 | Read |
| 38 | achtunddreissig | 4 | 11 | trentehuit | 3 | 7 | Read |
| 41 | einundvierzig | 4 | 11 | quaranteetun | 4 | 7 | Read |
| 43 | dreiundvierzig | 4 | 12 | quarantetrois | 4 | 9 | Read |
| 45 | fünfundvierzig | 4 | 13 | quarantecinq | 4 | 8 | Read |
| 46 | sechsundvierzig | 4 | 13 | quarantesix | 4 | 8 | Read |
| 51 | einundfünfzig | 4 | 12 | cinquanteetun | 4 | 7 | Read |
| 52 | zweiundfünfzig | 4 | 13 | cinquantedeux | 4 | 8 | Read |
| 57 | siebenundfünfzig | 5 | 15 | cinquantesept | 4 | 8 | Read |
| 59 | neunundfünfzig | 4 | 13 | cinquanteneuf | 4 | 8 | Read |
| 61 | einundsechzig | 4 | 11 | soixanteetun | 4 | 8 | Read |
| 63 | dreiundsechzig | 4 | 12 | soixantetrois | 4 | 10 | Read |
| 65 | fünfundsechzig | 4 | 13 | soixantecinq | 4 | 9 | Read |
| 69 | neunundsechzig | 4 | 12 | soixanteneuf | 4 | 9 | Read |
| 72 | zweiundsiebzig | 4 | 12 | soixantedouze | 5 | 9 | Read |
| 74 | vierundsiebzig | 4 | 12 | soixantequatorze | 5 | 13 | Read |
| 78 | achtundsiebizig | 4 | 12 | soixantedixhuit | 5 | 12 | Read |
| 79 | neunundsiebzig | 4 | 12 | soixantedixneuf | 5 | 12 | Read |
| 81 | einundachtzig | 4 | 10 | quatrevingtun | 4 | 7 | Read |
| 83 | dreiundachtzig | 4 | 11 | quatrevingttrois | 4 | 10 | Read |
| 84 | vierundachtzig | 4 | 11 | quatrevingtquatre | 5 | 10 | Read |
| 86 | sechsundachtzig | 4 | 12 | quatrevingtsix | 5 | 9 | Read |
| 93 | dreiundneunzig | 4 | 12 | quatrevingttreize | 4 | 10 | Read |
| 95 | fünfundneunzig | 4 | 13 | quatrevingtquinze | 4 | 9 | Read |
| 96 | sechsundneunzig | 4 | 13 | quatrevingtseize | 4 | 9 | Read |
| 98 | achtundneunzig | 4 | 12 | quatrevingtdixhuit | 5 | 12 | Read |
| 31 | einunddreissig | 4 | 10 | trenteetun | 3 | 6 | Match |
| 32 | zweiunddreissig | 4 | 11 | trentedeux | 3 | 6 | Match |
| 37 | seiebenunddreissig | 5 | 13 | trentesept | 3 | 7 | Match |
| 39 | neununddreissig | 4 | 11 | trenteneuf | 3 | 7 | Match |
| 42 | zweiundvierzig | 4 | 12 | quarantedeux | 4 | 7 | Match |
| 47 | siebenundvierzig | 5 | 14 | quarantesept | 4 | 8 | Match |
| 48 | achtundvierzig | 4 | 12 | quarantehuit | 4 | 8 | Match |
| 49 | neunundvierzig | 4 | 12 | quaranteneuf | 4 | 8 | Match |
| 53 | dreiundfünfzig | 4 | 13 | cinquanetrois | 4 | 9 | Match |
| 54 | vierundfünfzig | 4 | 13 | cinquantequatre | 5 | 9 | Match |
| 56 | sechsundfünfzig | 5 | 14 | cinquantesix | 4 | 8 | Match |
| 58 | achtundfünfzig | 4 | 13 | cinquantehuit | 4 | 8 | Match |
| 62 | zweiundsechzig | 4 | 12 | soixantedeux | 4 | 8 | Match |
| 64 | vierundsechzig | 4 | 12 | soixantequatre | 5 | 10 | Match |
| 67 | siebenundsechzig | 5 | 14 | soixantesept | 4 | 9 | Match |
| 68 | achtundsechzig | 4 | 12 | soixantehuit | 4 | 9 | Match |
| 71 | einundsiebzig | 4 | 11 | soixanteetonze | 4 | 8 | Match |
| 73 | dreiundsiebzig | 4 | 12 | soixantetreize | 4 | 10 | Match |
| 75 | fünfundsiebizig | 4 | 13 | soixantequinze | 4 | 9 | Match |
| 76 | sechsundsiebzig | 4 | 13 | soixanteseize | 4 | 9 | Match |
| 82 | zweiundachtzig | 4 | 11 | quatrevingtdeux | 4 | 8 | Match |
| 85 | fünfundachtzig | 4 | 12 | quatrevingtcinq | 4 | 9 | Match |
| 87 | siebenundachtzig | 5 | 13 | quatrevingtsept | 4 | 9 | Match |
| 89 | neunundachtzig | 4 | 11 | quatrevingtneuf | 4 | 9 | Match |
| 91 | einundneunzig | 4 | 11 | quatrevingtonze | 4 | 8 | Match |
| 92 | zweiundneunzig | 4 | 12 | quatrevingtdouze | 4 | 9 | Match |
| 94 | vierundneunzig | 4 | 12 | quatrevingtquatorze | 5 | 13 | Match |
| 97 | siebenundneunzig | 5 | 14 | quatrevingtdixsept | 5 | 12 | Match |

Note: Read = reading aloud task. Match = verbal-visual matching task. Phonological lengths (PhonoL) were retrieved from CLEARPOND [1]

1. Marian V, Bartolotti J, Chabal S, Shook A. CLEARPOND: Cross-Linguistic Easy-Access Resource for Phonological and Orthographic Neighborhood Densities. PLOS ONE. 2012;7. doi:10.1371/journal.pone.0043230
