# Supplementary material for: Number transcoding in bilinguals—A transversal developmental study
Source: PLoS One. 2022 Aug 29;17(8):e0273391. doi: 10.1371/journal.pone.0273391 (PMC9423630; doi:10.1371/journal.pone.0273391)
Supplement: S4 File — Analyses carried on each decades. Linear mixed models for both tasks’ reaction times, but replacing the Number Size factor’s level with decades from ‘30s until ‘90s (instead of small and large). (DOCX) [file pone.0273391.s004.docx]

# Supplementary Analyses per decades:

In the following additional analyses with linear mixed models, we compared all decades. That is, in the following, the factor *Number* *Size*, has **8 levels: ‘30s, ‘40s, ‘50s, ‘60s, ‘70s, ‘80s, ‘90s**. We used exactly the same model for reaction times as described in the results (i.e. (A)) on the data removing the post-error slow down. All degrees of freedom are calculated with Satterthwaite approximation.

## Reading aloud task:

| S4 Table 1: results of the linear mixed model *per decades* | | | | |
| --- | --- | --- | --- | --- |
|  | num Df | den Df | F | Pr (>F) |
| Age | 3 | 93.15 | 17.64 | < 0.001 |
| Language | 1 | 97.84 | 682.33139.44 | <0.001 |
| Number Size | 6 | 20.93 | 6.41 | <0.001 |
| Age x Language | 3 | 90.09 | 17.48 | <0.001 |
| Age x Number Size | 18 | 2155.72 | 2.84 | 0.005 |
| Language x Number Size | 6 | 21.00 | 13.35 | < 0.001 |
| Age x Language x Number Size | 18 | 2155.15 | 2.04 | 0.006 |
| Note: Number Size has 8 levels: ‘30s, ‘40s, ‘50s, ‘60s, ‘70s, ‘80s, ‘90s. | | | | |

###

### “Stepwise” contrasts:

The following custom contrasts compare each decade with the previous and following one. For example ‘30 vs ‘40, ‘40 vs ‘50, etc. Contrasts are calculated on estimated marginal means, degrees of freedom from Satterthwaite approximation and p-values are Bonferroni corrected.

| S4 Table 2: Contrasts between subsequent decades | | | | | | |
| --- | --- | --- | --- | --- | --- | --- |
| French | | | | | | |
| Age | contrast | estimate | SE | df | t.ratio | p.value |
| 5^th^ grade | 30 - 40 | -60.74 | 64.20 | 68.00 | -0.95 | 0.35 |
|  | 40 - 50 | -75.19 | 64.30 | 68.60 | -1.17 | 0.25 |
|  | 50 - 60 | -57.31 | 64.50 | 69.30 | -0.89 | 0.38 |
|  | 60 - 70 | -386.08 | 70.70 | 98.30 | -5.46 | <.0001 |
|  | 70 - 80 | 227.47 | 71.5 1 | 2.60 | 3.18 | 0.001 |
|  | 80 - 90 | -17.00 | 67.10 | 80.70 | -0.25 | 0.80 |
|  | | | | | | |
| 8^th^ grade | 30 - 40 | 70.38 | 58.2 | 46.3 | 1.209 | 0.2328 |
|  | 40 - 50 | -17.61 | 58.1 | 45.9 | -0.303 | 0.7632 |
|  | 50 - 60 | -48.96 | 58.1 | 46 | -0.842 | 0.4042 |
|  | 60 - 70 | -147.22 | 60.1 | 52.5 | -2.448 | 0.0177 |
|  | 70 - 80 | -50.7 | 60.9 | 55.3 | -0.832 | 0.409 |
|  | 80 - 90 | 17.12 | 59.4 | 50 | 0.288 | 0.7743 |
|  | | | | | | |
| 11^th^ grade | 30 - 40 | 42.62 | 59.9 | 51.7 | 0.712 | 0.4796 |
|  | 40 - 50 | -26.24 | 60 | 52.1 | -0.437 | 0.6636 |
|  | 50 - 60 | -100.27 | 59.6 | 50.6 | -1.684 | 0.0984 |
|  | 60 - 70 | -61.74 | 60.6 | 54.3 | -1.018 | 0.313 |
|  | 70 - 80 | -114.42 | 63 | 63.1 | -1.816 | 0.074 |
|  | 80 - 90 | 168.64 | 61.6 | 57.8 | 2.738 | 0.0082 |
|  | | | | | | |
| Adults | 30 - 40 | 83.6 | 64.3 | 68.6 | 1.3 | 0.198 |
|  | 40 - 50 | -58.38 | 64.2 | 68.1 | -0.909 | 0.3664 |
|  | 50 - 60 | 15.68 | 64 | 67.1 | 0.245 | 0.807 |
|  | 60 - 70 | -290.96 | 65.6 | 74.1 | -4.435 | <.0001 |
|  | 70 - 80 | 44.51 | 68.5 | 87.5 | 0.65 | 0.5175 |
|  | 80 - 90 | 19.82 | 66.7 | 78.8 | 0.297 | 0.7671 |
|  | | | | | | |
| German | | | | | | |
| Age | contrast | estimate | SE | df | t.ratio | p.value |
| 5^th^ grade | 30 - 40 | 27.98 | 56.9 | 56 | 0.492 | 0.6248 |
|  | 40 - 50 | 5.04 | 56.9 | 56.1 | 0.089 | 0.9297 |
|  | 50 - 60 | -33.46 | 57.6 | 58.8 | -0.581 | 0.5636 |
|  | 60 - 70 | -21.63 | 57.7 | 59.1 | -0.375 | 0.709 |
|  | 70 - 80 | 54.19 | 56.9 | 56.1 | 0.953 | 0.3449 |
|  | 80 - 90 | -13.34 | 56.7 | 55.5 | -0.235 | 0.815 |
|  | | | | | | |
| 8^th^ grade | 30 - 40 | 15.67 | 55.5 | 50.8 | 0.282 | 0.7788 |
|  | 40 - 50 | 47.99 | 55.3 | 50.1 | 0.868 | 0.3896 |
|  | 50 - 60 | -11.74 | 55.2 | 49.7 | -0.213 | 0.8323 |
|  | 60 - 70 | -33.47 | 55.2 | 49.7 | -0.607 | 0.5468 |
|  | 70 - 80 | 40.89 | 55.7 | 51.7 | 0.734 | 0.4666 |
|  | 80 - 90 | -67.49 | 55.8 | 51.8 | -1.211 | 0.2316 |
|  | | | | | | |
| 11^th^ grade | 30 - 40 | 71.35 | 57.6 | 58.9 | 1.238 | 0.2205 |
|  | 40 - 50 | 38.87 | 58.1 | 61 | 0.669 | 0.506 |
|  | 50 - 60 | -34.06 | 57.4 | 58 | -0.594 | 0.5549 |
|  | 60 - 70 | -21.73 | 57.4 | 58 | -0.379 | 0.7062 |
|  | 70 - 80 | 35.96 | 57.2 | 57.3 | 0.629 | 0.532 |
|  | 80 - 90 | -72.91 | 57.4 | 58.1 | -1.271 | 0.2089 |
|  | | | | | | |
| Adults | 30 - 40 | 19.18 | 61.3 | 75.4 | 0.313 | 0.7554 |
|  | 40 - 50 | 114.77 | 62 | 78.6 | 1.852 | 0.0678 |
|  | 50 - 60 | -10.61 | 62.5 | 81 | -0.17 | 0.8656 |
|  | 60 - 70 | -54.77 | 62.3 | 80.1 | -0.879 | 0.382 |
|  | 70 - 80 | 17.14 | 61.2 | 74.6 | 0.28 | 0.78 |
|  | 80 - 90 | -56.22 | 60.9 | 73.3 | -0.924 | 0.3587 |
|  | | | | | | |

In French, ‘30s, ‘40s, ‘50s numbers have comparable RTs. However, ‘60s have significant faster RT than ‘70s for 5^th^, 8^th^ and adults. 11^th^ graders show a marginally significant (p = .07) slow-down between ‘70s and ‘80s. None of those contrasts are significant in German.


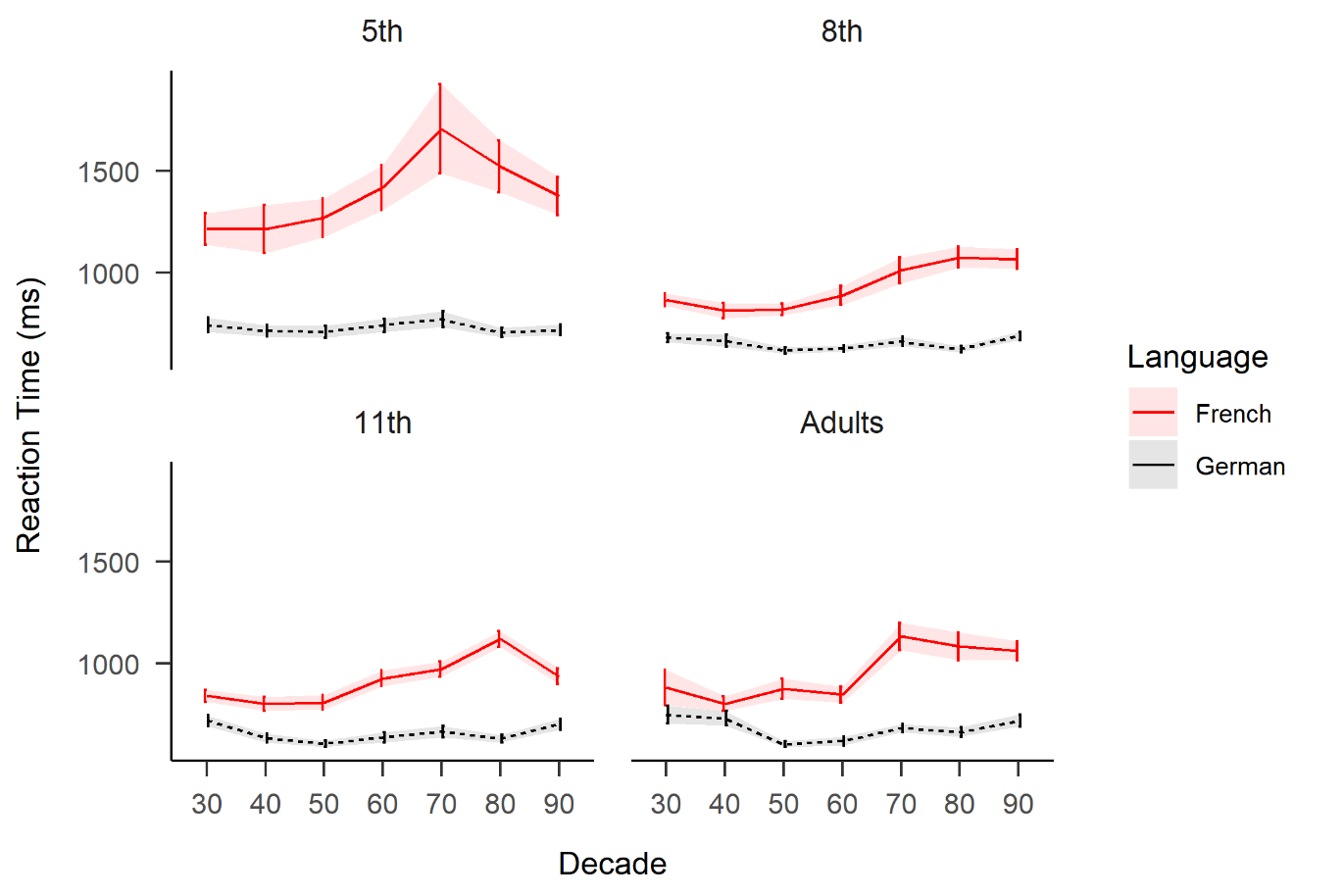


S4 Fig1. Mean reaction time of the reading aloud task for each decade at each age groups. Ribbons represent one standard error.

## Verbal-visual matching

| S4 Table 3: results of the linear mixed model *per decades* | | | | |
| --- | --- | --- | --- | --- |
|  | num Df | den Df | F | Pr(>F) |
| Age | 3 | 91.40 | 50.99 | <0.001 |
| Language | 1 | 83.23 | 66.66 | <0.001 |
| Number Size | 6 | 20.21 | 6.62 | <0.001 |
| Age x Language | 3 | 85.79 | 15.35 | <0.001 |
| Age x Number Size | 18 | 2017.63 | 3.62 | <0.001 |
| Language x Number Size | 6 | 20.35 | 9.05 | <0.001 |
| Age x Language x Number Size | 18 | 2017.28 | 2.75 | <0.001 |

### “Stepwise” contrasts:

The following custom contrasts compare each decade with the previous and following one. For example ‘30 vs ‘40, ‘40 vs ‘50, etc. Contrasts are calculated on estimated marginal means, degrees of freedom from Satterthwaite approximation and p-values are Bonferroni corrected.

| S4 Table 4: Contrasts between subsequent decades | | | | | | |
| --- | --- | --- | --- | --- | --- | --- |
| French | | | | | | |
|  | contrast | estimate | SE | df | t.ratio | p.value |
| 5^th^ grade | 30 – 40 | -183.46 | 117.50 | 80.60 | -1.56 | 0.1223 |
|  | 40 - 50 | 114.62 | 119.90 | 87.00 | 0.96 | 0.3416 |
|  | 50 - 60 | -435.53 | 123.70 | 97.50 | -3.52 | 0.0007 |
|  | 60 - 70 | -381.76 | 130.40 | 119.40 | -2.93 | 0.0041 |
|  | 70 - 80 | -22.69 | 134.50 | 134.10 | -0.17 | 0.8663 |
|  | 80 - 90 | -17.12 | 131.60 | 124.10 | -0.13 | 0.8967 |
|  | | | | | | |
| 8^th^ grade | 30 – 40 | -173.54 | 105.6 | 52.8 | -1.644 | 0.1062 |
|  | 40 - 50 | 133.03 | 105.7 | 53.1 | 1.258 | 0.2137 |
|  | 50 - 60 | -117.65 | 105.5 | 52.8 | -1.115 | 0.2698 |
|  | 60 - 70 | -121.56 | 107.7 | 57.2 | -1.129 | 0.2636 |
|  | 70 - 80 | -183.97 | 107.9 | 57.8 | -1.705 | 0.0936 |
|  | 80 - 90 | -32.91 | 108.3 | 58.4 | -0.304 | 0.7623 |
|  | | | | | | |
| 11^th^ grade | 30 – 40 | -18.24 | 107.6 | 57.1 | -0.17 | 0.866 |
|  | 40 - 50 | 52.74 | 107.3 | 56.5 | 0.491 | 0.6251 |
|  | 50 - 60 | -67.36 | 110.4 | 63 | -0.61 | 0.5438 |
|  | 60 - 70 | -53.57 | 114.1 | 71.5 | -0.47 | 0.64 |
|  | 70 - 80 | -205.86 | 112.6 | 68 | -1.829 | 0.0718 |
|  | 80 - 90 | 30.54 | 112.4 | 67.8 | 0.272 | 0.7868 |
|  | | | | | | |
| Adults | 30 – 40 | -177.21 | 115.7 | 76 | -1.532 | 0.1297 |
|  | 40 - 50 | 61.05 | 115.6 | 75.7 | 0.528 | 0.5988 |
|  | 50 - 60 | -100.44 | 115.6 | 75.7 | -0.869 | 0.3877 |
|  | 60 - 70 | -58.69 | 116.1 | 76.7 | -0.506 | 0.6146 |
|  | 70 - 80 | -135.44 | 118.4 | 82.7 | -1.144 | 0.256 |
|  | 80 - 90 | 109.33 | 121.5 | 91.8 | 0.9 | 0.3705 |
| German | | | | | | |
| 5^th^ grade | contrast | estimate | SE | df | t.ratio | p.value |
|  | 30 – 40 | 63.01 | 103.4 | 76.8 | 0.61 | 0.5439 |
|  | 40 - 50 | -245.4 | 104.2 | 79.2 | -2.355 | 0.021 |
|  | 50 - 60 | 161.73 | 104.1 | 78.8 | 1.554 | 0.1242 |
|  | 60 - 70 | -50.87 | 102.7 | 74.7 | -0.496 | 0.6216 |
|  | 70 - 80 | -83.95 | 103.1 | 75.9 | -0.815 | 0.4178 |
|  | 80 - 90 | 79.17 | 104 | 78.7 | 0.761 | 0.4489 |
|  | | | | | | |
| 8^th^ grade | 30 – 40 | -115.7 | 99.3 | 65.5 | -1.165 | 0.2483 |
|  | 40 - 50 | 183.35 | 99 | 64.6 | 1.853 | 0.0685 |
|  | 50 - 60 | -119.82 | 99.4 | 65.7 | -1.205 | 0.2325 |
|  | 60 - 70 | 168.74 | 98.9 | 64.4 | 1.706 | 0.0928 |
|  | 70 - 80 | -202.7 | 98.2 | 62.7 | -2.064 | 0.0432 |
|  | 80 - 90 | -10.12 | 99.5 | 66.1 | -0.102 | 0.9193 |
|  | | | | | | |
| 11^th^ grade | 30 – 40 | 47.55 | 103.4 | 76.9 | 0.46 | 0.6469 |
|  | 40 - 50 | 37.87 | 103.5 | 77.2 | 0.366 | 0.7155 |
|  | 50 - 60 | -91.99 | 103.7 | 77.8 | -0.887 | 0.3776 |
|  | 60 - 70 | 56.91 | 102.1 | 73.3 | 0.557 | 0.579 |
|  | 70 - 80 | -43.62 | 100.7 | 69.4 | -0.433 | 0.6663 |
|  | 80 - 90 | -31.17 | 101.9 | 72.5 | -0.306 | 0.7605 |
|  | | | | | | |
| Adults | 40 | -33.83 | 106.9 | 87.6 | -0.317 | 0.7523 |
|  | 50 | 88.15 | 106.9 | 87.6 | 0.825 | 0.4116 |
|  | 60 | -88.4 | 106.9 | 87.6 | -0.827 | 0.4103 |
|  | 70 | 71.96 | 107.9 | 90.8 | 0.667 | 0.5064 |
|  | 80 | -81.46 | 108.8 | 94 | -0.748 | 0.456 |
|  | 90 | -1.27 | 108.9 | 94.2 | -0.012 | 0.9907 |

In French, ‘30s, ‘40s, ‘50s numbers have comparable RTs. However, ‘60s have significant faster RT than ‘70s for 5^th^ graders and ‘70s are faster than ‘80s fro 8^th^, and 11^th^ graders. No differences were found for adults. In German the only significant differences found were between ‘40s and ‘50s in 5^th^ graders and between ‘70s and ’80s in 8^th^ graders.


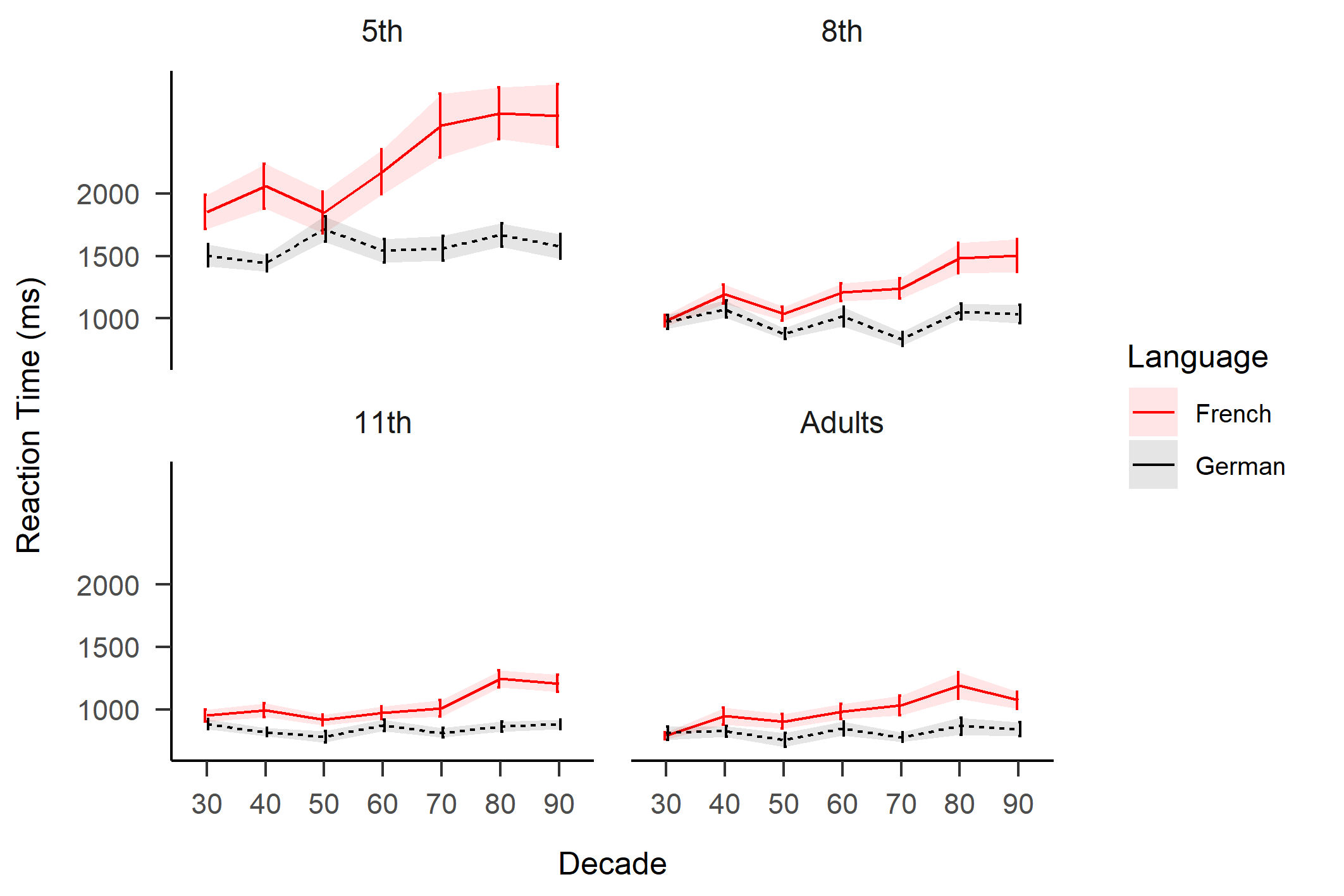


S4 Fig. 2: Mean reaction time of the verbal-visual matching task for each decade at each age groups. Ribbons represent one standard error.
