# Supplementary material for: Number transcoding in bilinguals—A transversal developmental study
Source: PLoS One. 2022 Aug 29;17(8):e0273391. doi: 10.1371/journal.pone.0273391 (PMC9423630; doi:10.1371/journal.pone.0273391)
Supplement: Attachment — Submitted filename: Response to Reviewers.docx [file pone.0273391.s005.docx]

# Reviewer #1

Dear Editor, Prof. Dr Prado,

dear Reviewer,

We would like to thank the editor and the two anonymous reviewers for their very valuable comments and their constructive suggestions. As synthesized by the editor, two major points were raised by the reviewers: the first point(s) concerned the theoretical framework in general, and the second the statistical analysis applied to the dataset. In the revised version of the manuscript we have addressed these concerns in depth and believe the manuscript’s quality has greatly improved as a consequence. In the following, we describe how we implemented the different points raised by both reviewers into the revised manuscript. Below you can find our answers (A) to the comments (C) to each of the two reviewer’s points. Please note that adjustments in the manuscript are indicated in this colour code.

**C1:** *The theoretical framing is poor, unfocused and sometimes incorrect.*

**C1.1:** *I do not refer to the important/impact of the study, because this is not a publication criterion in PLOS ONE. However, irrespectively of the question impact, the manuscript should still interpret the result in terms of relevant theory.* *There is no reference to relevant cognitive models of number processing, although there are quite a few of those (McCloskey; Dehaene & Cohen; Butterworth & Cipolotti; Dotan & Friedmann; in a way, also Huber, Nuerk, & Willmes; and more).*

**A1.1:** We agree with the reviewer’s suggestion that the manuscript was not sufficiently considering the different cognitive models that are relevant for understanding number processing and transcoding in particular. To complete the theoretical framework with the above mentioned transcoding models, we added three paragraphs in the introduction describing cognitive models of transcoding (**lines 51-77**):

Several cognitive models have been proposed to describe transcoding, with some also taking into account cognitive development or the acquisition of a second language. These models are summarized below in two main categories: semantic and asemantic (Barrouillet et al., 2004).

In semantic models, transcoding requires an obligatory access of the number’s magnitude. For example McCloskey (McCloskey, 1992; McCloskey et al., 1985) proposed a transcoding model in which the entry number - regardless from the input’s format - accesses an abstract semantic representation from where it is then handled into a production system. Power and Dal Martello (Power & Dal Martello, 1990) further proposed a model specifically for number dictation (*i.e.* verbal to Arabic format) which differs from the previous one in that semantic representations reflect the verbal word structure of the numbers. In sum for these models, transcoding activates a common semantic representation, independently of language or age.

In contrast, asemantic models propose that numbers can be transcoded without accessing magnitude. Deloche and Seron (Deloche & Seron, 1982) proposed such a model for number naming (*i.e.* Arabic to verbal) where the first step involves parsing the input into primitives which are then submitted to a set of rules for the output system. Later on, Barrouillet and colleagues (Barrouillet et al., 2004) proposed ADAPT (A Developmental Asemantic Procedural Model), which includes the developmental perspective that frequent number words become lexicalized and are thus directly retrieved from long term memory, hence bypassing the procedural processes. Another more general model considering number representations is the Triple Code Model (TCM). The TCM proposes a functional and topographical framework of how and where in the brain approximate, visual symbolic and verbal codes are processed. Approximate processing of magnitudes (*i.e.* not exact quantities) are taken over by the horizontal segment of the intraparietal sulcus. Visual symbols, depending on visuospatial abilities, are processed by the parietal and fusiform gyrus (number form area). Finally, verbal codes, depending on linguistic processes, involve the left angular gyrus of the temporal-parietal gyrus (Dehaene et al., 2003). Therefore, the TCM implies that transcoding from or to a verbal code does not require semantic magnitude activations and is language-dependent (Dehaene, 1992). More recently, Dotan and Friedmann (Dotan & Friedmann, 2018) proposed a model for Arabic to verbal transcoding where numbers are first visually analysed for identity, order and decimal structure and then passed to a second system which applies language-specific rules which are associated with their phonological and articulatory counter-parts. In sum, these asemantic models sustain that semantics are not necessarily activated for transcoding, some including developmental perspectives and others language differences.

In addition we added these paragraph in the discussion, in order to link the cognitive models presented in the introduction with the results that were found **(lines 497 to 510)**:

From the perspective of cognitive models presented in the introduction, the cost for *transparency of power* in French would fit with Power and Dal Martello’s semantic model if rules reflecting the vigesimal form are added, since more rules would mean slower production (Power & Dal Martello, 1990). Taking into account development, the asemantic ADAPT model (A Developmental Asemantic and Procedural model for Transcoding (Barrouillet et al., 2004)), would partly fit with our results. ADAPT was proposed for number dictation (*i.e.* verbal to visual Arabic transcoding), hence similar processes could have taken place in the verbal to visual matching and reading aloud tasks (*i.e.* visual Arabic to verbal) of the present study design. In ADAPT, French `70s, `80s and `90s require more rules and therefore also more processing time. However, the model further proposes a lexicalization through repetitions over development, *i.e.* leading to faster transcoding of `70s, `80s and `90s. Yet, when standardizing age groups variability through z-score transformations, we noted that numbers above 70 were similarly slower to transcode in all age groups, arguing against a lexicalization with training. The non-lexicalization of `70s, `80s and `90s vigesimal numbers in French might interact with their late formal acquisition (*i.e.* LM2 acquired at 7^th^ grade, around 11-12 years old). Finally, the cost for `70s, `80s and `90s numbers could be accounted in Dotan and Freidman’s recent transcoding model (Dotan & Friedmann, 2018) at the phonological level of binding between digits and number words.

Despite being a model about number dictation, we also added the ADAPT model. First, because it was recommended by the second reviewer. Second, it is the only transcoding model at our knowledge that includes a developmental perspective, which is one of the approaches of our study. Third, a recently published article with a similar verbal visual matching task than our study (Steiner et al., 2021) (p.15) included the ADAPT model and we adapted this article’s reasoning that is generally speaking a model for verbal to Arabic digit’s transcoding, thus implying common processes are probably required in our tasks too.

**C1.2:** *The topic of this article is number transcoding, but for some reasons the authors chose to dedicate much of the theoretical discussions to other issues, which are only vaguely related – ANS, arithmetic, and language. Perhaps the two most extreme examples for this is that (1) the first page of the manuscript (!!) is simply irrelevant – except the last few lines, it talks mostly about ANS and brain regions, two topics that are only vaguely related to the article’s main point.* *(2) The first sentence in the abstract is simply incorrect. It says that the study examined the “analogic, verbal, and visual symbolic codes”, but the “analogic code” (ANS) was not really examined here.*

**A1.2:** We agree with the reviewer’s critique about the length of the first page. Our original aim was to refer to the triple code model of Dehaene, underlying the possible multiplicity of verbal codes. We see now that it is over-emphasized and the approximate code is irrelevant, especially for the introduction. Hence, we have reduced the first page’s content to a paragraph, removing the passages concerning approximate representations (**lines 42-48**).

Exact numerical representations are supported by symbolic verbal (*e.g.* forty-two) and visual (*e.g.* 42) representations which are acquired through learning. However, in many languages the verbal number word’s syntax differs from the visual one, *i.e.* the Arabic number system (Chrisomalis, 2010; Ifrah & Bellos, 2000). This difference might have an influence on transcoding, *i.e.* the cognitive transformation from one code to another. Thus reading aloud for instance the number “42” implies the transcoding from a visual (“42”) to a verbal code (“forty-two”). Moreover, in the case of bi- and multilingualism, acquiring multiple languages means that during development multiple verbal codes are mapped with the visual code (Salillas & Martínez, 2018).

Accordingly, the first sentence of the abstract was also adjusted (**lines 21 to 23)**

Number transcoding is the cognitive task of converting between different numerical codes (*i.e.* visual “42”, verbal “forty-two”). Visual symbolic to verbal transcoding and *vice versa* strongly relies on language proficiency.

**C1.3:** *Similarly, the selection of cited literature seems a bit odd throughout most of the manuscript (but in the Discussion it is good). There is a lot of published work on transcoding, but the article ignores much of this literature and cites literature from other domains, which is less relevant or hardly relevant. There is no citation at all of many relevant works by researchers who did a lot of theoretical work on transcoding, e.g., Michael McCloskey, Carlo Semenza, Margaret Delzaer, Marie-Pascal Noël, Xavier Seron, Laurent Cohen and more – including works that specifically addressed the base-10 vs base-20 issue. Even when talking about arithmetic, there are additional studies that specifically examined the role of the verbal structure of numbers and could be cited (e.g., Campbell & Clark, 1992; Gonzalez & Kolers, 1982; Noël & Seron, 1997).*

**A1.3:** We thank the reviewer for her/his suggestion concerning the literature. We added several additional references such as McCloskey [7] and Seron [10]. However, we decided not to add all the proposed references, since the aim of the present study was not to compare results between developmental and neuropsychological literature.

**C1.4:** *On few occasions, the manuscript cited general language literature as a reference to number-specific phenomena, e.g. references [49] [52]. I would prefer seeing citation of studies more specific to numbers.*

**A1.4:** We agree with the reviewer’s suggestion to remain focused on literature concerning numerical cognition. We deleted the references from outside the field of numerical cognition, since these are not strictly necessary (**lines 176 to 177**):

However, they agree with recent reports from the bilingualism literature that academic knowledge acquired in one language is not (or difficultly) transferable to the other language (Volmer et al., 2018).~~,60,61~~].

**C2:** *The article’s main theoretical question requires the comparison of base-10 versus base-20 verbal numbers. To operationalize this, the authors took the French numbers above 60 as reflecting the base-20 system. This does not seem justified. In French, the syntactic structure of verbal numbers in the 60’s and 80’s is congruent with both systems (b10 and b20). Only numbers in the 70’s and 90’s are clear examples for base-20 syntax. I would guess that if you focus only on the 70’s and 90’s, your results will actually improve. Also, perhaps you may even be able to show that the 80’s are as easy as the smaller numbers – this could be a nice control for an artefact of number size (as opposed to syntactic structure, the effect you’re actually interested in).*

*The manuscript seems to assume that numbers in the 80’s are cognitively represented as “20 times 4 plus X”, e.g., 83=20x4+3. Presumably, the reason is that the French word for 80 is “quatre-vingt”, “four-twenty” (although this is not explained explicitly). Although this argument has been made in past studies, it is not convincing at all – “quatre-vingt” is very likely to be one lexicalized value, even if its etymology is 20x4. If there is empirical evidence showing that 80 is represented in a decomposed manner in the relevant population, please cite it. If not, please remove this claim, or at least present it as an unsupported hypothesis.*

*Note that even if you view 80 as decomposed 20x4, this still does not mean that the numbers 81-89 are in base-20, because the issue of 80 vs. 20x4 concerns only the tens word, not the way it is composed with the ones word.*

**A2:** We thank the reviewer for this very valuable suggestion. We agree that strictly speaking ‘80s (*quatre-vingt* 4*20) and ‘90s (*quatre-vingt-dix* 4*20+10) are clear representations of a base-20 system. On a conceptual level, ‘70s (*soixante-dix* 60+10) are not in base-10 neither, hence we used the term “vigesimal” to refer to these particular number word structure that distinguishes French from German. The question if base-20 are transcoded from algorithmic or procedural mechanisms (*i.e.* *20 times 4 plus X”, e.g., 83=20x4+3*) remains theoretical. Certain transcoding models (*i.e*. Power Dal Marelo, Seron, Barrouillet) propose it might be the case, at least for very young children. This would explain the higher rate of transcoding errors in (Seron & Fayol, 1994), and the prolonged reaction times in (Van Rinsveld & Schiltz, 2016).

Accordingly, we decided to adjust our analyses to make a slight change regarding the comparisons between numbers over and under 60. Instead, we dropped the ‘60s (as it is in base-10) and compared ‘30s, ‘40s, ‘50s vs ‘70s, ‘80s, ‘90s. This approach is supported by additional analyses (see annex), indicating that ‘60s were not significantly different than ‘50s, and hence the cognitive cost related to processing vigesimal numbers in French is operating from ‘70s (“soixante-dix”) to ‘90s (“quatre-vingt-dix”)^[1]^. Hence, also figures and tables were updated.

Since the question if ‘80s are as fast to transcode as smaller numbers is an empirical one, we tested this statistically. In the annex, we compared the different decades to test the hypothesis of (a) are ‘60s faster than ‘70s (see previous paragraph) and (b) are ‘80s (“quatre-vingt”) faster than ‘70s and ‘90s. This allowed contrasting each decade with respectively previous and following one, to spot a “statistical jump” in terms of RT. As a result, we noticed that ‘60s are indeed not significantly different from ‘50s. Moreover, ‘80s were also not significantly different from ‘70s and ‘90s in both tasks. Note that for the reading aloud tasks in 5^th^ and 11^th^ graders, ‘80s and ‘90s were almost significantly different (z = 1.96, p = .05), probably due to number words lengths (*i.e.* 87:*“*quatrevingtsept” vs 97: “*quatreving****dix****tsept*”). Hence, the majority of these comparisons seem to refute the hypothesis that “*quatre-vingt*” (80) are lexicalized.

**C3**: *The theoretical interpretation in the discussion is not convincing and should be improved.*

**C.3 (a)** *The theoretical interpretation in lines 560-575 seems incorrect in several ways.*

*-* *Lines 560-562: this is not in good agreement with commonly-accepted cognitive models of number production (e.g. McCloskey et al 1986). Phonologically, both “quatre-ving” and “dix-sept” are probably single lexical entries so these are two words, not four. I am not sure this was tested in French, but certainly you cannot claim the opposite as if it was a fact.*

**-** *Even if we assume that French 97 requires activating 4 lexical entries, it is still not clear why the cost should be attributed specifically to interference (and not, for example, to the effort arising from the need to retrieve more words).*

**-** *Lines 566-568, reference [58]: the example is completely irrelevant, and in this context, even a bit misleading. Of course, some cognitive interference effects exist. But it doesn’t mean that this is the case for numbers too. Especially since numerous studies show fundamental dissociations between the production mechanisms of number words and non-number words.*

**A.3 (a)** We thank the reviewer for this suggestion and agree that it is probably too speculative to state that the two words building “*quate-vingt”* require multiple lexical activation’s generating lexical interference. We have therefore depleted this interpretation from the discussion. We have restated the interpretations by referring more explicitly to cognitive models of transcoding in these rewritten paragraphs. We also designated more explicitly some possible links across the numerical cognition and psycholinguistic domains (**lines 497-519)**

From the perspective of cognitive models presented in the introduction, the cost for *transparency of power* in French would fit with Power and Dal Martello’s semantic model if rules reflecting the vigesimal form are added, since more rules would mean slower production (Power & Dal Martello, 1990). Taking into account development, the asemantic ADAPT model (A Developmental Asemantic and Procedural model for Transcoding (Barrouillet et al., 2004)), would partly fit with our results. ADAPT was proposed for number dictation (*i.e.* verbal to visual Arabic transcoding), hence similar processes could have taken place in the verbal to visual matching and reading aloud tasks (*i.e.* visual Arabic to verbal) of the present study design. In ADAPT, French `70s, `80s and `90s require more rules and therefore also more processing time. However, the model further proposes a lexicalization through repetitions over development, *i.e.* leading to faster transcoding of `70s, `80s and `90s. Yet, when standardizing age groups variability through z-score transformations, we noted that numbers above 70 were similarly slower to transcode in all age groups, arguing against a lexicalization with training. The non-lexicalization of `70s, `80s and `90s vigesimal numbers in French might interact with their late formal acquisition (*i.e.* LM2 acquired at 7^th^ grade, around 11-12 years old). Finally, the cost for `70s, `80s and `90s numbers could be accounted in Dotan and Freidman’s recent transcoding model (Dotan & Friedmann, 2018) at the phonological level of binding between digits and number words.

Speculatively, it could be argued that for French morpho-syntactically complex number words that are not lexicalized, multiple additional morphemes need to be retrieved (see *i.e.* (Meeuwissen et al., 2003)). This could be the origin of the cognitive slow-down as it requires to retrieve more lexical morphemes (*i.e.* four for 97: “quatre”, “vingt”, “dix”, “sept”). Eventually these morphemes, which are made of other numbers might interfere among each other, in a similar fashion as for multiplications (*i.e.* proactive interference (Campbell, 1995; De Visscher & Noël, 2014)).

Please note that all three models would also generally predict slower responses for German number words above 12 due to the additional inversion rule in German. Such effects of *transparency of order* have been found in previous studies (Poncin et al., 2019; Steiner et al., 2021; Zuber et al., 2009), but it is possible these were masked in the present study by the effect of language of math acquisition described in the following section.

An also at lines (565-571):

The LM2 cost is also explainable from a psycho-linguistic perspective: here we present a syntactic and a lexical interpretation. Syntactically, the LM2 cost might result from an over-generalization of the LM1 syntactic *inversion* rule (see for example (Zuber et al., 2009)): transcoding number words in French would require inhibition of the inversion rule (over)learned from German. A lexical explanations can be found in more general bilingual models such as the bilingual interactive activation model (BIA+), predicting a response competition between both languages, with faster activation for the more frequently used language (Dijkstra & van Heuven, 2002). In this framework, the slower lexical retrieval of LM2 would also have detrimental impacts on short term memory (Ellis & Hennelly, 1980; Gathercole & Baddeley, 1993) and hence arithmetic (Friso-van den Bos et al., 2013).

**C.3 (b)*: “****Lines 576-598, 623-629: it seems strange to interpret your findings according to the ADAPT model, because ADAPT is a model for number writing (verbal-to-arabic transcoding), and the task here is reading (arabic-to-verbal transcoding). You can use models of number reading instead.
I don’t think this should disturb you because you don’t really rely on any particularity of the ADAPT model, you merely rely on the well-accepted, widely-supported distinction between lexical processing and syntactic processing in numbers. What your data shows is probably a phenomenon that was shown in many previous studies: numbers with complicated syntactic structure are harder to process than syntactically-simple numbers, and syntactic ability develops with age. I think it would be more appropriate to frame your claim in these more-general terms, rather than present it as if the results support a particular cognitive model – I don’t think they do. If I were you, I would just drop the interpretation in lines 560-575, stick with the second interpretation, and present the results as evidence for a syntactic-complexity effect (a conceptual replication of several previous studies), and for the development of syntactic abilities with age. But needless to say, this is of course totally your decision.
Either way, I think that the manuscript will benefit from using the terminology “number syntax”. Although sometimes vague, this is standard terminology (certainly more standard than “transparency of power”) and it seems appropriate for your research question.”*

**A.3 (b):** We thank the reviewer for the suggestion. Given the well-funded request to provide a more thorough theoretical embedding of our work (e.g. C1), we decided to keep the ADAPT model as an interpretation of the results, since the model proposes a verbal to visual transcoding (that is involved in our tasks). For this approach we also aligned with recent studies on transcoding adopting a similar verbal-visual matching paradigm (Steiner et al., 2021) .

**C.3 (c)** *Lines 613 seem like a vague mixture of several phenomena/issues. I found it more confusing than clarifying.*

**-** *Lines 613-614: your findings do not seem to be about language switching, but about familiarity with language. While I agree that language switching is probably relevant, I find this analogy more blurring than clarifying. At least discuss the difference between the two phenomena*

**A.3 (c):** We agree with the reviewer’s suggestion. Hence, we have adapted the following paragraph (**lines 535 to 543)**

Our findings are in line with studies reporting qualitatively different arithmetic performance with LM+ compared to LM- in bilingual Filipino-English and Spanish-Basque participants, even if LM- corresponds to participants’ mother language (Bernardo, 2001; Salillas & Wicha, 2012). Finally they also match and extend the finding that solving addition problems was slower in LM2 than LM1 in participants coming from the same education system than in the present study (Van Rinsveld et al., 2015).

Interestingly, while the *language of math acquisition* impacted the number reading task, this was considerably less the case in the verbal-visual matching task. Lexically, the different pattern of results for the LM2 could be explained by different memory retrieval mechanisms (Vander Beken & Brysbaert, 2018) since the number reading task can be considered as a form of free recall while the matching task is more similar to a familiarity judgment.

And lines 576-577:

A limitation of the present study is that it did not allow to disentangle the role played by the *language of math acquisition* from the familiarity with number words in the two languages.

**-** *619-620: the encoding complex model makes very specific assumptions about the types of information coded in language-specific way (in particular arithmetic facts). The language effect you have found doesn’t seem to support these assumptions – it merely shows that language, and familiarity with language, matters. Frankly, this effect is a bit trivial, and pretty much agrees with any number-processing model I am aware of.*

**A.3 (c):** We agree with the reviewer’s suggestion and have therefore deleted the following lines: (lines 517)

~~These costs support the~~ **~~Encoding Complex Model~~** ~~suggesting that numbers might be encoded in a language and format-specific manner [70]~~

**C.3 (d)** *Lines 630-638: your data doesn’t seem to support working-memory effects in any way. Certainly, difficulty in lexical retrieval does not necessarily imply a WM issue.*

**A.3 (d):** We thank the reviewer for this suggestion. While the present study did not indeed address the role of working memory, the relation between transcoding and short-term memory is sustained by several studies (Camos, 2008; Imbo et al., 2014; Pixner et al., 2011; Steiner et al., 2021; Zuber et al., 2009). We therefore included a brief mention to working memory in the discussion, as a potential link between transcoding and arithmetics.

**C.4** *In the results section, some of the statistical analyses are described dryly, without any meaning/interpretation****.*** *Please add their theoretical implication. E.g. in p.15, the precise pattern of interactions with age seem to strengthen your theoretical claim about a progression of learning – why not say it explicitly right away? Especially in the summary paragraphs (lines 406-410, 495-503) I would expect that the text doesn’t just talk about factors and effects but also say something about the cognitive implications.*

*Even when the results are interpreted, this is sometimes done separately from the result. E.g. the analyses of both tasks start by saying that all double interactions were significant, but these interactions are explained only later in the text. Please fix: unite each result with its interpretation.*

*The comparison of the two tasks doesn’t seem to have any theoretical relevance and should probably be deleted (or at least pushed down to somewhere less central in the text). As far as I understand, there is no theoretical reason to assume that the performance in the two tasks would be similar, and we don’t learn any insights from this comparison.*

**A4:** We agree with the reviewer’s suggestion and because of the change of statistical analyses (LMM instead of ANOVA) we have rewritten the result section taking into account these suggestions. First, we have taken into account the unification between interpretation within the results (see Results, p.12 to 19).

Second, we have dropped the statistical comparison between the tasks. By removing the following lines (**line 180**)

~~While transcoding involves the search within a full set~~ ~~of lexical information in the production task, the recognition task only requires an already cued search amongst a limited number of response alternatives, which is largely based on familiarity processing [52].~~

**C5:** *Some of the terminology used in the manuscript is imprecise, non-standard or incorrect.*

*-* *“Language of math acquisition” is a confusing and imprecise term. You did not manipulate the language of acquisition (=learning), you manipulated the language in which the experiment was done. But I certainly agree that the reason for this effect is likely to be LM1 vs. LM2*

**-** *The first task is neither “a production task” nor “number naming”, it’s reading aloud (it includes both visual input and verbal production). The second task would be better described not as a recognition task, but as a matching task.*

***-****“Transparency of power” is not a standard term in the number transcoding literature, so please explain it before using it for the first time. Perhaps avoid using it in the abstract.*

**C5** We agree thank the reviewer for her/his suggestion. Despite the fact that language of mathematical acquisition was not experimentally manipulated, we kept this wording, as it is in line with previous work on arithmetic problem solving in the same multilingual educational context (Van Rinsveld et al., 2015, 2017). Throughout the paper, we have adapted the terminology for the two tasks according to the reviewers’ suggestions (*i.e.* from production task to reading aloud task and from recognition to verbal-visual number matching task. In general throughout the paper we also tried to reduce repetitions and shorten paragraphs (for brevity, these changes are not listed below, but can be found in the revised paper). The term “transparency of power” we also clarified, by explaining it in the abstract, and referencing its previous use in a taxonomy for the different effects of language on transcoding (Bahnmueller et al., 2018; Dowker & Nuerk, 2016) (see also line 91).

**-** *When saying the performance is better in condition X, say better than what (e.g. p. 17 line 407)*

**-** *The inversion in German is not between tens and units, it is between tens and ones (“units” = digits, “ones” = words).*

Thank you for this suggestion, we have accordingly modified the following sentence (**lines 95-97)**.

Another morpho-syntactic difference concerns the inversion between tens and ones as for example in German and Dutch (*i.e. y +* 10**x*).

**-** *Lines 579-581: there is a confusion here between two meanings of the term “semantic”. In the ADAPT model (and in other models, e.g. McCloskey’s), “semantic” often refers to a central abstract representation of the number. The number size effect refers to semantics as the quantity associated with the number via the ANS. These are two completely different things.*

Thank you for noting this, it was not our intention to link both terms for semantics, due to possible confusion between the meanings of semantics, we deleted those lines.

**-** *“Analogic code” is a correct/acceptable term for ANS, but it is not standard. “Approximate” would be a more standard terminology*

We substituted this terminology throughout the paper, adopting the more standard one (lines 66-69)

The TCM proposes a functional and topographical framework of how and where in the brain approximate, visual symbolic and verbal codes are processed. Approximate processing of magnitudes (*i.e.* not exact quantities) are taken over by the horizontal segment of the intraparietal sulcus. Visual symbols, depending on visuospatial abilities, are processed by the parietal and fusiform gyrus (number form area).

**C6:** *I would recommend using linear mixed model instead of ANOVA. It is more reliable, and also might improve your statistical results.*

**A6:** We have re-written anew the result section, since we changed the analyses from ANOVA to linear mixed models. However, since the experiment was not designed for LMM, we first had to test different models because to assure convergence of the models. The model that could be fitted for both tasks RT’s includes per-item random slopes and intercepts for each language, but only random intercepts per subjects. As suggested *(“70 makes much more sense than 60 though*”) we adjusted the analyses to compare `30s, `40s and `50s vs `70s, `80s and `90s numbers, instead of over and under 60. (see Results section, pages 12 to 19).

**C7:** *The quality of writing should be improved.
- The manuscript should undergo an English language review.
- The text is too long, please write more concisely. Just to give a few examples:
(1) The issue of French>60 examines transparency of power is explained too many time.
(2) The first paragraph in the results section can be cut into a short sentence. But there are many more.*
*(3) The text describing the analyses with (p. 16) and without (p.15) z-scored are redundant and tiring. You can write the text once, and say you got essentially the same results with and without z-scores (with results of both ANOVAs in a table). Similarly, fig. 6 is redundant and can be removed. Same issue for recognition task.*

*But I emphasize that these are just examples, please write the whole text more concisely*

**A8:** Throughout the paper we have thoroughly revised the English language and formulations, aiming to remove redundancies and formulate some paragraphs more concisely. For example we have shortened the te first paragraph in the *Transparency of power* section (lines 92-95):

In many Asian languages (*i.e.* Mandarin Chinese, Vietnamese) the morpho-syntactic structure of the verbal number system is highly consistent with the Arabic number system, and in general with the base-10 (*i.e.* 10**x + y* , see (Miura et al., 1988)). This linguistic characteristic, also termed *transparency of power*, facilitates learning to count (Lê & Noël, 2020; Miller et al., 1995; Miller & Stigler, 1987) and solving arithmetic problems (Geary et al., 1996; McClung & Arya, 2018; Rodic et al., 2015).

**C8** *Minor comments*

We thank the reviewer for the minor comments, we shortly comment them below :

**C8.1.** *The statistical analyses are described in detail, which is excellent, but because there are quite a few of them, I would recommend moving them to the results section. I completely lost track of the analyses while reading, and it was very hard to relate specific results to specific analyses.*

**A 8.1:** The whole result section has been rewritten anew.

**C8.2.** *Fig. 3 – the Y axis label says RT instead of accuracy*

**A8.2:** Thank you for pointing out this inadvertence error, which has been corrected.

**C8.3.** *Fig. 4 – instead of o60 and u60, perhaps write something clearer, e.g. >60, <60*

**A8.3:** we have changed the dataset subdivision (see above), hence now the comparison is between small (‘30s, ‘40s and ’50s) vs big (‘70s, ‘80s and 90’s) decades

**C8.4.** *Lines 344/355 – I think there is an unintended paragraph break here.*

**A8.4.** Thank you for pointing this out.

**C8.5*.*** *There are quite a lot of ANOVA results. Consider putting them all in table/s instead of in the text – it will cut the text shorter and could make it easier to find the relevant stats.*

**A8.6.** We have added tables with the results (see Table 1 to 4)

**C8.7*.*** *Lines 344-351: to show that the German and French trends are different with respect to >60 vs <60, here would be the correct place to talk about the Size x Language interaction.*

**A8.7**. The result section is completely rewritten anew.

**C8.8**. *Line 390: how many 4-syllable numbers are there? Also, as far as I know, the relevant measure for word length is typically assumed to be the number of phonemes, not the number of syllables (Nickels & Howard, 2004, Cognitive Neuropsychology).*

**A8.8.** We have followed the same procedure as in (Van Rinsveld & Schiltz, 2016)

**C8.9***. The particularity of the German language (tens-ones inversion) should be mentioned earlier on in the text.*

For the German number system, we added a description of transparency of power in the introduction (**lines 95 to 99**).

Another morpho-syntactic difference concerns the inversion between tens and ones as for example in German and Dutch (*i.e. y +* 10**x*). This linguistic characteristic, called *transparency of order* (Bahnmueller et al., 2018), explains some transcoding error patterns and reaction times (Clayton et al., 2020; Imbo et al., 2014; Moeller et al., 2015; Pixner et al., 2011; Poncin et al., 2019; Steiner et al., 2021; Zuber et al., 2009), gives rise to specific pattern of compatibility effects (Bahnmueller et al., 2015; H. Nuerk et al., 2005; H.-C. Nuerk et al., 2004) and complicates the solution of certain arithmetic problems (Göbel et al., 2014; Lonnemann & Yan, 2015; Xenidou-Dervou et al., 2015).

**A8.9.** We added a few lines in the introduction

**C8.10***. All figures: measurement units “millisec” should be written only on axis label, not in the figure caption.*

**A8.10** This has been modified accordingly

**C8.11.** *The introduction and discussion mention a transparency-of-power analysis for numbers above 70 but I think I didn’t see it in the results section, did I miss it?*

**A8.11.** In the present revised manuscript version we only use the cut-off from 70 onwards

**C8.12***. Line 556: as far as I can see this is incorrect - the base-20 cost was not independent of age, it decreased with age (even if it existed to some extent in all age groups).*

**A8.12.** We interpreted that the cost in French for `70s `80s `90s numbers was independently of age based on the z-scores. The reasoning being that the z-scores standardized the variability present in each age groups and since we do not find an interaction between Age and Size (see Supplementary Materials), it might mean that the results from the raw RT are biased due to bigger variance. This has now been clarified in lines 491 to 495.

Notably, the cost was found for both reading aloud and number matching tasks that is transcoding from visual to verbal and *vice-versa*. The results from the z-scores standardization suggest that the cost explained by a difference in *transparency of power*, generally and stably persisted from 5^th^ grade up to adulthood. In other words the cost maintains across age groups, tasks and the associated degree of language proficiency*.*

**C8.13***. The reference to ADAPT in line 577 is incorrect, [60] instead of [64]. Please double check all references.*

**A8.13** We thank the reviewer for noticing this. We have corrected the reference and checked that all references.

**C8.14***. Lines 180-182: this claim does not seem correct – at least, this is not the common assumption for how number transcoding works. Certainly, you cannot base this claim on reference [52] not from the number domain.*

**A8.14.** We thank the reviewer for noticing this. We have removed this interpretation.

# Reviewer #2

Dear Editor, Prof. Dr Prado,

dear Reviewer,

We would like to thank the editor and the two anonymous reviewers for their very valuable comments and their constructive suggestions. As synthesized by the editor, two major points were raised by the reviewers: the first point(s) concerned the theoretical framework in general, and the second the statistical analysis applied to the dataset. In the revised version of the manuscript we have addressed these concerns in depth and believe the manuscript’s quality has greatly improved as a consequence. In the following, we describe how we implemented the different points raised by both reviewers into the revised manuscript. Below you can find our answers (A) to the comments (C) to each of the two reviewer’s points. Please note that adjustments in the manuscript are indicated in this colour code.

**C1*:*** *line 44-46: Since multilinguals are outnumbering monolinguals across the globe [12], the question of how numbers are processed and transcoded in different languages is of crucial importance.’ Why? Undeniably there are a lot of multilinguals but that fact doesn’t necessarily make this particular research question crucially important. Please either remove or explain the relevance of the research question for all those multilinguals. This is a bit related to the discussion, see also later. If this is fundamental study that is perfectly fine, but don't pretend it also has immediate applications or benefits.*

**A1:** We agree with the reviewer’s suggestion concerning the applicability of the study’s outcome. In general, and this is also related to a later comment of the reviewer (see A13), we tried to reorient the formulations into a fundamental rather than applied relevance of our study. For example, as suggested by the first reviewer, by adding new paragraphs concerning transcoding models in both introduction (lines 51-77) and discussion (lines 477-491). Furthermore, we reformulated also why we think it is relevant to investigate transcoding in bilinguals (lines 48-50):

~~Given the importance of language in representing numbers comparing these additional~~ ~~transcoding routes is of crucial importance.~~ Since multilinguals are outnumbering monolinguals across the globe (Grosjean, 2010), the question of how numbers are processed and particularly transcoded using two (or more) verbal codes in a (developing) multilingual cognitive system is of crucial importance (Wicha et al., 2018).

**C2.1:** *line 75-78: This section would benefit from a clearer explanation of the different counting systems in German and French for those unfamiliar with those languages. Now the base-10 vs 20 is highlighted, but first I’d argue that the base 20 system in French is only visible for the 80s and 90s and not below (or else 62 would be 3x20+2 which is not the case). I’d say that the true base 20 starts at 80, as the 60’s follow the regular base 10 system and the 70’s are neither (as they are 60+10, 60+11 etc). And then there are the irregular number words for 11-16.*

*Line 77 ‘only eighty decades are vigesimal’ probably supposed to mean ‘only the decade 80’? Note by the way that also languages like English and French are not as transparent as Chinese in the base 10 system. If they were, a number like 60 in French would be ‘six dix’ (six ten) instead of ‘soixante’.*

*And please also elaborate on differences between the French and German counting system. The explanation of the base 10 vs 20 system is very elaborate but the decade unit inversion in German is barely mentioned, so short that I doubt whether a reader who is unfamiliar with languages that have this inversion even understands what it is. And what where the results from references [18] and [51]?*

*And I wanted to recommend some more papers on this phenomenon but I already found a few in the reference list without referencing to them in the text [65-67]. Was this part deliberately taken away to give the simplified impression that French numbers are always more difficult than German numbers? Decade unit inversion makes transcoding more difficult. This could become especially visible in the*

**A2.1:** We fully agree with the reviewer’s suggestion to improve the clarification of the French and German counting systems and their differences. Indeed, we had included a paragraph concerning the inversion in German in a draft version that we finally removed for reasons of conciseness in our last version (leaving by mistake the related references). While the effect of inversion in German is not directly investigated in our study (motivating our initial removal of the passage), we nevertheless agree with the reviewer that it should be mentioned for the sake of completeness. The following paragraph has been added to explain the inversion in German and related difficulties (lines 95-99).

Another morpho-syntactic difference concerns the inversion between tens and ones as for example in German and Dutch (*i.e. y +* 10**x*). This linguistic characteristic, called *transparency of order* (Bahnmueller et al., 2018), explains some transcoding error patterns and reaction times (Clayton et al., 2020; Imbo et al., 2014; Moeller et al., 2015; Pixner et al., 2011; Poncin et al., 2019; Steiner et al., 2021; Zuber et al., 2009), gives rise to specific pattern of compatibility effects (Bahnmueller et al., 2015; H. Nuerk et al., 2005; H.-C. Nuerk et al., 2004) and complicates the solution of certain arithmetic problems (Göbel et al., 2014; Lonnemann & Yan, 2015; Xenidou-Dervou et al., 2015).

Concerning the base-20 in French, we agree that the base-20 system is better represented in the ‘80s (*quatre-vingt* 4*20) and ‘90s (*quatre-vingt-dix* 4*20+10) than in the ‘60s. While the ‘70s (*soixante-dix* 60+10) are nor clearly in base-20, nor in base-10. However, from a conceptual level since ‘70s use teen numbers that are only used in another base-20 decade (‘90s) we believe they are more similar in their structure to ‘80s and ‘90s than ‘60s. In particular, when put in comparison with German. Hence we used the term “Vigesimal” to refer to this particular number word structure that distinguishes French from German. We now state this more clearly this in the below paragraph. We also thank the reviewer for correctly pointing out the lexical primitives that are present in French vigesimal numbers, we added one sentence specifying this characteristic (lines 100-108):

*Transparency of power* can also vary with a change of base, for example a base-20 system (20**x + y*), also called vigesimal. The use of the vigesimal system is found for example in French, Basque, or in Diola-Fogny (see (Haspelmath et al., 2005)). French, to be precise, has a mixed system since it uses base-10 and base-20 systems. Up to the `60s in French, like in English, the counting system follows the base-10 rule. However, `80s to `90s follow a base-20 system, *e.g.* 87 = *4*20 + 7,* “four-twenty-seven”. `70s are a particular case, as they are composed with `60s and teens numbers, *e.g.* 77 = 60 + 17, “sixty-seventeen”. Moreover, the teens (11 to 16) are lexical primitives, like in English and in stark contrast to the more transparent Asian languages. This system is subject to regional variances, for example in Belgian Wallonia only `80s are vigesimal (80, “quatre-vingt”, literally “four-twenty”) and in some Swiss-French cantons the vigesimal system is entirely absent (80, “huitante”*,* literally “eighty”).

**C3:** *The ADAPT model of number processing is mentioned in the discussion but perhaps this could be already done in the introduction, with an integrated approach discussing the difficulties of all languages.*

**A3:** We thank the reviewer for this suggestion. We agree about the importance of the ADAPT model and have added it in the introduction, integrated into a novel paragraph on different transcoding models (lines 62-65).

Later on, Barrouillet and colleagues (Barrouillet et al., 2004) proposed ADAPT (A Developmental Asemantic Procedural Model), which includes the developmental perspective that frequent number words become lexicalized and are thus directly retrieved from long term memory, hence bypassing the procedural processes.

**C4:** *p. 4 line 82. ‘Analogously’ but what follows is not analogously, it is in contrast.*

**A4:** We thank the reviewer for noting this ambiguity in this sentence’s formulation. We reformulated it follows (lines 112-113)

Further indicating an interaction between the number structure and number processing, Basque-speaking adults solved additions faster when the operand was in the base-20 structure.

**C5:** *The present study – Please, for a complete picture,* ***describe what language children in Luxembourg speak before going to school.***

*Luxembourgish, German, French? Many children speak Luxembourgish, that is highly similar to German, right? This may especially be relevant if parents stimulate number activities. I see that it follows in the methods section- a lot of different languages and French was excluded. Please elaborate on these choices and potential effects of this choice. In fact, the children with French as native language may form a very interesting group (although a bit small in size if there is age variation) to disentangle L1 from ML1 influences.*

**A5:** Concerning the language that children speak at home, this is probably mainly Luxembourgish, which is linguistically close to German as correctly noted by the reviewer. We fully agree on the plausible role of parental activity on numerical learning in the present samples. In the present study participant’s mother tongues (which are likely also their home-language) profiles were very diverse, with nonetheless a majority of Luxembourgish (64 out of 99), limiting the possible influence of different parental number language activity on transcoding. Here we excluded participants speaking French at home, since our study compared transcoding in German (i.e. LM1) and French (i.e. LM2). We reformulated the corresponding sentence for greater clarity (l278-280). We find the reviewer’s suggestion to analyze French mother-tongue participants very interesting (that is the participants who were excluded from the final sample). Unfortunately, the French sample was too small to consider each age group to obtain robust results (total 25, but 6 5^th^ graders, 4 8^th^ graders, 7 11^th^ graders and 8 adults). (lines 216-218)

From the initial full sample of 125, we first excluded participants who reported having French as their mother tongue, as these participants might have acquired French number words outside the context of formal schooling.

**C6.** *p.8 I understand how numbers below 60 are a purer indication of an effect of language of math acquisition (although confounded with decade-unit inversion) but this doesn’t stop at 60, sure numbers above 60 are also affected by this, not just by an effect of transparency of power.*

**A6.** We agree with the reviewer that the effect of language of math acquisition also concerns numbers above 60. Probably, the effect of language transparency and the effect of language of math acquisition are cumulative, such that vigesimal number words in French are slower because they are less transparent *and* they constitute the second language of learning mathematics. However, the effect of transparency of power is based on the difference within French, hence the effect of language of math acquisition should be factored out when comparing transparent (< 60) and non-transparent (>70) numbers (see also Fig.1 in the paper).

**C7:** *70 makes much more sense than 60 though, because in French only 70 and above cannot be transcribed by merely writing the numbers that you hear, the numbers in the 60s can. It is stated that the analyses were also performed with the cut-off at 70 but were there any relevant differences?*

**A7:** We agree with the reviewer’s suggestion that ‘70s is a better cut-offs than ‘60s. We used ‘60s as cut-off in the first version because we while hearing ‘60s, the participant cannot predict if it would have been the ‘60s or the ‘70s decade (*i.e.* starting with the same number words *soixante*) (Avanzi & Thibault, 2019). However, we agree that using a cut-off of 70 allows to better contrast numbers having distinct transparencies of power. In accordance with the reviewer’s suggestion, we directly therefore re-analyzed the data comparing ‘30s, ‘40s, ‘50s to ‘70s, ‘80s and ‘90s (see results section, and changes throughout the manuscript). The analyses with the cut-off at ‘70s led to similar patterns of results. Furthermore this approach is in line with the observation that transcoding of French ‘60s is not different in terms of RT than transcoding of ‘50s (while ‘70s are, see annex for additional fine-grained analyses where we compared each decade with each other)

**C8:** *Furthermore, the study make an attempt to identify effects related to the language structure and effects related to language of acquisition but the two are confounded while a control group is lacking. Any effect in favor of German can be due to L1/math language or to language structure. It would be helpful to compare the results to monolingual French and German control groups (and as mentioned above, the native French speaking children in Luxembourg), not just to mention this in the discussion but actually add those groups.*

**A8:** Regarding the suggestion to add a monolingual group as a control, we argue that this additional group would be difficult to compare with a bilingual group. Indeed, we think that including a monolingual group might also not be an ideal control because it would entail the comparison of different school systems and national curricula (Krinzinger et al., 2011). This difference might be additionally accentuated due to school systems that are designed for monolingual or bilingual teaching approaches. Monolingual German and French groups would also display differences in their transcoding patterns, especially in the younger age groups (see (Poncin et al., 2019; Steiner et al., 2021). Moreover, the individual cognitive level, the speed of lexical retrieval of monolinguals is faster than for bilinguals (even for the L1), which would cause different baselines (Gollan et al., 2005; Ivanova & Costa, 2008; Sullivan et al., 2018). This would add several additional layers of complexity to the study design and interpretation, which would go beyond the aims and scope of the present investigation.

**C9: *Design and statistical analyses****: it’s not explained what was done with separate stimuli.*

*How were aggregate scores determined?*

*Why not run a mixed effects model instead of ANOVA? The afex package that was used can handle that as well and you can estimate the effects more precisely because it can also handle random slopes across participants (and items). Not taking that into account can lead to alpha inflation.*

*Similarly for accuracy, which is bounded by 0% and 100%, ANOVA typically gives the wrong results, often spurious significant results but p-values can also be too low. Especially when values are close to the lower or upper boundary (which is the case with these very high accuracy rates close to ceiling) because ANOVA requires unbounded data. You need a model for proportional data such as a binomial mixed effects model in that case. See for instance Jaeger, T. F. (2008). Categorical data analysis: Away from ANOVAs (transformation or not) and towards logit mixed models. Journal of memory and language, 59(4), 434-446.*

*But also for the RT data a huge benefit of a mixed effects model is that you can enter all stimuli separately and you don’t need to calculate averages per condition, thus making maximal use of the available information.
Do note though that mixed effects models aren’t easy, and should be carried out appropriately with sufficient knowledge, or else the results may be off as well.*

**A9:** We agree with the reviewer’s suggestion that mixed linear model allow considering all available information optimally. Therefore, we followed this suggestion and completely re-analyzed our data using linear mixed models instead of ANOVA. We also applied binomial models for correct responses. Due to difficulties with convergence, we nevertheless had to remove the age group factor for the latter analyses. As suggested in C7 we adjusted the analyses to compare `30s, `40s and `50s vs `70s, `80s and `90s, instead of over and under 60. This led to similar patterns of results than with the ANOVA. Hence, we have rewritten the whole result section anew (see results section pages 12 to 19). Since the experiment was not designed for LMM, we first had to test different models because of issues with convergences. The model that could be fitted for both tasks' RTs includes per-item random slopes and intercepts for each language, but only random intercepts per subject.

**C10: *Analysis plan: explain the dependent variable.***

**A10:** We agree with the reviewer’s suggestion and added the dependent variables (lines 267-268)

The transcoding of two-digit numbers was compared in a transversal developmental design by Linear Mixed Models (LMM) on the dependent variables reaction time and correct responses.

**C11:** *Why speak of ‘groups of contrasts’ instead of just ‘contrast’? Moreover, I’m a bit confused about the terminology here because a contrast is applied to compare the levels of a single variable in a pre-specified way. For example, if you have 4 groups, comparing group A and B to group C and D, and then comparing group C to group D, that would be a planned contrast. In the context of the present paper, the term contrast seems to apply to just the main effect of a single variable (with only two levels). There is no need to specify all this. A three- or four-way ANOVA automatically gives the effect of each variable, and given that for most variables there are only two levels, this effect automatically tests if these two levels differ. No contrast necessary.*

*Only age has four groups so here a contrast could theoretically be applied, but this is not specified.*

*You do need to explain in the analysis plan which interactions you are going to test and how you are planning to proceed if the interaction is significant.*

*It is a common procedure if one interaction is significant, to decompose this interaction by going back one level, so if an interaction of A*B is significant you test the simple effect of B for each level of A. Or if A*B*C is significant, test interaction A*B at all levels of C.*

*p.13 why include an analysis with so many interaction terms if you ignore the results of those interactions anyway? You only look at one main effect. Note that many of the interactions are actually significant, meaning you can’t interpret a main effect, but you have to proceed with caution. Significant higher order interactions mean that the pattern is more complicated than any of the lower order interactions (or main effects) can capture, so their results can’t be interpreted isolated from the rest.*

*It would make more sense to decompose this significant four-way interaction by a series of follow up analyses: first three-way interactions, then two-way, as mentioned above. Along the way this automatically captures all the intended other effects (of language, number size, type of task and age).*

**A11:** We thank the reviewer these detailed suggestions regarding the statistical analyses. Since we have completely re-analyzed the dataset with linear mixed models, we have directly implemented the suggestions in the newly written result section (pages 12 to 19), were we compared the different interactions.

**C12:** *Then some inconsistencies like in line 332 ‘Finally, the triple interaction between age group, language and number size was significant for RT and CR’.
But the discussion says (line 556): ‘The results reveal that the cost entailed by processing base-20 numbers is independent of age and the associated degree of language.’
But it’s not independent of age, it becomes weaker over time, that’s what the three-way interaction showed. The cost did not disappear entirely but it did wane.*

**A12:** We thank the reviewer for noticing these inconsistencies between results and discussion. We based the interpretation that the transparency of power is independent of age mainly on the z-score analyses, since the difference among age groups might be due to higher inter-individual variability, which might be considered as “noise in the signal” (*i.e.* the high standard deviation in Grade 5). These variabilities might be due to developmental differences (*i.e.* in processing speed) rather than cognitive processing differences. While after z-score transformation, the interaction with age was not present. We added a sentence in the discussion to clarify this point (lines 505-507).

Yet, when standardizing age groups variability through z-score transformations, we noted that numbers above 70 were similarly slower to transcode in all age groups, arguing against a lexicalization with training.

**C13:** *Line 670 ‘Our results have practical implications’, this looks forced. What follows is very general ‘linguistic factors have to be taken into account when instructing and evaluating children’. That may be true but it doesn’t follow from this study, it may be more relevant then to look at international comparisons: does Luxembourg drop in rankings after children switch to French? Then I’d see the issue, but in this study, how bad is it if someone is a few milliseconds slower in writing down a certain number word? And if it is bad, how can a teacher take this into account when instructing and evaluating children and aren’t they doing that already? Please specify the practical implications of this particular study (or argue that this is a fundamental study and as such there are no practical implications, which is perfectly acceptable by the way. Not every study has practical implications).*

**C13:** We agree with the reviewer’s suggestion. As already mentioned in A1, we reoriented the interpretation to a more fundamental than applied research direction. Practically, we removed the following paragraph we had written having in mind the educational situation in Luxembourg, where the costs have also been found for arithmetic (Van Rinsveld et al., 2015).

~~Our results have practical implications, especially when aiming to provide equal opportunities for all students. Since linguistic factors can impact mathematics, these have to be taken into account when instructing and evaluating children. Considering that mathematical learning is cumulative, meaning that solid bases of previous knowledge are necessary to understand more elaborate concepts in mathematics, the impact of language on numerical and mathematical learning needs to be considered as early as possible [76]. Taken together the present results confirm and extend the interactions between language and number processing observed in more complex tasks such as arithmetic problem solving, corroborating the role of language in numerical cognition.~~

***Minor points***

**C13.1:** *p. 3 line 47-48: ‘The simplest declination corresponding to bilingualism’. What is declination supposed to mean here? Is it form perhaps?*

**A13.1:** We thank the reviewer for this suggestion. We changed the following lines accordingly: (line 78-80)

When investigating how the acquisition of several languages (and hence multiple number words) develops, the many forms and constellations of multilingualism must be taken into account; with bilingualism corresponding to the simplest instance.

**C13.2:** *p. 4 line 80 unusual use of ‘peers’, peers are children from the same environment, commonly classmates in a school context, not children from different countries.*

**A13.2:** We thank the reviewer for this suggestion. We changed the following lines accordingly: (line 108-109)

In a study comparing French-speaking 1^st^ graders from Wallonia and France with a number dictation task, more mistakes were committed in the `70s and `90s by the latter (Seron & Fayol, 1994).

**C13.3:** *P. 6 line 121 what is LM-? The language in which mathematics was not learned? Please define.*

**A13.3:** We thank the reviewer for noticing that this term definition was not complete. Hence we tried to be more clear about the definition of LM- (lines 146 to 151)

The LSC generalizes to more ecological learning settings, showing that mathematical problems are solved more accurately in LM+, even if it is not the dominant language (*i.e.* the LM+ not being the same language as the L1). For example, Bernardo (Bernardo, 2001) investigated arithmetic among high school Filipino-English bilinguals who have Filipino as L1 but specifically learned mathematics in English (LM+). The results indicate a cost for arithmetical problems written in number words in Filipino (*i.e.* being the L1 but LM-) compared to English (*i.e.* being an L2 but LM+), which in turn showed comparable results than for problems presented as Arabic digits.

**C13.4:** *Correct terminology is two-way or three-way interaction, not double or triple.*

**A13.4:** We thank the reviewer for noticing this terminological imprecision. We have substituted the terminology in the present paper (*i.e*. lines 312 and 331).

**C13.5:** *Line 659: ‘It is, however, noteworthy that math education extended for one year longer in LM2 (7 years of primary education) than in LM1 (6 years of secondary education), which might help to balance the frequencies in both languages.’ This only makes sense if the words primary and secondary were reversed. But it may help to explain the Luxembourgish situation in a bit more detail in the introduction.*

**A13.5:** We thank the reviewer for noticing this inversion. We have corrected it in the following lines (lines 578 to 580)

It is, however, noteworthy that math education extended for one year longer in LM2 (7 years of ~~primary~~secondary education) than in LM1 (6 years of ~~secondary~~primary education), which might help to balance the frequencies in both languages.

# Annex:

In the following additional analyses with linear mixed models, we compared all decades. That is, in the following, the factor *Number* *Size*, has **8 levels: ‘30s, ‘40s, ‘50s, ‘60s, ‘70s, ‘80s, ‘90s**. Otherwise, the analysis remains exactly the same as in the main manuscript.

## Reading aloud task:

|  | num Df | den Df | F | Pr(>F) |
| --- | --- | --- | --- | --- |
| Age | 3 | 94.51 | 17.15 | < 0.001 |
| Language | 1 | 21.21 | 682.32 | <0.001 |
| Number Size | 6 | 21.40 | 6.35 | <0.001 |
| Age x Language | 3 | 2320.04 | 111.10 | <0.001 |
| Age x Number Size | 18 | 2316.98 | 2.07 | 0.005 |
| Language x Number Size | 6 | 21.02 | 12.54 | < 0.001 |
| Age x Language x Number Size | 18 | 2316.46 | 1.09 | 0.355 |
| Note: Number Size has 8 levels: ‘30s, ‘40s, ‘50s, ‘60s, ‘70s, ‘80s, ‘90s. | | | | |

The model converged and the interaction between Language and Number Size was significant (F (6,21.02) = 12.54, p < .001).

### “Stepwise” contrasts:

The following custom contrasts compare each decade with the previous and following one. For example ‘30 vs ‘40, ‘40 vs ‘50, etc.

| French | | | | |
| --- | --- | --- | --- | --- |
| contrasts | estimate | SE | z.ratio | p.value |
| 30 - 40 | 46.62 | 50 | 0.93 | 0.35 |
| 40 - 50 | -52.09 | 50 | -1.04 | 0.30 |
| 50 - 60 | -75.68 | 49.8 | -1.52 | 0.13 |
| 60 - 70 | -194.7 | 51.1 | -3.81 | 0.0001 |
| 70 - 80 | -3.28 | 51.9 | -0.06 | 0.95 |
| 80 - 90 | 99.97 | 50.9 | 1.96 | 0.05 |
| German | | | | |
| contrast | estimate | SE | z.ratio | p.value |
| 30 - 40 | 35.01 | 44.3 | 0.79 | 0.43 |
| 40 - 50 | 51.23 | 44.4 | 1.15 | 0.25 |
| 50 - 60 | -18.19 | 44.5 | -0.41 | 0.68 |
| 60 - 70 | -38.15 | 44.5 | -0.86 | 0.39 |
| 70 - 80 | 38.73 | 44.3 | 0.87 | 0.38 |
| 80 - 90 | -60.04 | 44.2 | -1.36 | 0.17 |

In French, ‘30s, ‘40s, ‘50s, ‘60s numbers have comparable RTs. However, ‘60s have significant faster RT than ‘70s, which are not different from ‘80s. Interestingly, ‘90s are (p = .05) almost slower than ‘80s. In German there are no differences among the decades (all p > .17).

Figure 1 Mean reaction time for each decade at each age groups. Ribbons represent one standard error.

## Verbal-visual matching

|  | num Df | den Df | F | Pr(>F) |
| --- | --- | --- | --- | --- |
| Age | 3 | 94.85 | 55.64 | <0.001 |
| Language | 1 | 20.95 | 282.15 | <0.001 |
| Number Size | 6 | 20.87 | 5.51 | 0.001 |
| Age x Language | 3 | 2317.05 | 67.15 | <0.001 |
| Age x Number Size | 18 | 2315.26 | 2.75 | <0.001 |
| Language x Number Size | 6 | 20.74 | 9.10 | <0.001 |
| Age x Language x Number Size | 18 | 2314.64 | 1.59 | 0.054 |

The model converged and the interaction between Language and Number Size was significant (F (6,20.74) = 9.10, p < .001).

Figure 2: Mean reaction time for each decade at each age groups. Ribbons represent one standard error.

### Stepwise” contrasts:

The following custom contrasts compare each decade with the previous and following one. For example ‘30 vs ‘40, ‘40 vs ‘50, etc.

| French | | | | |
| --- | --- | --- | --- | --- |
| contras | estimate | SE | z.ratio | p.value |
| 30 - 40 | -154.04 | 84.6 | -1.82 | 0.07 |
| 40 - 50 | 98.16 | 84.8 | 1.16 | 0.24 |
| 50 - 60 | -127.58 | 85.6 | -1.49 | 0.13 |
| 60 - 70 | -222.20 | 86.6 | -2.56 | 0.01 |
| 70 - 80 | -105.11 | 87.4 | -1.20 | 0.23 |
| 80 - 90 | 43.99 | 88.5 | 0.50 | 0.62 |
| German: | | | | |
| contras | estimate | SE | z.ratio | p.value |
| 30 - 40 | -16.31 | 77.8 | -0.21 | 0.83 |
| 40 - 50 | 14.37 | 77.7 | 0.18 | 0.85 |
| 50 - 60 | -43.83 | 77.9 | -0.56 | 0.57 |
| 60 - 70 | 61.03 | 77.9 | 0.78 | 0.43 |
| 70 - 80 | -99.86 | 77.7 | -1.28 | 0.20 |
| 80 - 90 | 0.391 | 77.8 | 0.005 | 0.99 |

In French, ‘30s, ‘40s, ‘50s, ‘60s numbers have comparable RTs. However, ‘60s have significant faster RT than ‘70s, which are not different from ‘80s. In German there are no differences among the decades (all p > .20).

# References:

Avanzi, M., & Thibault, A. (2019). Documenter le système vigésimal et sa contrepartie décimale (70-80-90) en français et dans les dialectes galloromans. *Langages*, *N°215*(3), 89. https://doi.org/10.3917/lang.215.0089

Bahnmueller, J., Moeller, K., Mann, A., & Nuerk, H.-C. (2015). On the limits of language influences on numerical cognition – no inversion effects in three-digit number magnitude processing in adults. *Frontiers in Psychology*, *6*. https://doi.org/10.3389/fpsyg.2015.01216

Bahnmueller, J., Nuerk, H.-C., & Moeller, K. (2018). A Taxonomy Proposal for Types of Interactions of Language and Place-Value Processing in Multi-Digit Numbers. *Frontiers in Psychology*, *9*. https://doi.org/10.3389/fpsyg.2018.01024

Barrouillet, P., Camos, V., Perruchet, P., & Seron, X. (2004). ADAPT: A Developmental, Asemantic, and Procedural Model for Transcoding From Verbal to Arabic Numerals. *Psychological Review*, *111*(2), 368–394. https://doi.org/10.1037/0033-295X.111.2.368

Bernardo, A. B. I. (2001). Asymmetric activation of number codes in bilinguals: Further evidence for the encoding complex model of number processing. *Memory & Cognition*, *29*(7), 968–976. https://doi.org/10.3758/BF03195759

Camos, V. (2008). Low working memory capacity impedes both efficiency and learning of number transcoding in children. *Journal of Experimental Child Psychology*, *99*(1), 37–57. https://doi.org/10.1016/j.jecp.2007.06.006

Campbell, J. (1995). Mechanisms of Simple Addition and Multiplication: A Modified Network-interference Theory and Simulation. *Mathematical Cognition*, *1*, 121–164.

Chrisomalis, S. (2010). *Numerical Notation: A Comparative History*. Cambridge University Press.

Clayton, F. J., Copper, C., Steiner, A. F., Banfi, C., Finke, S., Landerl, K., & Göbel, S. M. (2020). Two-digit number writing and arithmetic in Year 1 children: Does number word inversion matter? *Cognitive Development*, *56*, 100967. https://doi.org/10.1016/j.cogdev.2020.100967

De Visscher, A., & Noël, M.-P. (2014). The detrimental effect of interference in multiplication facts storing: Typical development and individual differences. *Journal of Experimental Psychology: General*, *143*(6), 2380–2400. https://doi.org/10.1037/xge0000029

Dehaene, S. (1992). Varieties of numerical abilities. *Cognition*, *44*(1), 1–42. https://doi.org/10.1016/0010-0277(92)90049-N

Dehaene, S., Piazza, M., Pinel, P., & Cohen, L. (2003). Three parietal circuits for number processing. *Cognitive Neuropsychology*, *20*(3–6), 487–506. https://doi.org/10.1080/02643290244000239

Deloche, G., & Seron, X. (1982). From three to 3: A differential analysis of skills in transcoding quantities between patients with Broca’s and Wernicke’s aphasia. *Brain*, *105*(4), 719–733. https://doi.org/10.1093/brain/105.4.719

Dijkstra, T., & van Heuven, W. J. B. (2002). The architecture of the bilingual word recognition system: From identification to decision. *Bilingualism: Language and Cognition*, *5*(3), 175–197. https://doi.org/10.1017/S1366728902003012

Dotan, D., & Friedmann, N. (2018). A cognitive model for multidigit number reading: Inferences from individuals with selective impairments. *Cortex*, *101*, 249–281. https://doi.org/10.1016/j.cortex.2017.10.025

Dowker, A., & Nuerk, H.-C. (2016). Editorial: Linguistic Influences on Mathematics. *Frontiers in Psychology*, *7*. https://doi.org/10.3389/fpsyg.2016.01035

Ellis, N. C., & Hennelly, R. A. (1980). A bilingual word-length effect: Implications for intelligence testing and the relative ease of mental calculation in Welsh and English. *British Journal of Psychology*, *71*(1), 43–51. https://doi.org/10.1111/j.2044-8295.1980.tb02728.x

Friso-van den Bos, I., van der Ven, S. H. G., Kroesbergen, E. H., & van Luit, J. E. H. (2013). Working memory and mathematics in primary school children: A meta-analysis. *Educational Research Review*, *10*, 29–44. https://doi.org/10.1016/j.edurev.2013.05.003

Gathercole, S. E., & Baddeley, A. D. (1993). Phonological working memory: A critical building block for reading development and vocabulary acquisition? *European Journal of Psychology of Education*, *8*(3), 259. https://doi.org/10.1007/BF03174081

Geary, D. C., Bow‐Thomas, C. C., Liu, F., & Siegler, R. S. (1996). Development of Arithmetical Competencies in Chinese and American Children: Influence of Age, Language, and Schooling. *Child Development*, *67*(5), 2022–2044. https://doi.org/10.1111/j.1467-8624.1996.tb01841.x

Göbel, S. M., Moeller, K., Pixner, S., Kaufmann, L., & Nuerk, H.-C. (2014). Language affects symbolic arithmetic in children: The case of number word inversion. *Journal of Experimental Child Psychology*, *119*, 17–25. https://doi.org/10.1016/j.jecp.2013.10.001

Gollan, T. H., Montoya, R. I., Fennema-Notestine, C., & Morris, S. K. (2005). Bilingualism affects picture naming but not picture classification. *Memory & Cognition*, *33*(7), 1220–1234. https://doi.org/10.3758/BF03193224

Grosjean, F. (2010). *Bilingual: Life and reality*. Harvard University Press.

Haspelmath, M., Dryer, M. S., Gil, D., & Comrie, B. (2005). *The World Atlas of Language Structures*. Oxford Univ. Press.

Ifrah, G., & Bellos, D. (2000). *The universal history of numbers: From prehistory to the invention of the computer*. Wiley.

Imbo, I., Vanden Bulcke, C., De Brauwer, J., & Fias, W. (2014). Sixty-four or four-and-sixty? The influence of language and working memory on children’s number transcoding. *Frontiers in Psychology*, *5*. https://doi.org/10.3389/fpsyg.2014.00313

Ivanova, I., & Costa, A. (2008). Does bilingualism hamper lexical access in speech production? *Acta Psychologica*, *127*(2), 277–288. https://doi.org/10.1016/j.actpsy.2007.06.003

Krinzinger, H., Gregoire, J., Desoete, A., Kaufmann, L., Nuerk, H.-C., & Willmes, K. (2011). Differential Language Effects on Numerical Skills in Second Grade. *Journal of Cross-Cultural Psychology*, *42*(4), 614–629. https://doi.org/10.1177/0022022111406252

Lê, M.-L. T., & Noël, M.-P. (2020). Transparent number-naming system gives only limited advantage for preschooler’s numerical development: Comparisons of Vietnamese and French-speaking children. *PLOS ONE*, *15*(12), e0243472. https://doi.org/10.1371/journal.pone.0243472

Lonnemann, J., & Yan, S. (2015). Does number word inversion affect arithmetic processes in adults? *Trends in Neuroscience and Education*, *4*(1), 1–5. https://doi.org/10.1016/j.tine.2015.01.002

McCloskey, M. (1992). Cognitive mechanisms in numerical processing: Evidence from acquired dyscalculia. *Cognition*, *44*(1–2), 107–157. https://doi.org/10.1016/0010-0277(92)90052-J

McCloskey, M., Caramazza, A., & Basili, A. (1985). Cognitive mechanisms in number processing and calculation: Evidence from dyscalculia. *Brain and Cognition*, *4*(2), 171–196. https://doi.org/10.1016/0278-2626(85)90069-7

McClung, N. A., & Arya, D. J. (2018). Individual Differences in Fourth-Grade Math Achievement in Chinese and English. *Frontiers in Education*, *3*, 29. https://doi.org/10.3389/feduc.2018.00029

Meeuwissen, M., Roelofs, A., & Levelt, W. J. M. (2003). Planning levels in naming and reading complex numerals. *Memory & Cognition*, *31*(8), 1238–1248. https://doi.org/10.3758/BF03195807

Miller, K. F., Smith, C. M., Zhu, J., & Zhang, H. (1995). Preschool Origins of Cross-National Differences in Mathematical Competence: The Role of Number-Naming Systems. *Psychological Science*, *6*(1), 56–60.

Miller, K. F., & Stigler, J. W. (1987). Counting in Chinese: Cultural variation in a basic cognitive skill. *Cognitive Development*, *2*(3), 279–305. https://doi.org/10.1016/S0885-2014(87)90091-8

Miura, I. T., Kim, C. C., Chang, C.-M., & Okamoto, Y. (1988). Effects of Language Characteristics on Children’s Cognitive Representation of Number: Cross-National Comparisons. *Child Development*, *59*(6), 1445. https://doi.org/10.2307/1130659

Moeller, K., Zuber, J., Olsen, N., Nuerk, H.-C., & Willmes, K. (2015). Intransparent German number words complicate transcoding – a translingual comparison with Japanese. *Frontiers in Psychology*, *6*. https://doi.org/10.3389/fpsyg.2015.00740

Nuerk, H., Weger, U., & Willmes, K. (2005). Language effects in magnitude comparison: Small, but not irrelevant. *Brain and Language*, *92*(3), 262–277. https://doi.org/10.1016/j.bandl.2004.06.107

Nuerk, H.-C., Weger, U., & Willmes, K. (2004). On the Perceptual Generality of the Unit-Decade Compatibility Effect. *Experimental Psychology*, *51*(1), 72–79. https://doi.org/10.1027/1618-3169.51.1.72

Pixner, S., Zuber, J., Heřmanová, V., Kaufmann, L., Nuerk, H.-C., & Moeller, K. (2011). One language, two number-word systems and many problems: Numerical cognition in the Czech language. *Research in Developmental Disabilities*, *32*(6), 2683–2689. https://doi.org/10.1016/j.ridd.2011.06.004

Poncin, A., Van Rinsveld, A., & Schiltz, C. (2019). Units-first or tens-first: Does language matter when processing visually presented two-digit numbers? *Quarterly Journal of Experimental Psychology*, *73*(5), 726–738. https://doi.org/10.1177/1747021819892165

Power, R. J. D., & Dal Martello, M. F. (1990). The dictation of Italian numerals. *Language and Cognitive Processes*, *5*(3), 237–254. https://doi.org/10.1080/01690969008402106

Rodic, M., Zhou, X., Tikhomirova, T., Wei, W., Malykh, S., Ismatulina, V., Sabirova, E., Davidova, Y., Tosto, M. G., Lemelin, J.-P., & Kovas, Y. (2015). Cross-cultural investigation into cognitive underpinnings of individual differences in early arithmetic. *Developmental Science*, *18*(1), 165–174. https://doi.org/10.1111/desc.12204

Salillas, E., & Martínez, A. (2018). Linguistic Traces in Core Numerical Knowledge: An Approach From Bilingualism. In *Language and Culture in Mathematical Cognition* (pp. 173–196). Elsevier. https://doi.org/10.1016/B978-0-12-812574-8.00008-0

Salillas, E., & Wicha, N. Y. Y. (2012). Early Learning Shapes the Memory Networks for Arithmetic: Evidence From Brain Potentials in Bilinguals. *Psychological Science*, *23*(7), 745–755. https://doi.org/10.1177/0956797612446347

Seron, X., & Fayol, M. (1994). Number transcoding in children: A functional analysis. *British Journal of Developmental Psychology*, *12*(3), 281–300. https://doi.org/10.1111/j.2044-835X.1994.tb00635.x

Steiner, A. F., Banfi, C., Finke, S., Kemény, F., Clayton, F. J., Göbel, S. M., & Landerl, K. (2021). Twenty-four or four-and-twenty: Language modulates cross-modal matching for multidigit numbers in children and adults. *Journal of Experimental Child Psychology*, *202*, 104970. https://doi.org/10.1016/j.jecp.2020.104970

Sullivan, M. D., Poarch, G. J., & Bialystok, E. (2018). Why is Lexical Retrieval Slower for Bilinguals? Evidence from Picture Naming. *Bilingualism (Cambridge, England)*, *21*(3), 479–488. https://doi.org/10.1017/S1366728917000694

Van Rinsveld, A., Brunner, M., Landerl, K., Schiltz, C., & Ugen, S. (2015). The relation between language and arithmetic in bilinguals: Insights from different stages of language acquisition. *Frontiers in Psychology*, *6*. https://doi.org/10.3389/fpsyg.2015.00265

Van Rinsveld, A., Dricot, L., Guillaume, M., Rossion, B., & Schiltz, C. (2017). Mental arithmetic in the bilingual brain: Language matters. *Neuropsychologia*, *101*, 17–29. https://doi.org/10.1016/j.neuropsychologia.2017.05.009

Van Rinsveld, A., & Schiltz, C. (2016). Sixty-twelve = Seventy-two? A cross-linguistic comparison of children’s number transcoding. *British Journal of Developmental Psychology*, *34*(3), 461–468. https://doi.org/10.1111/bjdp.12151

Vander Beken, H., & Brysbaert, M. (2018). Studying texts in a second language: The importance of test type. *Bilingualism: Language and Cognition*, *21*(5), 1062–1074. https://doi.org/10.1017/S1366728917000189

Volmer, E., Grabner, R. H., & Saalbach, H. (2018). Language switching costs in bilingual mathematics learning: Transfer effects and individual differences. *Zeitschrift Für Erziehungswissenschaft*, *21*(1), 71–96. https://doi.org/10.1007/s11618-017-0795-6

Wicha, N. Y., Dickson, D. S., & Martinez-Lincoln, A. (2018). Arithmetic in the Bilingual Brain. In *Language and Culture in Mathematical Cognition* (pp. 145–172). Elsevier. https://doi.org/10.1016/B978-0-12-812574-8.00007-9

Xenidou-Dervou, I., Gilmore, C., van der Schoot, M., & van Lieshout, E. C. D. M. (2015). The developmental onset of symbolic approximation: Beyond nonsymbolic representations, the language of numbers matters. *Frontiers in Psychology*, *6*. https://doi.org/10.3389/fpsyg.2015.00487

Zuber, J., Pixner, S., Moeller, K., & Nuerk, H.-C. (2009). On the language specificity of basic number processing: Transcoding in a language with inversion and its relation to working memory capacity. *Journal of Experimental Child Psychology*, *102*(1), 60–77. https://doi.org/10.1016/j.jecp.2008.04.003

1. In these analyses, we ran the same Linear Mixed Models, with a factor including all the levels of the decades (*i.e.* ‘30s to ‘90s). This allowed contrasting each decade with respectively previous and following one, to spot a “statistical jump” in terms of RT. The jump in French occurred prevalently between ‘60s and ‘70s (the latest being significantly slower). Indicating that ’60s are indeed not slower to process than ’50s and justifying the drop out of these decades from the comparisons.
