# Supplementary material for: Number transcoding in bilinguals—A transversal developmental study
Source: PLoS One. 2022 Aug 29;17(8):e0273391. doi: 10.1371/journal.pone.0273391 (PMC9423630; doi:10.1371/journal.pone.0273391)
Supplement: Attachment — Submitted filename: Response to Reviewers_Revision2_Final.docx [file pone.0273391.s006.docx]

Dear Editor, Prof. Dr Prado,

dear Reviewer,

We would like to thank the editor and the two anonymous reviewers for their very valuable comments and their constructive suggestions on this second revision.

We identified two major points which needed further details or adjustements: (1) the introduction of the cognitive number transcoding models, and (2) the description of the results.

In this second revision of the manuscript we have addressed these concerns in depth and believe the manuscript’s quality has yet again significantly improved as a consequence. In the following, we describe how we implemented the different points raised by both reviewers into the revised manuscript.

Below you can find our answers (A) to the comments (C) to each of the two reviewer’s points. Please note that adjustments in the manuscript are indicated in this colour code.

# Reviewer #1:

C 1. *The authors added a discussion of theoretical models, following my request. This is big improvement. In the discussion section, the theoretical relation with the model is good in my opinion (see only minor comments below). In contrast, the presentation of models in the introduction still feels a bit out-of-context. I didn’t expect a general summary of theoretical models of number transcoding, but a more concrete explanation of how these issues are related with the present study.*

**A1.** We thank the reviewer for this comment. According to the suggestion that the models felt out of context, we have added several sentences to link the different models with the topics of transcoding, multilingualism, and second language acquisition. We have also specified our hypothesis and linked it to the ADAPT model in a later paragraph. However, please note that the study was not designed to specifically test those models, hence most of the matches between our study and the models' predictions are made *a posteriori*, (l. 51 to 79). Note that we have removed the description of the different brain parts of the TCM to avoid a lengthy paragraph.

Several cognitive models have been proposed to describe transcoding. While some are taking also into account cognitive development, most of these models do not specifically account for transcoding differences between languages and multilingualism. These models are summarized below in two main categories: semantic and asemantic (Barrouillet et al., 2004).

In semantic models, transcoding requires an obligatory access of the number’s magnitude. For example McCloskey (McCloskey, 1992; McCloskey et al., 1985) proposed a transcoding model in which the entry number - regardless from the input’s format - accesses an abstract semantic representation. Power and Dal Martello (Power & Dal Martello, 1990) further proposed a model specifically for number dictation (*i.e.* verbal to Arabic format) which differs from the previous one in that semantic representations reflect the verbal word structure of the numbers, thus predicting differences between languages. Semantic models hence assume that transcoding difficulties depend on the quality and maturity of the semantic representations, therefore predicting worse performances in children, in particular for larger numbers.

Asemantic models propose that numbers can be transcoded without accessing magnitude. Deloche and Seron (Deloche & Seron, 1982) proposed such a model for number naming (*i.e.* Arabic to verbal) where the first step involves parsing the input into primitives which are then submitted to a set of rules for the output system. Later on, Barrouillet and colleagues (Barrouillet et al., 2004) proposed ADAPT (A Developmental Asemantic Procedural Model), explicitly including a developmental perspective on transcoding. In ADAPT, number words become lexicalized with training, hence directly retrieved from long-term memory and bypassing the procedural processes. Therefore transcoding in children would depend on language-dependent characteristics. Yet, the language-dependent characteristics should diminish with increasing lexicalization. Another more general model considering number representations is the Triple Code Model (TCM). The TCM proposes a functional and topographical framework of how and where in the brain approximate, visual symbolic and verbal codes are processed. ~~Approximate processing of magnitudes (~~*~~i.e.~~* ~~not exact quantities) are taken over by the horizontal segment of the intraparietal sulcus. Visual symbols, depending on visuospatial abilities, are processed by the parietal and fusiform gyrus (number form area). Finally, verbal codes, depending on linguistic processes, involve the left angular gyrus of the temporal-parietal gyrus~~ (Dehaene et al., 2003)~~.~~ The TCM also implies asemantic language-dependent transcoding from or to a verbal code (Dehaene, 1992). Language-specific training in transcoding would therefore increase the strength of the association between each code and their respective brain areas. More recently, Dotan and Friedmann (Dotan & Friedmann, 2018) proposed a model for Arabic to verbal transcoding where numbers are first visually analysed for identity, order and decimal structure to build a language independent number word frame, which is passed to a second system which applies language-specific rules which are associated with their phonological and articulatory counter-parts. In sum, asemantic models predict that verbal transcoding depends on language characteristics. However, those models do not explicitly model multilingualism, nor how transcoding develops by acquiring number words in a second language.

See also lines (307-318) about the hypothesis, which we linked to the above-mentioned predictions, herein we also integrated C6 concerning more precise hypotheses:

For coherence between the models and the hypothesis, we also added the following sentence in the discussion about transparency of power (lines 513-516):

However, this interpretation can be excluded, because the same comparison in German did not reveal any differences. Assuming that 5^th^ graders do not yet have mature magnitude representations this result also speaks against the predictions of semantic models.

C 2. *Lines 104-105: why are 70’s a particular case?*

**A 2.** We thank the reviewer for this comment. The remarks on teens in 70’s French number words were added as an answer to a comment from reviewer 2 (see reviewer 2 first revision, C2.1 and A 2.1: “*I’d say that the true base 20 starts at 80, as the 60’s follow the regular base 10 system and the 70’s are neither (as they are 60+10, 60+11 etc). And then there are the irregular number words for 11-16.”*). However, after further reflections on the morpho-syntactic structure of French number words, it becomes clear to us that the considerations on teens also apply to ‘90s number words, which might have led to confusion. Yet, as described in the annexed analyses provided in the previous revision comparing each decade, we did not find differences between ’80s (without teens) and ‘70s or ‘90s with teens (lines 106-110)

However, `80s to `90s follow a base-20 system, *e.g.* 87 = *4*20 + 7,* “*quatre-vingt*-sept”, literally “*four-twenty*-seven”. Moreover, the teens (11 to 16) are lexical primitives, like in English and in stark contrast to the more transparent Asian languages. Note that the transparency contrast is additionally increased for 71 to 76 and 91 to 96, which are composed with a base-20 and teens, *e.g.* 96 = 4*20 + *16*, “quatre-vingt-*seize*”, “four-twenty-*sixteen*”.

C 3. *Line 107: I did not understand the Belgian Wallonia dialect, perhaps elaborate. The 80’s are same as in France-French, but what’s the difference? Do they say septante and nonante?*

**A.3.** Yes in Belgian Wallonia, septante and nonante are commonly used (see Seron & Fayol, 1994). We adjusted the sentence to improve the clarity of that description of the french counting system and added emphasis with *italic* about the corresponding english translation (lines 110 to 113). (See also R2 C1).

Furthermore, the vigesimal system is subject to regional variances, for example in Belgian Wallonia `70s and ‘90s are not vigesimal (i.e. “*sept*ante” for “*seven*ty” and “*non*ante” for “*nine*ty”, see (Seron & Fayol, 1994)) and in some Swiss-French cantons the vigesimal system is entirely absent (*i.e.* including for 80, “*huit*ante”*,* literally “*eigh*ty”).

C 4. *Lines 268-269, line 341: was z-scoring done separately for each age group?*

**A 4.** We thank the reviewer for noticing this important point, yes it was done separately for each age group, we added the following sentence (line 278-279):

To control for age related variabilities analogous analyses were conducted on *z-score* transformed RT, separately for each group.

C 5. *Fig. 1 seems to me unnecessary. It could be easily explained in the text. For your consideration.*

**A 5.** We added Fig.1 after noticing a problem on our side to describe the analyses design such that it is easily understood and we think the figure might add clarity about the design to the reader. We agree this could be a repetition of information, but the design rationale being fundamental to understanding the interpretation of the results, we decided to keep Fig. 1.

C 6. *Lines 296-301: you wrote precisely which conditions will be compared, this is excellent, but please write also the specific predictions. Don’t leave it to the reader to guess.*

**A.6.** We agree that adding the predictions with the analysis designs would improve the reader’s understanding. We modified the paragraph adding more precise precise predictions (see lines 307-318):

The analyses aimed at testing for all age groups the two main hypotheses: (1) the effect of *transparency of power* in French and (2) the effect of *language of math acquisition*. First, the effect of *transparency of power* in French, corresponding to the cost for vigesimal numbers in the base-20 system was tested by comparing small numbers (*i.e.* ’30s, ’40s, and ’50s) to large numbers (*i.e.* ’70s, ’80s, and ’90s) in French only (see A in Fig 1). For this hypothesis, we predicted that both tasks would lead to worse performances for large compared to small numbers, henceforth a cost. Developmentally, the transparency cost might either be re-absorbed with increasing training using LM2 number words or remain stable across age groups. Additional control for the potential confounder of a number size effect was implemented by applying the same comparison within German (A.1 in Fig 1). Second, the hypothesis concerning the *language of math acquisition* was tested by comparing only small numbers (*i.e.* ’30s, ’40s, and ’50s) in French and German (B in Fig 1). We predicted that both tasks would lead to a cost for French compared to German. Developmentally, this cost could remain stable throughout adolescence and adulthood, or gradually diminish with age, potentially even resorbing in adults.

C 7. *The model description in lines 304-310 is confusing. You talk about what you did and what you intended to, going back and forth between the two. Perhaps just write what your model actually was. Then, if you want, you can tell about additional things you tried but failed (personally I feel this is neither needed nor common, but it’s up to you). This should be an extremely simple paragraph to read, but I found myself having to re-read it. Similar issues in lines 346-351.*

**A.7.** We thank the reviewer for this comment about clarity which is reflected also by the second reviewer comments (see R2 C12). To facilitate understanding we have completely written anew the model’s description, as follows (lines 321 -332):

The linear mixed model was defined with main effects and interactions between the fixed between-group factor *Age* (levels: 5^th^, 8^th^, 11^th^ grade and adults) and two fixed within-group factors: *Language* (German, French), and *Number Size* (Large, Small), all levels being defined as categorical. As random factors, we modelled individual differences (*i.e. Subject*) and item-related variability (*i.e. Item*), the latter being considered separately by language. Individual differences were modelled using random intercepts per subject. Item-related variability was modelled using both random intercepts and slopes as a function of languages. In sum, the model takes the following in R syntax (A):

(A) $RT \sim1 + Age*Language*Number Size + (1|Subject) + (1 + Language|Item)$*.*

P-values and degrees of freedom were obtained with the Satterthwaite approximation method by the comparison of the full model against the model without the effect (Singmann et al., 2020). Follow-ups were calculated by comparing estimated marginal means (EMM) obtained with the emmeans package (Lenth, 2021), and p-values were adjusted with the Bonferroni method.

We have changed also the model description of correct responses (lines 375-380):

Correct responses (CR) were analysed using a binomial approach and *p-values* estimated by the likelihood ratio test. Since applying the same model as for RT (see (A)) did not converge, we had to drop the random per-item factor and the fixed factor *Age*. In sum the model had the following syntax (B):

(B) CR ~ Language*Size + (1|Sujet)

The failure of convergence with the more complex model might be due to ceiling effects, as the task was very simple, particularly for older age groups, see Fig 4 and S1 Table 2.

C 8. *The statistics is not sufficiently ordered / detailed. I will not go through each analysis, because there are many of them. I only provide examples here, but please fix these issues in all analyses, not just the examples I give here:*
     a. *Define the LMM variables fully and explicitly. E.g. was age a categorical or numerical factor? If categorical, specify the levels. Were Subject and Item defined as random factors (they should have been)? Which variables were within/between subjects?*

**A8.a.** We added the details of the levels, cfr the paragraph above (lines 321 -332).

b. *Write statistical results either in a table or in the text, no need for both. For example lines 312-319 vs. Table 1. Lines 353-356 can be simply deleted.*

**A8.b.** We thank the reviewer for this suggestion, in agreement with this suggestion, we removed the within text results to keep them exclusively in the tables. Consequently, we have adjusted some formulations. Note that, according to reviewer 2 suggestion (R2 C11), we have however added the descriptive statistics of the effects. For example (lines 334-342, but see also 382-390, 410-416 and 458-431).

The model for the reading aloud task RTs resulted in three significant main effects and three two-way interactions (see Table 1). The main effect of Age was decomposed with follow-up analyses showing that the 5^th^ graders (1091*(290)* ms) were slower than the older groups, which had similar RT’s (8^th^ 797*(288)* ms, 11^th^ 780*(247)* ms and adults 828*(320) ms*; standard deviations in parenthesis). Furthermore, the main effect of Language indicated overall slower naming in French than in German, and the main effect of Number Size indicated slower naming for bigger than smaller numbers. Significant two-way interactions were also found between Age and Language, as well as Age and Number Size. Finally, critically for the hypothesis, the two-way interaction between Language and Number Size was significant, while the triple interaction was not significant, see Table 1 and Fig 2.

c. *When talking about follow-up analyses, write what the statistical test was and its result. E.g. line 314, lines 326-327, and more (which statistical test does the z stand for? You use it a lot, perhaps you wrote somewhere in methods but I can’t remember all the details).*

**A8.c.** We have fixed this, the follow-up analyses were done by pairwise comparisons of the estimated marginal means of the models. The z stand for Walden z test which could be calculated since the degrees of freedom was defined as asymptotic. As underlined by the reviewer we have changed the parameters to calculate Satterthwaite corrected degrees of freedom (and added Bonferroni corrected p-values) to enable pairwise t-test comparisons with which the reader would be more familiar. Note that those changes did not modify the follow-up significancy patterns (lines 348-350, but also 300-302):

Follow-up analyses were performed on the model's estimated marginal means (EMMs) of the interaction's terms (see Singmann, 2021) with Satterthwaite estimation of degrees of freedom. Then EMM were compared with two-tailed pairwise tests with Bonferroni adjusted p-values.

See also changes at lines 422-423:

Follow-up analyses were applied comparing the estimated marginal means with paired comparisons on Satterthwaite corrected degrees of freedom and Bonferroni adjusted p-values

d. *When the variable had 2 possible values, or for 2 x 2 interactions, describe the direction of the results in the text (e.g. lines 312-319, effects of Number Size and Size-Language interaction, and the info is missing in several other places too).*

**A8.d.** We thank the reviewer for this comment. We have added the direction of the Number Size and the Language The Number-Size and Language interaction is described in the follow-up for the reading task. For the matching task, we do not describe the directions of the two-way interactions since there is a three-way interaction (and also given the analyses design in Fig. 1.), which in turn is described in the follow-up in the matching task. (see lines 337-342).

Furthermore, the main effect of Language indicated overall slower naming in French than in German, and the main effect of Number Size indicated slower naming for bigger than smaller numbers. Significant two-way interactions were also found between Age and Language and Age and Number Size. Finally, critically for the hypothesis, the two-way interaction between Language and Number Size was significant, while the three-way interaction was not significant, see Table 1 and Fig 2.

e. *For each analysis, I’d recommend presenting the interesting effect first, and the unimportant/trivial/boring effects later. You sometimes start with the boring one, and this is… well… a bit boring.*

**A8.e.** We thank the reviewer for the suggestion to change the order of presentation of the effects. The order might be a little boring to read, but on the other side, it is a standard order (main effects, two-way interactions, three-way, ect) which is now even more important to respect since the reader will need to rely on the table to find the respective statistical result (see C8.b.). With the current changes (i.e. only having the results in the tables) we hope the eventually attentive and hurried reader might jump directly jump to the follow-up or sum paragraphs, whose logic is explained visually in Fig.1.

f. *Linear mixed models: how was the p-value computed? There are number of ways to do this in LMM, explain which one was used.*

**A.8.f.** We thank the reviewer for this remark, we specified how the p-values were computed (computing an ANOVA with the complete model compared to the model without the effect in question). (lines 329 -330).

P-values and degrees of freedom were obtained with the Satterthwaite approximation method by the comparison of the full model against the model without the effect.

g. *Perhaps also explain Table 1, 3rd column. Degrees of freedom are typically integer. I know that in some cases they may be floating point, but it may be a good idea to say something about why this happened or what they mean*

**A.8.g.** We thank the reviewer for the comment. The degrees of freedom are calculated with Satterthwaite approximation (which relies on parametric bootstrapping, see (Luke, 2017)). Please see the answer above for the corresponding line (A.8.f.).

h. *Use standard terminology to describe the LMM. E.g., say random factor (with or without random slopes) instead of other terminology, e.g. in lines 375-376, which I admit I did not understand.*

**A.8.h.** We thank the reviewer for this comment. We have adapted the terminology accordingly (l. 325-326).

Individual differences were modelled using random intercepts per subject. Item-related variability was modelled using both random intercepts and slopes as a function of languages

i. *When relevant (e.g. in follow-up analyses), specify whether the p values are one-tailed or two-tailed.*

**A.8.i.** We thank the reviewer for this comment**.** All p-values are two-tailed. We added the information in the revision as following (l. 349-350):

Then EMM were compared with pairwise tests on the relevant interactions, two-tailed p-value are Bonferroni adjusted.

C 9. *Line 343-344: interesting and important point. But please explain why this is important.*

**A.9.** We thank the reviewer for this comment, according to the suggestion, we have added the explanation, as follows (lines 369-372):

The interaction subsisted also in further analyses limiting the dataset to number words with 4 syllables (see S2 Table 3) to control for the possible world length effect confounder (see (see Ellis & Hennelly, 1980)). Critically none of the analyses resulted in three-way interactions between Age, Language and Number Size.

C 10. *Line 302: I assume that when analyzing reaction times, you removed the incorrect-response trials? This should be the case, as incorrect trials cannot be expected to exhibit the same RTs. Same issue in the matching task*

**A.10.** We thank the reviewer for this comment, indeed the RT analyses are done on the correct responses exclusively. This is described in the data filtering part, at the beginning of the results (lines 294-296).

Finally, for reaction time analyses all incorrect responses were removed, additionally removing 2.93 % of the reading task and 3 % of the matching task, corresponding to the total error rate.

C 11. *Lines 397-403 – the statistics is not written clearly, and I suspect it’s also not optimal. You write that the hypothesis on language of acquisition was not supported, but I was not convinced this is correct. I suspect that if you analyze all age groups together, of course limiting to numbers < 60, you will get a significant effect.*

**A 11.** We thank the reviewer for this comment which overlaps with reviewer 2 (C.12). Indeed despite the fact that the difference between German and French seems visible on the verbal matching task’s RT figures, it was not significant in the follow-up analyses (we even tried with less conservative p-value adjustments, but we obtain the same pattern of significance, see R2 A.12). We tried some additional analyses excluding the 5^th^ graders from the dataset, since they have the biggest variance and difference. For the follow-up contrasts regarding the language of acquisition, we obtained almost the same pattern: near significant difference in 8^th^ graders (t(78.3) = 2.65, p = .06), significant for 11^th^ (t(96.8) = 3.1, p = .01) but not for adults (t(122.2) = 1.88, p = .37). Nevertheless, the z-score transformed data (which we originally decided to compare also because of this doubt) does not lead to an interaction with age.

C 12. *Certainly, I wouldn’t present it as if the effect existed only in 5th grade and nothing at all for older ages (line 439). You have very nice data, no reason to undermine it. This is also a more-general issue about follow-up analyses: you don’t have to run t-test (or whatever similar test it was) on small portions of the data. Why not just run LMM on the relevant subset of data?*

**A.12.** We thank the reviewer for this comment. Indeed we could have used a simple effect analysis as suggested. Here we followed the procedure suggested by (Singmann & Kellen, 2019), doing the follow-up with pairwise comparisons on the estimated marginal means. We have modified the summary to further clarify our findings (l. 473-477):

In summary, for the verbal-visual matching task the (1) *transparency of power* hypothesis could be confirmed, as transcoding performances were overall lower for French ’70s, ’80s and ’90s numbers having a vigesimal structure, stably across age groups. Concerning the hypothesis on (2) *language of math acquisition,* the advantage for transcoding in German (LM1) was also stable across age groups, when considering standardized RTs (see Fig. 6).

C 13. *The detailed analyses in the annex are interesting. I think it would be nice if mentioned them very briefly in the main text – perhaps just say the main conclusions and reference the annex. When I read the previous manuscript version I was worried about these issues, other readers may feel the same and you have good answers. Also, some of the things you wrote in the response to review, which were correct and important in my opinion, may be added to the annex (currently it’s stats and data, without much conclusions).
      E.g., lines 507-509 may be a relevant place to mention this.*

**A.13.** We thank the reviewer for this suggestionsand we added the supplementary analysis in the supplementary materials as suggested and refer to it in the text (at lines 546-549 and lines 661-663).

Also, in answer to an interesting comment in the review process, we conducted additional analyses on reaction times (see S4), by adding all decades as levels for the Number Size factor (hence 8 levels from ‘30s to ‘90s). Those additional analyses confirm that for both tasks in French, reaction times become slower from the ‘70s decade onwards.

C 14. *Line 492 claims that number matching is transcoding from verbal to visual. This is incorrect. You can’t know which transcoding direction the participants use in a number matching task.*

**A 14.** We thank the reviewer for this comment, we have corrected the sentence to adapt to the suggestion (line 530-531):

Notably, the cost was found for both reading aloud and number matching tasks that relies on transcoding from visual to verbal processes and vice-versa.

C 15. *Please write in supplementary material files which task they refer to.*

**A 15.** We thank the reviewer for this suggestion, we have added the headers in the supplementary materials.

C 16. *Line 493-494: the finding that the transparency of power effect on z-scored RTs remains stable across age is interesting. However, you did not say which data supports it. In my opinion, the critical analysis that supports it is the absence of triple interaction in the z-scored data. So if you want to reach this conclusion, you should mention the triple interaction issue in the results section (maybe even move this z-scored analyses into the main text).*

**A.16.** We thank the reviewer for this suggestion, we have added the following sentences in the corresponding results section (lines 444-446):

The linear model applied on z-scores (see Fig 6) replicated the results with raw data described above, except, the three-way interaction with age, suggesting that the age differences observed with raw RTs, might be caused by differences between age-group variability (see S2 Table 3)

C 17. Line 497-515: I think you propose an interesting idea – that the difficulty in 70-99 may arise either from syntactic complexity or from morphological-retrieval complexity of these numbers. However, I was not convinced by your claim that the former interpretation necessarily relates to ADAPT and the latter necessarily relates to Dotan & Friedmann. I think that both models can accommodate both interpretations.

**A.17.** We thank the reviewer for this comment. We might have not expressed ourself clearly enough, we did not thought to contrast the ADAPT to the Dotan & Friedmann model, but to discuss how our results can fit with them – in particular with ADAPT. We have reformulated the paragraph as follows (see lines 551-555):

Finally, the cost for `70s, `80s and `90s numbers also fits with Dotan and Freidman’s recent transcoding model which suggests that reading `70s and `90s French number words requires additional irregular rules. When reading 75 for example, a number word frame would be structured by a decade and a teen instead of ten, then the ten frame filling would be changed from “7” to “6” (the filled frame would be: [6:tens] [15:teens] to give “*soixante*-*quinze*”, literally “*six*ty*-fifteen*”) (Dotan & Friedmann, 2018).

C 18. *The idea of proactive interference (line 515) seems far-fetched. Retrieving 4 morphemes instead of 2 may be difficult for several different reasons, I am not sure I see a reason to point specifically at PI. In any case, Campbell’s and De Visscher’s studies on multiplication talk about a scenario quite different – not interference of single words because they are numerous, but of multi-word multiplication exercise because they are similar. The analogy seems to me more misleading than illuminating.*

**A.18.** We thank the reviewer for this comment, we aknowledge the position that the reviewer is not convinced by the proactive interference interpretation. Yet, we would like to mention this possibility in the discussion, as this reflection emerged after reading more general language-related literature, where words from the same lexical domain highly interfere with each other. If we consider number words as a well-defined and very frequent lexical domain, it could be that “morphologically composed numbers”, such as the vigesimal numbers, might cause interference. To clarify that this interpretation originates from the language literature we changed the following (lines 559-560)

Eventually, these morphemes, which are made of other numbers might interfere among each other and slow down RTs~~, in a similar fashion as for multiplication~~ (*i.e.* proactive interference (Campbell, 1995; De Visscher & Noël, 2014)).

C 19. *Line 543: presenting number reading as a form of free recall, or the matching task as a form of familiarity judgment, seems far-fetched and perhaps even misleading. I totally agree with you that the tasks have some resemblance, in the sense that the former two require unlimited retrieval, whereas the latter two require verification (lines 544-548). But you can say just that, no need to imply that the tasks are more identical than they really are.*

**A.19.** We thank the reviewer for this comment and respectfully disagree with the interpretation. Again, following the perspective of lexical retrieval, we argue that the underlying long-term memory cognitive mechanisms differ. For example considering the ADAPT model, which considers LTM retrieval for each morphemes, then both tasks might have recurred to a different form of retrieval (free recall vs recognition).

C 20. *Lines 576+: another limitation may be that it’s hard to disentangle language of math acquisition from an alternative interpretation that assumes intrinsic differences between German and French (“French is just harder”).*

**A.20.** We thank the reviewer for this comment, indeed both languages are intrinsically different. Concerning the difficulty – specifically for number words – there are mainly studies pointing at German number words to be harder to learn, compared, for example, to English number words. This is often explained by the inversion effect (see the introduction part in German and work from the Nuerk team in Tübingen for example). Therefore the interpretation that French is harder seems difficult to make since both languages have intrinsic charachteristics that would make it harder to learn: less *transparency of order* (German) and less *transparency of power* (French).

If what is meant by the reviewer is that it is harder to learn French *in general as a language* than German, then we also think both languages have their difficulties – for example compared to English. On one side French might be harder due to irregular verbs and less phono-grammatic transparency. But on the other hand German has multiple cases (Dativ, Genitiv and Akusativ) and a neutral “das” case which is inexistent in French. Also words are in average longer in German than in French. Finally, we are not aware of studies indicating if French is harder than German to learn.

# Reviewer #2:

*I read a revised version of the manuscript and first I applaud the authors for the endeavor to greatly rewrite the work including a complete change of the analyses. That is a large step that undoubtedly took quite some time but it is definitely a large improvement.
I noted that reviewer 1 is much more knowledgeable than me on the theoretical details. It looks like that improved a lot too but reviewer 1 is probably much better able than me to judge that (if also rereviewing the manuscript).
I do still have some remaining questions, mostly about the analyses.*

C 1. *p. 4-5 for the reader unfamiliar with those languages it would be helpful to give an example in words, not just in formula of 10*x+y (and also define x and y). You do this for French now but not for German (I’d also give the example both in the original language and its counterpart in English).*

**A.1.** We thank the reviewer for this comment, we agree it would be helpful for the reader. We have implemented the following changes (and an italic emphasis to find the corresponding unit/decade) (lines 93 -113). For the ulterior changes refer to (R1 C3).

*Transparency of power* refers to the existence of different degrees of morpho-syntactic language transparencies in verbal numbers. In many Asian languages (*i.e.* Mandarin Chinese, Vietnamese) the morpho-syntactic structure of the verbal number system is highly consistent with the Arabic number system, and in general with the base-10 (*i.e.* 10**x + y*, where x indexes the base index and y the unit, see (Miura et al., 1988)). This linguistic characteristic, also termed *transparency of power*, facilitates learning to count (Lê & Noël, 2020; Miller et al., 1995; Miller & Stigler, 1987) and solving arithmetic problems (Geary et al., 1996; McClung & Arya, 2018; Rodic et al., 2015). Another morpho-syntactic difference concerns the inversion between tens and ones as for example in Dutch and in German (*i.e.* y + *10*x*; in German 42 is “zwei-und-*vierzig*”, literally “two-and-*forty*”). This linguistic characteristic, called *transparency of order* (Bahnmueller et al., 2018), explains some transcoding error patterns and reaction times (Clayton et al., 2020; Imbo et al., 2014; Moeller et al., 2015; Pixner et al., 2011; Poncin et al., 2019; Steiner et al., 2021; Zuber et al., 2009), gives rise to specific pattern of compatibility effects (Bahnmueller et al., 2015; H. Nuerk et al., 2005; H.-C. Nuerk et al., 2004) and complicates the solution of certain arithmetic problems (Göbel et al., 2014; Lonnemann & Yan, 2015; Xenidou-Dervou et al., 2015).

*Transparency of power* can also vary with a change of base, for example a base-20 system (20**x + y*), also called vigesimal. The use of the vigesimal system is found for example in French, Basque, or in Diola-Fogny (see (Haspelmath et al., 2005)). French, to be precise, has a mixed system since it uses base-10 and base-20 systems. Up to the `60s in French, like in English, the counting system follows the base-10 rule. However, `80s to `90s follow a base-20 system, *e.g.* 87 = *4*20 + 7,* “*quatre-vingt*-sept”, literally “*four-twenty*-seven”.

Moreover, the teens (11 to 16) are lexical primitives, like in English and in stark contrast to the more transparent Asian languages. Note that the transparency contrast is additionally increased for 71 to 76 and 91 to 96, which are composed with a base-20 and teens, *e.g.* 96 = 4*20 + *16*, “quatre-vingt-*seize*”, literally “four-twenty-*sixteen*”. Furthermore, the vigesimal system is subject to regional variances, for example in Belgian Wallonia `70s and ‘90s are not vigesimal (i.e. “*septante*” for “*seventy*” and “*nonante*” for “*ninety*”, see (Seron & Fayol, 1994)) and in some Swiss-French cantons the vigesimal system is entirely absent (*i.e.* even for 80, “*huitante*”*,* literally “*eighty*”).

C 2. *I also doubt if ‘transparency of power’ is the best term. It sounds nice/interesting but it’s only the names of the tens that are affected, not the hundreds or thousands (admittedly, it returns for tens of thousands, but still). So isn’t it just ‘transparency of tens/decades’?*

**A.2.** We thank the reviewer for this comment, we decided to use this terminology in the sense that it has been proposed in (Bahnmueller et al., 2018; Dowker & Nuerk, 2017), which is to “*derive the power of each number directly from the number word*” which we believe would include the concept of “*transparency of tens*”. In that sense vigesimal number words in French would fit with this definitions since, “quatre-vingt” (80), literally “four-twenty”, does not provide a direct way to derive the corresponding eight decimal power.

C 3. *Line 173 ‘These results support the above-mentioned TCM stating that precise numbers are encoded in a language dependent format [63].’ I was under the impression that the results mentioned above all related to studies that offered math in a verbal format to find differences in language of presentation. For instance, the text speaks of ‘problems presented in the LM+’. But if this is the case, the conclusion above cannot be drawn. The use of a verbal context means we can’t know if the precise numbers (by the way, does ‘precise numbers’ refer to exact quantities or symbolic numbers?) are stored in a language-dependent or -independent format.*

**A.3.** We understand the reviewers comment concerning the verbal format of the cited studies which would elicit a verbal context. However one of the studies mentioned compares LM Basque and LM Spanish using arabic digits (Salillas & Carreiras, 2014). Nevertheless, we have toned down the formulation by changing the following sentence (line 178-179)

These results tend to support the above-mentioned TCM stating that precise numbers are encoded in a language-dependent format (Dehaene et al., 1999).

C 4. *Line 177 ‘However, they agree with recent reports from the bilingualism literature that academic knowledge acquired in one language is not (or difficultly) transferable to the other language [55].’ That is a bold statement! That basically says I can’t use the knowledge I learn in English in my native language? Luckily this is not true. This study says that there is a switch cost between languages in math problem solving. A switch cost in mathematics is quite far away from this conclusion that in general academic knowledge learned in one language is not transferable to the other language!*

**A 4.** We thank the reviewer for this comment, we understand that the statement is misleading compared to what we wanted to express. We have adjusted the sentence as follows (line 181-183).

However, they agree with recent reports from the bilingualism literature that academic knowledge acquired in one language is retrieved more efficiently in the learning language compared to another language (Volmer et al., 2018).

C 5. *Together it would be nice to see a better paragraph explaining the present research question to earlier knowledge. What is it that we don’t know and how does study contribute to filling that gap?*

**A 5.** We thank the reviewer for this suggestion. We have added a paragraph explaining the research question in the context of previous research about mathematics in Luxembourgish samples (see C 8.). We hope that this additional context information will help to better understand the relation with our previous studies as described just below (lines 192-198).

C 6. *Line 212: The two tasks might thus reveal a somewhat different result pattern, as already observed in Van Rinsveld et al. [69]. Please elaborate on how.*

**A 6.** We thank the reviewer for this comment. We have adjusted the sentence according to the suggestion as follows (lines 221-223):

The two tasks might thus reveal a somewhat different result pattern such as more marked linguistic influences in the reading aloud free recall task than on the verbal-visual recognition task, as already observed in Van Rinsveld et al. (Van Rinsveld et al., 2016).

C 7. *Present study: I said this before, when describing the population also describe the mother tongue of the children and a bit more of the language background in Luxembourgh besides language in the classroom.
     Specifically it is relevant to know and possibly not widely known by the audience that Luxembourgish strongly resembles German. So one may argue that for the 70 German- and Luxembourgish speaking children, LM1 and L1 are (virtually) identical in terms of number words, whereas for the other 27 this isn’t the case. That means there are two linguistically very diverse groups of participants. It would at least be relevant to know at what age the immigrant children had started learning German and French.
     This information is also relevant because in the way the study is framed now, the youngest age group wouldn’t be able to do the task in French at all because they wouldn’t know it yet. But they do. But how well? And to what degree can all results be explained by just very poor proficiency in one group that hasn’t properly been taught French at all?*

**A 8.** We thank the reviewer for this comment, we have adapted the first paragraph of the present study in order to add more information about the linguistic charachteristics of the sample that followed the Luxembourgish school curricula. Regarding the Luxembourgish speakers, this might probably have an influence in terms of L1 to LM1 transfer. As we have answered in the previous review, the sub-sample was unfortunately to small and not equally represented across the age groups to be compared separatly (lines 192– 198).

In the Luxembourgish multilingual school system, pre-schools (3 to 5 y.o.) are in Luxembourgish, which is linguistically close to German. The teaching language in primary school (1^st^ to 6^th^ grade, 6 to 12 y.o.) is German, except for French lessons. From 7^th^ grade to 10^th^ grade, the majority of subjects are taught in German, except for mathematics and French lessons, which are taught in French. Then from 11^th^ grade until the end of obligatory school, all topics are thought in French. This multilingual education system aims to render students highly proficient in both German and French. In sum, critically for number transcoding, the language for teaching and learning mathematics switches from German to French at 7^th^ grade (*Languages in Luxembourg Schools*, 2021). Hence students’ first language of math acquisition (LM1) is German, while their second language of math learning (LM2) is French. Therefore allowing within-subject designs from a sample with the same educational background.

Hypothesizing that non-native Luxembourgish speakers (i.e. immigrants) had more difficulties in learning German than native Luxembourghish, the comparison between German and French still results in a reliable cost.

C 8. *Line 265. There are 2 sets of 5 practice and 14 test stimuli but the experiment lasted 45 minutes. That sounds extremely long for this task. If this test was part of a larger test battery please mention the context.*

**A 8.** We thank the reviewer for this comment. Indeed the experiment was part of a bigger study where the participants passed additional tests (lines 250-253):

Stimuli for each task were 28 different two-digit numbers ranging from 31 to 98. These were further subdivided into two sets of 14 distinct stimuli each, one for the German and the other for the French blocks. The experiment was part of a larger study, which lasted 45 minutes, at the end of which the participants were compensated with a gift voucher.

C 9. *Line 242 still makes the cut at 60 instead of 70. (there are no 14 numbers above and 14 below 60 in the set by the way. There are 4 from each decade). Also explain this decision to leave out the 60 numbers earlier than the results section (it’s not a result, it’s a methodological choice).*

**A 9.** We thank the reviewer for this comment. We thank for noticing this error in the stimuli description which we have corrected and adjusted adding further clarification information, see lines 270-272 above.

C 10. *Line 285. Have you considered post-error slowing (the ‘oops’ effect)? It may be wise to remove RTs after an error too.*

**A11.** We thank the reviewer for this comment. Note that incorrect responses are excluded from the reaction time analyses of both tasks, hence excluding possible “post-error” slowed down trials (lines 394-397).

Finally, for reaction time analyses all incorrect responses were removed, additionally removing 2.93 % of the reading task and 3 % of the matching task, corresponding to the total error rate.

C 11. *Could you give some descriptive statistics? Like M and SD of RT/accuracy per task, broken down by the relevant factors, so by age group, language and number size.*

**A.11.** We thank the reviewer for this comment. We have added the measured mean differences in reaction times where suggested. Note that the break down tables for each level’s by means and standard errors for reaction time and accuracy are in the supplementary materials. (Below, we have removred the statistical results within the text and kept in the table only, according with reviewer 1 suggestion). For example (line 334 – 337, but also lines 352, 353, 385, 388,434,436,459,467):

The model for the reading aloud task RTs resulted in three significant main effects and three two-way interactions (see Table 1). The main effect of Age was decomposed with follow-up analyses showing that the 5^th^ graders (1091*(290)* ms) were slower than the older groups, which had similar RT’s (8^th^ 797*(288)* ms, 11^th^ 780*(247)* ms and adults 828*(320) ms*; standard deviations in parenthesis).

As well in lines 422-429. Note that we changed from Walden z to t-tests given that we used Satterthwaite approximations and that readers might be more familiar with (see R2 C8.c and A8c.):

Follow-up analyses were applied comparing the estimated marginal means with paired comparisons on Satterthwaite corrected degrees of freedom and Bonferroni adjusted p-values. Follow-up analyses confirm the hypothesis relating to *transparence of power,* given a cost in French for ’70s, ’80s and ’90s compared to ’30s, ’40s and ’50s numbers for all age groups: 5^th^ graders with a 593 ms cost ($t(98.0)=9.48,p<.001$), 8^th^ graders 364 ms cost ($t(61.0)=5.34,p<.001$), 11^th^ graders a 356 ms cost ($t(70.7)=3.28,p<.01$), and Adults with a 219 ms cost ($t\left( 87.2 \right)=3.16,p=.01$). In contrast, the same comparison in German did not result in any significant differences ($all t<1.53, p>.76, max difference= 34 ms$). In sum, the cost for vigesimal numbers observed in the reading aloud task is replicated with the verbal-visual matching task for all age groups, see Fig 5.

C 12. *306 we aimed to model the individual variability, ideally considering individual differences (i.e. Subject) and age group (i.e. Age) related differences. Furthermore, we aimed to model per-item variability (i.e. Item), particularly related to language.
      I’m a bit confused here on what exactly you did especially with age group. Is this about random intercepts or random slopes? Slopes for what? And you modeled age group as a fixed effect already so you can’t add a random effect for it too, so it makes sense that that didn’t work.
      So what did the model syntax look like exactly?
      It sounds like it was something like RT~Age*Language*Number Size + (1|Subject) + (1+language|Item)?
      Or also (1+language|Subject)? If not, why no random slope for language per subject given that you say you run a maximal model?*

**A.12** We thank the reviewer for this comment which joins also some of the first reviewers concerns (see R1 C7). We will explain herein into details how we designed and implemented the model. The initial maximal model that we had defined was the following:

RT ~ 1 + Age*Language*Size + (Age|Subject) + (1 + Language|Item)

The reasoning was to have (Age|Subject) as random part reasoning that subjects variability would mostly depend on age group (i.e. different speed of processing, as well as different variabilities). The (1 + Language|Item) random part was designed to catch the number words different lengths and/or phonological characteristics further depending on Language characteristis (given that word length has been brought up as a confounder in bilingual designs (Ellis & Hennelly, 1980)). However the maximal model did not converge (despite trying different optimizers, increase the iterations to 1,000,000: we tried to “fix the convergence” following some common advices as the reviewer lists in C15). Since the model kept not converging, we tried removing different random parts of the maximal model. With this procedure, we reached to the final model Mend (the one presented in the results):

- MM <-	RT ~ 1 + Age*Language*Size + (1 + Age| Subject) + (1 + Language|Item)
- M1 <-	RT ~ 1 + Age*Language*Size + (1 + Age|| Subject) + (1 + Language| Item)
- M2 <-	RT ~ 1 + Age*Language*Size + (1 + Age| Subject) + (1 + Language|| Item)
- M3 <-	RT ~ 1 + Age*Language*Size + (1 + Age|| Subject) + (1 + Language|| Item)
- M4 <-	RT ~ 1 + Age*Language*Size + (1|Subject) + (1 + Language|| Item)
- Mend <- RT ~ 1 + Age*Language*Size + (1| Subject) + (1 + Language| Item)

(Besides M4 and Mend, none of the above model did converge). The Mend model was judged satisfactory among the converging final model because it has a per-item random part, and a per-subject random part (though “only” for intercepts). The model’s R^2^ fit of .57 for the naming and .62 for the matching model) was also judged satisfactory (calculated with the package “MuMIn”although we do not report it in the manuscript given the following issues with effect sizes in linear mixed models: <https://afex.singmann.science/forums/topic/compute-effect-sizes-for-mixed-objects>). Therefore we did not search for further model possibilities. However, concerning the manuscript, as suggested by reviewer 1 (R2 C7 & A7), we removed the different models that were tried as well as the maximal model description in the results so to render the reading of the reults possibly more fluid and kept only a short sentence about how we obtained the final model (lines 301-302)

When our initially designed full model failed to converge, we reduced the model complexity by gradually simplifying the random effect structure until convergence was reached.

We have changed the paragraph about the model description as follows (lines 321-332):

The linear mixed model was defined with main effects and interactions between the fixed between-group factor *Age* (levels: 5^th^, 8^th^, 11^th^ grade and adults) and two fixed within-group factors: *Language* (German, French), and *Number Size* (Large, Small), all levels being defined as categorical. As random factors, we modelled individual differences (*i.e. Subject*) and item-related variability (*i.e. Item*), the latter being considered separately by language. Individual differences were modelled using random intercepts per subject. Item-related variability was modelled using both random intercepts and slopes as a function of languages. In sum, the model takes the following R syntax form (A):

$\left( A \right) RT \sim1 + Age*Language*Number Size + (1|Subject) + (1 + Language|Item)$*.*

P-values and degrees of freedom were obtained with the Satterthwaite approximation method by comparing the full model against the model without the effect (Singmann et al., 2020). Follow-ups were calculated by comparing estimated marginal means (EMM) obtained with the emmeans package (Lenth, 2021), and p-values were adjusted with the Bonferroni method.

And adapted lines 403-405 in the verbal-visual matching task:

For coherence of interpretation and comparison with the previous verbal-visual matching task, the same model and parameters for p-values, degrees of freedom and follow-up’s were applied here on RT.

C 13. *Nd could you tell which method you used to obtain p-values? Conditional F-tests with Kenward-Roger or Satterthwaite corrected degrees of freedom probably?*

**A 13.** We thank the reviewer for this comment. Satterthwaite degree of freedom approximation was used here. (Note that the comparison with Kenward-Rogers method did not lead to big differences in terms of *df* for our dataset, typically third or fourth digit position, but uses significantly longer computation time than Satterthwaite approximation), see cited paragraph above (lines 329-332, see also the note of Table 1).

C 14. *Lines 314, 325 and also in later sections. Specify how you did your follow-up analyses when applicable (and what were the resulting statistics)? Explain what kind of analysis (the same?) on what kind of subset of the data. I see no more F tests but z-scores, if these are Wald tests from mixed effects models, those aren’t very reliable.*

**A.14.** We thank the reviewer for noticing this. The follow-up’s were computed comparing estimated marginal means from the model (from the package emmeans), we have added this information below (lines 348-350):

Follow-up analyses were performed on the model's estimated marginal means (EMMs) of the interaction's terms [see 77] with Satterthwaite estimation of degrees of freedom. Then EMM were compared with two-tailed pairwise tests with Bonferroni adjusted p-values

C 15. *Line 347 and beyond, what do you mean that the model did not fit? Do you mean that it did not converge? Why specifically drop age here, it seems the most relevant factor? I also learned that dropping a random slope is the very very last thing one should try as it greatly reduces p-values (so it inflates significant findings).* [*https://academic.oup.com/esr/article/35/2/258/5306121*](https://academic.oup.com/esr/article/35/2/258/5306121)*. Now non-convergence can be a beast but other solutions to solve it do exist. This is what I learned to try, in this order:
      – Most recent package versions (particularly lme4)?
      – Center instead of standardize (or vice versa) cont. predictors
      – Increase number of iterations
      – Restart model fitting from previous (unconverged) estimates
      – Different optimizer(s); compare estimates from many different optimizers; (compare to) brms model (Bayesian model)
      - Simplify your model, Barr et al.: remove: (a) random correlations (b) random intercepts (c) random slopes; last resort, try to avoid that!
      By the way, alternatively I could also imagine leaving accuracy out altogether because of its ceiling effects.*

**A 15.** We thank the reviewer for this very detailed and informed recommendation. We tried the most of these recommendations as described below, but honestly, we are not even sure how to interpret the mixed model’s results (if they converged) given the ceiling effect. As reported in S1 Table 2 and 4, there are some cells with 100% accuracies and other with only errors in only one or two trials. The task is not hard enough to induce participants in enough errors, making this measure insensible. Nevertheless, we tried to fit the model again with (1) the updated to the latest lmer package (i.e. version 1.1-27), (2) we increased the number of iterations to 1e6 (3) tried different optimizers (with all_fit = TRUE of the mixed package). Finally, the model is already as simplified as possible, so that removing an additional term would then also remove the meaning of doing the model . We could not center nor standardize the predictors, since the dependent variable accuracy is binomial.

C 16. *Line 358 in order to explain the interaction language * number size you give the results for French but also give the contrasting results for German (I guess no sig difference? But I shouldn’t need to guess.)? You need them both to understand the interaction.*

**A.16** We thank the reviewer for this comment. We have added the results for the contrast in German as suggested (lines 385-388):

Follow-up analyses confirmed with CR the pattern observed with RT: in French, 4.8% more errors were made with ’70s, ’80s and ’90s than ’30s, ’40s and ’50s numbers $\left( z=-3.37,p<.01 \right)$, indicating a cost for vigesimal number transcoding. The same contrast in German did not lead to any significant difference$\left( z=0.63, n.s. \right).$

C 17. *p. 17 I’m a bit surprised that the language difference is so weak given the highly significant effect in table 3 and also the large differences in the figures. Is this correct?
      The analyses with only 4 syllable words made me wonder how to count syllables in French reliably given that so many of them are silent. If I look it up there seems to be consensus that silent e’s at the end of a word don’t count as syllables meaning that many words are one syllable shorter.*

**A 17.** We thank the reviewer for this comment. We were also surprised to observe that the language effect is not very stable across ages given the figures, we have added the estimated differences. We re-runned the follow-up analyses with three different p-value adjustments (holm, Bonferroni, and free as advised here: <https://cran.r-project.org/web/packages/afex/vignettes/afex_mixed_example.html#follow-up-analyses>) to test the significance stability. The significancy patterns are confirmed by all three types of adjustments, only the 11^th^ grade group fluctuates around the alpha level, depending on the adjustments done, with the Bonferonni (the one in the manuscript) being the less significant and most conservative p-value.

| Age group: | Contrast: | Holm | Bonferroni | Free |
| --- | --- | --- | --- | --- |
|  | | | | |
| 5^th^ | Large(FR)-small(FR) | <.0001 | <.0001 | <.0001 |
|  | **Small(FR)-small(GE)** | **<.0001** | **<.0001** | **<.0001** |
|  | Large(GE)-small(GE) | 0.127 | 0.7617 | 0.4185 |
|  | | | | |
| 8^th^ | Large(FR)-small(FR) | <.0001 | <.0001 | <.0001 |
|  | **Small(FR)-small(GE)** | **0.091** | **0.182** | **0.1319** |
|  | Large(GE)-small(GE) | 0.8192 | 1 | 0.9957 |
|  | | | | |
| 11th | Large(FR)-small(FR) | 0.0064 | 0.0097 | 0.0086 |
|  | **Small(FR)-small(GE)** | **0.0369** | **0.0737** | **0.0589** |
|  | Large(GE)-small(GE) | 0.497 | 1 | 0.9039 |
|  | | | | |
| Adults | Large(FR)-small(FR) | 0.0086 | 0.0129 | 0.0113 |
|  | **Small(FR)-small(GE)** | **0.4086** | **0.8172** | **0.4423** |
|  | Large(GE)-small(GE) | 0.7571 | 1 | 0.9758 |
|  | | | | |
| Note: FR: French, GE: German. Large: ’70s ‘80s and ‘90s, small:’30s, ‘40s and ‘50s. In bold the contrasts concerning the language difference. | | | | |

Note that we had obtained a similar follow-up pattern when running the analyses with ANOVA (cfr first manuscript). Also note that this observation was the motivation behind running the same analyses on standardized z-scores (as it would control for the bigger variability observed in 5^th^ grade, see standard error in the figure and SD in the table). Indeed the z-score turn out to be very similar across age groups (very “flat lines” in the figure), suggesting that the non-significant effects with RT might be inflated by the variability in RT responses by the younger age group.

Second, concerning the silent “e” at the end of the number words, we replicated the analyses by counting them as silent or not (i.e. 4 syllables for soi/xan/te/quinze). By counting them as silent we replicated for the naming task, the Language x Number Size interaction. For the matching task we replicate the three-way interaction. Note that, theoretically, item variability is also included now into the linear mixed model’s per-items random slopes and intercepts.

C 18. *Concerning this language difference in the verbal to visual task: isn’t German also easier in the sense of at which point you can already eliminate multiple choice options on the go? If you hear ‘vierund…’ you can already exclude the +1/-1 and the +11/-11 answers, leaving only two possible options halfway through the word. For French, specifically for the large numbers, hearing ‘quatrevingt…’ may still leave 3 or 4 options open halfway through the word.*

**A 18.** We thank the reviewer for this interesting reflection about possible participant’s strategic behaviour. We would like to bring to attention that the auditory and visual stimuli were presented sequentially, not simultaneously hence excluding the possibility for this strategic behaviour to be online. In order for this strategic “pre-selection” to occur, it would need a prior mental representation to have been built up by the participant (likely in verbal working memory, since transcoding is sensible to verbal interference (Frank et al., 2012)). Therefore the verbal to visual transcoding (which is critical for the hypothesis) must have occurred before the hypothetical strategic “pre-selection”. Also, a “pre-selection” would be possible in French by excluding the possibilities with a different unit.

C 19. *Minor points*

**A.19.** We thank the reviewer for noticing the following minor points, which we have fixed accordingly in this revision.

a. *Line 151. comparable results than for problems presented --> comparable results to problems presented. Also in line 194.*

**C.19.a.** see l.156, 203

The results indicate a cost for arithmetical problems written in number words in Filipino (i.e. being the L1 but LM-) compared to English (i.e. being an L2 but LM+), which in turn showed comparable results to problems presented as Arabic digits.

Previous studies on this specific population have reported language effects in magnitude comparison tasks, showing comparable compatibility effects to monolingual German [69].

b. *Line 161/ who had either learned arithmetic in English or Spanish --> who had learned arithmetic in either English or Spanish.*

**C.19.b.** lines 166-167

Recording electroencephalogram during a true or false judgment of simple multiplications, Salillas and Wicha (Salillas & Wicha, 2012) studied fluent Spanish-English bilingual adults who had learned arithmetic in either English or Spanish, the LM+ respectively

c. *Line 227 switches to French (LM2) after the 7th grade --> after 6th grade or in 7th grade.*

**C.19.c.** line 237

Importantly the language support for the formal acquisition of mathematics through school curricula is first in German (LM1) from 1^st^ to 6^th^ grade and switches to French (LM2) in 7^th^ grade.

d. *Line 358 says ‘40s and ‘40s instead of ‘50s.*

**C.19.d.** line 386

’30s, ’40s and ’50s numbers

e. *Quite some typos in the supplementary table with written out number words like ‘soixantetroi’, ‘siebenndneunzig’, etc.*

**C.19.e.** Thank you for noticing those typos! We have corrected them in the present supplementary material (S3 Table 1). i.e. soixantetrois or siebenundneunzig.

# Bibliography:

Bahnmueller, J., Moeller, K., Mann, A., & Nuerk, H.-C. (2015). On the limits of language influences on numerical cognition – no inversion effects in three-digit number magnitude processing in adults. *Frontiers in Psychology*, *6*. https://doi.org/10.3389/fpsyg.2015.01216

Bahnmueller, J., Nuerk, H.-C., & Moeller, K. (2018). A Taxonomy Proposal for Types of Interactions of Language and Place-Value Processing in Multi-Digit Numbers. *Frontiers in Psychology*, *9*. https://doi.org/10.3389/fpsyg.2018.01024

Barrouillet, P., Camos, V., Perruchet, P., & Seron, X. (2004). ADAPT: A Developmental, Asemantic, and Procedural Model for Transcoding From Verbal to Arabic Numerals. *Psychological Review*, *111*(2), 368–394. https://doi.org/10.1037/0033-295X.111.2.368

Campbell, J. (1995). Mechanisms of Simple Addition and Multiplication: A Modified Network-interference Theory and Simulation. *Mathematical Cognition*, *1*, 121–164.

Clayton, F. J., Copper, C., Steiner, A. F., Banfi, C., Finke, S., Landerl, K., & Göbel, S. M. (2020). Two-digit number writing and arithmetic in Year 1 children: Does number word inversion matter? *Cognitive Development*, *56*, 100967. https://doi.org/10.1016/j.cogdev.2020.100967

De Visscher, A., & Noël, M.-P. (2014). The detrimental effect of interference in multiplication facts storing: Typical development and individual differences. *Journal of Experimental Psychology: General*, *143*(6), 2380–2400. https://doi.org/10.1037/xge0000029

Dehaene, S. (1992). Varieties of numerical abilities. *Cognition*, *44*(1), 1–42. https://doi.org/10.1016/0010-0277(92)90049-N

Dehaene, S., Piazza, M., Pinel, P., & Cohen, L. (2003). Three parietal circuits for number processing. *Cognitive Neuropsychology*, *20*(3–6), 487–506. https://doi.org/10.1080/02643290244000239

Dehaene, S., Spelke, E., Pinel, P., Stanescu, R., & Tsivkin, S. (1999). Sources of Mathematical Thinking: Behavioral and Brain-Imaging Evidence. *Science*, *284*(5416), 970–974. https://doi.org/10.1126/science.284.5416.970

Deloche, G., & Seron, X. (1982). From three to 3: A differential analysis of skills in transcoding quantities between patients with Broca’s and Wernicke’s aphasia. *Brain*, *105*(4), 719–733. https://doi.org/10.1093/brain/105.4.719

Dotan, D., & Friedmann, N. (2018). A cognitive model for multidigit number reading: Inferences from individuals with selective impairments. *Cortex*, *101*, 249–281. https://doi.org/10.1016/j.cortex.2017.10.025

Dowker, A., & Nuerk, H.-C. (Eds.). (2017). *Linguistic Influences on Mathematical Cognition*. Frontiers Media SA. https://doi.org/10.3389/978-2-88945-200-2

Ellis, N. C., & Hennelly, R. A. (1980). A bilingual word-length effect: Implications for intelligence testing and the relative ease of mental calculation in Welsh and English. *British Journal of Psychology*, *71*(1), 43–51. https://doi.org/10.1111/j.2044-8295.1980.tb02728.x

Frank, M. C., Fedorenko, E., Lai, P., Saxe, R., & Gibson, E. (2012). Verbal interference suppresses exact numerical representation. *Cognitive Psychology*, *64*(1), 74–92. https://doi.org/10.1016/j.cogpsych.2011.10.004

Geary, D. C., Bow‐Thomas, C. C., Liu, F., & Siegler, R. S. (1996). Development of Arithmetical Competencies in Chinese and American Children: Influence of Age, Language, and Schooling. *Child Development*, *67*(5), 2022–2044. https://doi.org/10.1111/j.1467-8624.1996.tb01841.x

Göbel, S. M., Moeller, K., Pixner, S., Kaufmann, L., & Nuerk, H.-C. (2014). Language affects symbolic arithmetic in children: The case of number word inversion. *Journal of Experimental Child Psychology*, *119*, 17–25. https://doi.org/10.1016/j.jecp.2013.10.001

Haspelmath, M., Dryer, M. S., Gil, D., & Comrie, B. (2005). *The World Atlas of Language Structures*. Oxford Univ. Press.

Imbo, I., Vanden Bulcke, C., De Brauwer, J., & Fias, W. (2014). Sixty-four or four-and-sixty? The influence of language and working memory on children’s number transcoding. *Frontiers in Psychology*, *5*. https://doi.org/10.3389/fpsyg.2014.00313

*Languages in Luxembourg schools*. (2021). http://men.public.lu/en/themes-transversaux/langues-ecole-luxembourgeoise.html

Lê, M.-L. T., & Noël, M.-P. (2020). Transparent number-naming system gives only limited advantage for preschooler’s numerical development: Comparisons of Vietnamese and French-speaking children. *PLOS ONE*, *15*(12), e0243472. https://doi.org/10.1371/journal.pone.0243472

Lenth, R. V. (2021). *emmeans: Estimated marginal means, aka least-squares means* [Manual]. https://CRAN.R-project.org/package=emmeans

Lonnemann, J., & Yan, S. (2015). Does number word inversion affect arithmetic processes in adults? *Trends in Neuroscience and Education*, *4*(1), 1–5. https://doi.org/10.1016/j.tine.2015.01.002

Luke, S. G. (2017). Evaluating significance in linear mixed-effects models in R. *Behavior Research Methods*, *49*(4), 1494–1502. https://doi.org/10.3758/s13428-016-0809-y

McCloskey, M. (1992). Cognitive mechanisms in numerical processing: Evidence from acquired dyscalculia. *Cognition*, *44*(1–2), 107–157. https://doi.org/10.1016/0010-0277(92)90052-J

McCloskey, M., Caramazza, A., & Basili, A. (1985). Cognitive mechanisms in number processing and calculation: Evidence from dyscalculia. *Brain and Cognition*, *4*(2), 171–196. https://doi.org/10.1016/0278-2626(85)90069-7

McClung, N. A., & Arya, D. J. (2018). Individual Differences in Fourth-Grade Math Achievement in Chinese and English. *Frontiers in Education*, *3*, 29. https://doi.org/10.3389/feduc.2018.00029

Miller, K. F., Smith, C. M., Zhu, J., & Zhang, H. (1995). Preschool Origins of Cross-National Differences in Mathematical Competence: The Role of Number-Naming Systems. *Psychological Science*, *6*(1), 56–60.

Miller, K. F., & Stigler, J. W. (1987). Counting in Chinese: Cultural variation in a basic cognitive skill. *Cognitive Development*, *2*(3), 279–305. https://doi.org/10.1016/S0885-2014(87)90091-8

Miura, I. T., Kim, C. C., Chang, C.-M., & Okamoto, Y. (1988). Effects of Language Characteristics on Children’s Cognitive Representation of Number: Cross-National Comparisons. *Child Development*, *59*(6), 1445. https://doi.org/10.2307/1130659

Moeller, K., Zuber, J., Olsen, N., Nuerk, H.-C., & Willmes, K. (2015). Intransparent German number words complicate transcoding – a translingual comparison with Japanese. *Frontiers in Psychology*, *6*. https://doi.org/10.3389/fpsyg.2015.00740

Nuerk, H., Weger, U., & Willmes, K. (2005). Language effects in magnitude comparison: Small, but not irrelevant. *Brain and Language*, *92*(3), 262–277. https://doi.org/10.1016/j.bandl.2004.06.107

Nuerk, H.-C., Weger, U., & Willmes, K. (2004). On the Perceptual Generality of the Unit-Decade Compatibility Effect. *Experimental Psychology*, *51*(1), 72–79. https://doi.org/10.1027/1618-3169.51.1.72

Pixner, S., Zuber, J., Heřmanová, V., Kaufmann, L., Nuerk, H.-C., & Moeller, K. (2011). One language, two number-word systems and many problems: Numerical cognition in the Czech language. *Research in Developmental Disabilities*, *32*(6), 2683–2689. https://doi.org/10.1016/j.ridd.2011.06.004

Poncin, A., Van Rinsveld, A., & Schiltz, C. (2019). Units-first or tens-first: Does language matter when processing visually presented two-digit numbers? *Quarterly Journal of Experimental Psychology*, *73*(5), 726–738. https://doi.org/10.1177/1747021819892165

Power, R. J. D., & Dal Martello, M. F. (1990). The dictation of Italian numerals. *Language and Cognitive Processes*, *5*(3), 237–254. https://doi.org/10.1080/01690969008402106

Rodic, M., Zhou, X., Tikhomirova, T., Wei, W., Malykh, S., Ismatulina, V., Sabirova, E., Davidova, Y., Tosto, M. G., Lemelin, J.-P., & Kovas, Y. (2015). Cross-cultural investigation into cognitive underpinnings of individual differences in early arithmetic. *Developmental Science*, *18*(1), 165–174. https://doi.org/10.1111/desc.12204

Salillas, E., & Carreiras, M. (2014). Core number representations are shaped by language. *Cortex*, *52*, 1–11. https://doi.org/10.1016/j.cortex.2013.12.009

Salillas, E., & Wicha, N. Y. Y. (2012). Early Learning Shapes the Memory Networks for Arithmetic: Evidence From Brain Potentials in Bilinguals. *Psychological Science*, *23*(7), 745–755. https://doi.org/10.1177/0956797612446347

Seron, X., & Fayol, M. (1994). Number transcoding in children: A functional analysis. *British Journal of Developmental Psychology*, *12*(3), 281–300. https://doi.org/10.1111/j.2044-835X.1994.tb00635.x

Singmann, H. (2021). *Mixed Model Reanalysis of RT data*. https://cran.r-project.org/web/packages/afex/vignettes/afex_mixed_example.html

Singmann, H., Bolker, B., Westfall, J., Aust, F., & Ben-Shachar, M. S. (2020). *afex: Analysis of factorial experiments*. https://CRAN.R-project.org/package=afex

Singmann, H., & Kellen, D. (2019). An Introduction to Mixed Models for Experimental Psychology. In D. Spieler & E. Schumacher (Eds.), *New Methods in Cognitive Psychology* (1st ed., pp. 4–31). Routledge. https://doi.org/10.4324/9780429318405-2

Steiner, A. F., Banfi, C., Finke, S., Kemény, F., Clayton, F. J., Göbel, S. M., & Landerl, K. (2021). Twenty-four or four-and-twenty: Language modulates cross-modal matching for multidigit numbers in children and adults. *Journal of Experimental Child Psychology*, *202*, 104970. https://doi.org/10.1016/j.jecp.2020.104970

Van Rinsveld, A., Schiltz, C., Landerl, K., Brunner, M., & Ugen, S. (2016). Speaking two languages with different number naming systems: What implications for magnitude judgments in bilinguals at different stages of language acquisition? *Cognitive Processing*, *17*(3), 225–241. https://doi.org/10.1007/s10339-016-0762-9

Volmer, E., Grabner, R. H., & Saalbach, H. (2018). Language switching costs in bilingual mathematics learning: Transfer effects and individual differences. *Zeitschrift Für Erziehungswissenschaft*, *21*(1), 71–96. https://doi.org/10.1007/s11618-017-0795-6

Xenidou-Dervou, I., Gilmore, C., van der Schoot, M., & van Lieshout, E. C. D. M. (2015). The developmental onset of symbolic approximation: Beyond nonsymbolic representations, the language of numbers matters. *Frontiers in Psychology*, *6*. https://doi.org/10.3389/fpsyg.2015.00487

Zuber, J., Pixner, S., Moeller, K., & Nuerk, H.-C. (2009). On the language specificity of basic number processing: Transcoding in a language with inversion and its relation to working memory capacity. *Journal of Experimental Child Psychology*, *102*(1), 60–77. https://doi.org/10.1016/j.jecp.2008.04.003
