# Supplementary material for: Number transcoding in bilinguals—A transversal developmental study
Source: PLoS One. 2022 Aug 29;17(8):e0273391. doi: 10.1371/journal.pone.0273391 (PMC9423630; doi:10.1371/journal.pone.0273391)
Supplement: Attachment — Submitted filename: 220328 Review 3 (revision 2).docx [file pone.0273391.s007.docx]

*I read the new version of the manuscript and I don’t have any major remarks about the introduction, they have been adequately addressed, just a few small points. However, unfortunately I see that the analysis was still not carried out correctly and that could have large consequences, which requires a revision of the analysis. I’ll explain in more detail below.*

The initial maximal model that we had defined was the

following:

RT ~ 1 + Age*Language*Size + (Age|Subject) + (1 + Language|Item)

*I understand that this model and most of its varieties did not converge until you removed this random slope, because what you did, modeling (Age|Subject), is impossible. You seem to be misunderstanding what a random slope means. What you are really specifying here is that you are allowing the strength of the effect of age to vary across subjects. But that does not make sense in your design. Age does not differ within subjects, so you can’t have an individual effect of age for a single participant. That would require longitudinal data.*

*So you could (actually, should) have a random slope of age|item (allowing age group differences to vary across items) but not age|subject.*

*Likewise, you can, and actually should, include a random slope of size|subject whereas size|item would be equally nonsensical as age|subject.*

*Furthermore, you have multiple measures of language for both the subjects and the items, so including language|item but not language|subject seems arbitrary. Especially given the diverse linguistic backgrounds of the children, it is likely that language effects vary across subjects.*

*A maximal model contains all the random slopes that are possible (and no random slopes that are impossible), so you haven’t actually run the maximal model.*

*If you have a random intercept only but no (or not all) random slope(s), then what you specify is that participants show individual variation in their overall RT (assuming you used sum-to-zero contrasts, please specify if you did, because if not the main effects mean something different), but language and size affect their RTs identically for everyone. And similarly, items are allowed to differ in overall RT and the language effect is allowed to vary across items, but age effects are specified to be exactly identical across items. These assumptions aren’t very likely to hold, and simulation studies have shown that this tends to yield p-values that are far too low. They can even be underestimated by a factor 10 (see the frightening ‘Type 1’ column of tables 5 and 6 from Barr, Levy, Scheepers and Tily, 2013, that shows that omitting random slopes can inflate type 1 error rate to as much as .44 instead of .05). Barr (2013) (same author, but not in any sense related to me, just to clarify) even showed that you also need to incorporate random slopes for the highest possible order interaction term, and that having a maximal model except for the interaction terms was surprisingly the worst-performing model.*

*Keeping all this in mind, this would yield the following maximal model:*

RT ~ 1 + Age*Language*Size + (1+ Language*Size|Subject) + (1 + Age*Language|Item)

Barr, D. J. (2013). Random effects structure for testing interactions in linear mixed-effects models. *Front Psychol*, *4*, 328. <https://doi.org/10.3389/fpsyg.2013.00328>

Barr, D. J., Levy, R., Scheepers, C., & Tily, H. J. (2013). Random effects structure for confirmatory hypothesis testing: Keep it maximal. *Journal of Memory and Language*, *68*(3). https://doi.org/10.1016/j.jml.2012.11.001

*A few minor points:*

C2 I also doubt if ‘transparency of power’ is the best term. It sounds nice/interesting but

it’s only the names of the tens that are affected, not the hundreds or thousands

(admittedly, it returns for tens of thousands, but still). So isn’t it just ‘transparency of

tens/decades’?

A.2. We thank the reviewer for this comment, we decided to use this terminology in the

sense that it has been proposed in (Bahnmueller et al., 2018; Dowker & Nuerk, 2017),

which is to “derive the power of each number directly from the number word” which we

believe would include the concept of “transparency of tens”. In that sense vigesimal

number words in French would fit with this definitions since, “quatre-vingt” (80), literally

“four-twenty”, does not provide a direct way to derive the corresponding eight decimal

power.

*I understand that, my point was that referring to this phenomenon as ‘transparency of power’ is a bit general since only a few tens are affected (70/80/90), and all the higher powers are fully transparent. (for example 800 is huit cent, and 8000 is huit mille, there is nothing vigesimal going on there.) Perhaps ‘transparency of power in the tens’ would better capture it but I understand that that doesn’t sound very catchy.*

C10 Line 285. Have you considered post-error slowing (the ‘oops’ effect)? It may be

wise to remove RTs after an error too.

A10. We thank the reviewer for this comment. Note that incorrect responses are

excluded from the reaction time analyses of both tasks, hence excluding possible

“post-error” slowed down trials (lines 394-397).

Finally, for reaction time analyses all incorrect responses were removed, additionally

removing 2.93 % of the reading task and 3 % of the matching task, corresponding to

the total error rate.

*That is not post-error slowing. Post means after. So as I said, post-error slowing refers to the reaction times for the trial right AFTER an error. They tend to be slower because the participant often realizes they just made a mistake and they are distracted. That is the oops effect.*

*Concerning the model syntax, to be clear, I didn’t necessarily want the whole syntax written down in R language in the manuscript, I put it here as a clarifying question in the review as to find out what you did exactly because the text in the previous version was ambiguous. But if you prefer you can also describe the random slopes and intercepts verbally, as not every reader will be familiar with the specifics of R syntax. It does show really transparently what you did though, so I’m not saying it’s a bad thing, if that’s your choice it’s fine. Just to clear up a possible misunderstanding that you don’t only put it in because I said so, because that was not my intention.*
