# Supplementary material for: Number transcoding in bilinguals—A transversal developmental study
Source: PLoS One. 2022 Aug 29;17(8):e0273391. doi: 10.1371/journal.pone.0273391 (PMC9423630; doi:10.1371/journal.pone.0273391)
Supplement: Attachment — Submitted filename: Response to Reviewers_Revision3.docx [file pone.0273391.s008.docx]

Dear Editor, Prof. Dr Prado,

dear Reviewer,

We thank again the reviewer for their constructive comments on the second revision’s manuscript. Following those comments, we have made two major changes in the result part: (1) we wrote the results anew with the model suggested in C1 and (2) we removed trials corresponding to post-error slow-down (i.e. trials following an incorrect response). Therefore, those changes required writing the descriptive data anew (that is all tables, figures, and supplementary analyses), which can be found in the present revision. However, the significance patterns described in the revision 1 and 2 remained largely unchanged. The only change in significance was a now significant three-way interaction between age, number size, and language in the number reading task. This three-way interaction, however, is not significant with the two additional analyses on *z-scores* and subset data of 4-syllable long number words. Accordingly, we only made a few minor changes in some corresponding formulations in the discussion and in one sentence of the abstract.

# Reviewer #1:

**C1.** All my comments have been addressed.
I just found one typo -- line 378, “suject” should be “subject”.

**A2.** We thank the reviewer for noticing this typo, it has been corrected in the current revision.

# Reviewer #2:

**C1***. I read the new version of the manuscript and I don’t have any major remarks about the introduction, they have been adequately addressed, just a few small points. However, unfortunately I see that the analysis was still not carried out correctly and that could have large consequences, which requires a revision of the analysis. I’ll explain in more detail below.*

The initial maximal model that we had defined was the following:

RT ~ 1 + Age*Language*Size + (Age|Subject) + (1 + Language|Item)

*I understand that this model and most of its varieties did not converge until you removed this random slope, because what you did, modeling (Age|Subject), is impossible. You seem to be misunderstanding what a random slope means. What you are really specifying here is that you are allowing the strength of the effect of age to vary across subjects. But that does not make sense in your design. Age does not differ within subjects, so you can’t have an individual effect of age for a single participant. That would require longitudinal data.*

*So you could (actually, should) have a random slope of age|item (allowing age group differences to vary across items) but not age|subject.*

*Likewise, you can, and actually should, include a random slope of size|subject whereas size|item would be equally nonsensical as age|subject.*

*Furthermore, you have multiple measures of language for both the subjects and the items, so including language|item but not language|subject seems arbitrary. Especially given the diverse linguistic backgrounds of the children, it is likely that language effects vary across subjects.*

*A maximal model contains all the random slopes that are possible (and no random slopes that are impossible), so you haven’t actually run the maximal model.*

*If you have a random intercept only but no (or not all) random slope(s), then what you specify is that participants show individual variation in their overall RT (assuming you used sum-to-zero contrasts, please specify if you did, because if not the main effects mean something different), but language and size affect their RTs identically for everyone. And similarly, items are allowed to differ in overall RT and the language effect is allowed to vary across items, but age effects are specified to be exactly identical across items. These assumptions aren’t very likely to hold, and simulation studies have shown that this tends to yield p-values that are far too low. They can even be underestimated by a factor 10 (see the frightening ‘Type 1’ column of tables 5 and 6 from Barr, Levy, Scheepers and Tily, 2013, that shows that omitting random slopes can inflate type 1 error rate to as much as .44 instead of .05). Barr (2013) (same author, but not in any sense related to me, just to clarify) even showed that you also need to incorporate random slopes for the highest possible order interaction term, and that having a maximal model except for the interaction terms was surprisingly the worst-performing model.*

*Keeping all this in mind, this would yield the following maximal model:*

RT ~ 1 + Age*Language*Size + (1+ Language*Size|Subject) + (1 + Age*Language|Item)

Barr, D. J. (2013). Random effects structure for testing interactions in linear mixed-effects models. *Front Psychol*, *4*, 328. <https://doi.org/10.3389/fpsyg.2013.00328>

Barr, D. J., Levy, R., Scheepers, C., & Tily, H. J. (2013). Random effects structure for confirmatory hypothesis testing: Keep it maximal. *Journal of Memory and Language*, *68*(3). https://doi.org/10.1016/j.jml.2012.11.001

**A1.** We thank the reviewer for this important and very constructively explained comment about defining the maximal model. We now see the mistake we made in defining the maximal model with random slopes Age|Subjects and Size|Item. In the present revision, we implemented the suggested maximal model for both tasks. However, this model resulted in a singular error for the reading task, likely due to high correlations between random parts of Number Size per subject and Age per item. Therefore, we removed some terms from the random parts (see lines 325 to 338), aiming to preserve the most comprehensive model possible without singular fit.

The maximum model was defined taking into account individual differences by using random slopes and intercepts *per Subject* for the interaction between *Language* and *Number Size*. Moreover, item-related variability was modelled using random intercepts and slopes *per Item* for the interaction between *Language* and *Age*. This led to the model with the following R syntax form (A0):

$\left( A0 \right) RT \sim1 + Age*Language*Number Size + (1+ Language*Number Size|Subject) + (1 + Language*Age |Item)$*.*

However, since the maximal model led to a singular fit (due to high correlations between the random parts of Number Size per Subject and Age per Item ) we reduced the complexity of the model by removing those problematic random terms [78]. Therefore, the final model takes the following R syntax form (A):

$\left( A \right) RT \sim1 + Age*Language*Number Size + (1+Language|Subject) + (1 + Language|Item)$*.*

This final model led to the same pattern of significance than in our previous manuscript version, except for a significant three-way interaction between Age, Language and Number Size. However, the three-way interaction was neither significant with *z-score* nor with subset dataset of 4 syllable long number words. We therefore preserved our overall interpretation of the results, while adapting the wording in the abstract (lines 31 to 33) and in the discussion (lines 510 to 511 and l. 588-591) to reflect the final result pattern. Furthermore, we adapted the S2 Tables 1 to 6 and follow-up statistics with the values of the current model (additional changes are due to the removal of post-error slowing, see A3.). In addition, during the process of re-calculating and re-writing the results we noticed and corrected some small errors and typos, which we did not note.

*A few minor points:*

**C2. [R2C2]** I also doubt if ‘transparency of power’ is the best term. It sounds nice/interesting but it is only the names of the tens that are affected, not the hundreds or thousands (admittedly, it returns for tens of thousands, but still). So is not it just ‘transparency of tens/decades’?

**[R2A2]:** We thank the reviewer for this comment, we decided to use this terminology in the sense that it has been proposed in (Bahnmueller et al., 2018; Dowker & Nuerk, 2017), which is to “derive the power of each number directly from the number word” which we believe would include the concept of “transparency of tens”. In that sense vigesimal number words in French would fit with this definitions since, “quatre-vingt” (80), literally “four-twenty”, does not provide a direct way to derive the corresponding eight decimal power.

**C2.** *I understand that, my point was that referring to this phenomenon as ‘transparency of power’ is a bit general since only a few tens are affected (70/80/90), and all the higher powers are fully transparent. (for example 800 is huit cent, and 8000 is huit mille, there is nothing vigesimal going on there.) Perhaps ‘transparency of power in the tens’ would better capture it but I understand that that doesn’t sound very catchy.*

**A2.** We thank the reviewer for this additional comment, indeed the suggested “transparency of power in the tens” would be a finer terminology corresponding closer to the language structure and leads to an interesting discussion. To be super-precise it should be named “transparency of power in the tens between seventy and ninety”, since not only hundredths and thousands, but other decades as well are not affected (i.e. 40 is not “deux-vingt”, as it would be in Basque). In (Haspelmath et al., 2005) French is not even considered a decimal system and Basque is considered a hybrid vigesimal-decimal for the reason that 3 and 4 digit numbers follow the decimal structure as pointed out by the reviewer. However, we argue that the linguistic-cognitive interaction would be caused by a “*change of base”* (Ms. line.102). We compare 30/40/50 vs. 70/80/90 in French, where the change of base occurs, so we reason that what we observed is a “transparency of power” phenomenon and therefore prefer to conserve this more concise terminology.

**C2. [see R2C10]** Line 285. Have you considered post-error slowing (the ‘oops’ effect)? It may be wise to remove RTs after an error too.

**R2A10.** We thank the reviewer for this comment. Note that incorrect responses are excluded from the reaction time analyses of both tasks, hence excluding possible “post-error” slowed down trials (lines 394-397). Finally, for reaction time analyses all incorrect responses were removed, additionally removing 2.93 % of the reading task and 3 % of the matching task, corresponding to the total error rate.

***C2.*** *That is not post-error slowing. Post means after. So as I said, post-error slowing refers to the reaction times for the trial right AFTER an error. They tend to be slower because the participant often realizes they just made a mistake and they are distracted. That is the oops effect.*

**A2.** We thank the reviewer for this clarification. Since the accuracy rate is quite high in our study, it makes sense to remove those trials following an error, as they might have been additionally slower. In the present revision, we removed all trials following an error in both tasks (within each block and before we removed the ‘60s to respect the chronological order of presentation in the experiment). This had a slight influence on the descriptive, and we draw the figures anew to exclude post-error slow down trials there too. See lines 296-298:

As suggested by an anonymous reviewer, we additionally removed the trials following an error that might be affected by post-error slowing in particular given the high accuracy rate [75], thus additionally accounting for 1.95 % and 5.63 % of the trials respectively.

We additionally re-wrote the descriptive S1 Tables 1 to 4, since the values changed after removing both incorrect and subsequent trials corresponding to post-error slow-down and corrected some typos.

***C3.*** *Concerning the model syntax, to be clear, I didn’t necessarily want the whole syntax written down in R language in the manuscript, I put it here as a clarifying question in the review as to find out what you did exactly because the text in the previous version was ambiguous. But if you prefer you can also describe the random slopes and intercepts verbally, as not every reader will be familiar with the specifics of R syntax. It does show really transparently what you did though, so I’m not saying it’s a bad thing, if that’s your choice it’s fine. Just to clear up a possible misunderstanding that you don’t only put it in because I said so, because that was not my intention.*

**A3.** We understood it was not the reviewer’s intention to make us report the R syntax. We choose to report it, as it is the most direct way to convey the model, for those familiar with R. In particular, since there are at the moment no standards on how to report linear mixed models.
