# Supplementary material for: Number transcoding in bilinguals—A transversal developmental study
Source: PLoS One. 2022 Aug 29;17(8):e0273391. doi: 10.1371/journal.pone.0273391 (PMC9423630; doi:10.1371/journal.pone.0273391)
Supplement: Attachment — Submitted filename: ResponseToReviewer_Final.docx [file pone.0273391.s009.docx]

Dear Editor Prof. Dr. Prado,

We have carefully considered and discussed the reviewer’s comment about the model selection. Reviewer #2 insists in implementing a model that is as maximal as possible in line with the recommendations in Barr et al. (2013). However, the maximal model, suggested by the reviewer in the 3^rd^ revision, results in a singular fit, requiring to degrade it. The reviewer’s main point of concern in the 4^th^ revision regards the process of how to degrade and select the final model. His/her concern is that too simplified models might result in statistical alpha inflation.

Currently there is no golden standard regarding the model selection process. The absence of agreement in how to select a model is actively debated in the statistical literature, see for example (Bates et al., 2018; Matuschek et al., 2017). Matuschek et al. (2017) thus suggest to use “parsimonious models”, pointing to the limits of the approach of “keep the model maximal” suggested by (Barr et al., 2013). The lack of agreement about model selection was confirmed to us after consulting three different expert statisticians about our model selection (one internal colleague from the University Luxembourg and two external experts from University Potsdam and the University Aix-Marseille).

Nevertheless, for the sake of consistency and clarity, we now tested all possible degraded models with the number reading task (see Table 1). This rather drastic and computationally expensive approach did indeed result in a slightly more complex model M2 than the model M1 described in our previous manuscript version not resulting into a singular fit (please see the response to the reviewer below for more details on the models). Comparison of this model to the previous one with an ANOVA indicated that M2 presents a slightly better fit of the data (despite a very similar BIC). However, this model initially selected on the base of the first number naming task’s data did lead to singularity errors when applied to the data of the second visuo-verbal matching task. This model can therefore not be used, to analyze the complete dataset. And we had to proceed to the next level of decomposition, leading back to the same model (M1) than submitted in the previous manuscript. Consequently the analysis and results remained identical to our previous submission.

It’s important to note notwithstanding the exact statistical approach and model selections, results obtained were highly consistent across the multiple analyses carried out during the revision process: from the initial ANOVA on reaction time, z-score transformed reaction time and 4 syllable long number words to the different linear mixed models where ‘60s numbers are excluded. Results also remain consistent throughout the different analysis approaches, i.e. when considering the dataset without post-error slipping, as well as when including decades as additional factor; remarkably this holds true across both tasks (see Table 2). This consistency across the several analyses convinces us about the robustness of our main findings.

We believe to have tried our best to respond to the reviewer’s concerns and those additional analyses convince us about the solidity of the main findings beyond the theoretical statistical questions of model selection. We are thus confident that the present analysis approach and the corresponding results constitute a reliable and valuable contribution to the literature. We would be very happy to share our findings on the influence of language on numerical cognition and its development with the broad readership of PLOSONE.

Kind regards and thank you for your help throughout the reviewing process,
Rémy Lachelin

Dear reviewer,

Thank you again for your points regarding the model selection. We will shortly address the minor point regarding the contrasts and extensively the major point regarding model selection.

**Regarding the contrasts**, per default the contrasts are set to sum when analyzing data with afex’s mixed: we added this information (p. 15, l. 344):

*All contrasts were set to sum.*

**Regarding model selection**, the importance of keeping the model as maximal as possible appeared clearly to us following your very helpful explanations in review n°3 (as suggested by Barr et al., 2013). The suggested theoretical maximum random effect model was consequently (MM):

(MM) RT ~ 1 + Age * Language * Number Size + (1 + Language * Number Size | Subject) + (1 + Age * Language | Item)

This model nevertheless led to singular fit. As reported in our previous answer (A1.), singular fit is due to correlations of (or approaching) +/- 1 or 0 between the random terms. As recommended by (Barr et al., 2013) in this case, we proceeded by backward selection:

“*we removed some terms from the random parts (see lines 325 to 338), aiming to preserve the most comprehensive model possible without singular fit.”*

And in the corresponding manuscript (l. 335 to 338)

*“[…] led to a singular fit (due to high correlations between the random parts of Number Size per Subject and Age per Item ) we reduced the complexity of the model by removing those problematic random terms*”

To solve singularity issues, it is recommended to either remove the problematic components or use Bayesian methods (see isSingular ?). We decided to degrade the maximal model (MM), since the use of Bayesian methods is beyond the scope of the present study. The choice of which random terms to remove was based on the variance correlation matrix (VarCor), starting with the terms with correlations of +/-1. Hence, for example the random parameter Language * Number Size | Subject was decomposed and tested separately into Language | Subject, and Number Size | Subject. Many of those models lead to singular fits again, which is not surprising given the small number of participants and trials in the data.

The reviewer is concerned about alpha inflation and suggests following the recommendations (Barr et al., 2013, p.276):

- “check for possible misspecifications or data problems”
- “outlier removal methods and to center or sumcode the predictors”
  - The data was checked for outliers with threshold RT filters, both overall and on the participant’s level (+/- 3 SD from the individual mean).
- “increase the maximum number of iterations”
  - We tried to augment the iterations and different optimizers (i.e. all_fit function), which all lead to singular fits
- “presence of a few subjects (or items) with small numbers of observations in particular cells.”
  - We checked the number of items per subject in each cell.
- “general rule of thumb: for whatever fixed effects are of critical interest, the corresponding random effects should be present in that analysis.”

At this point, we want to highlight that Barr et al. (2013)’s theoretically driven model selection method is not the sole way to proceed with model selection. How to proceed is still debated among statisticians and there are as of today no golden standards regarding model selection, with different guidelines and procedures between authors. For example:

Matuschek et al. (2017) argue that the simplification of the model must be defined *a priori*.

Bates et al. (2018) argue for “parsimonious models” instead of maximal. They argue that the model parametrization must be supported by the data since overparametrized models might be uninterpretable. The reason is that adding random terms might not add significantly explained variance.

*“However, the advice to “keep it maximal” often creates hopelessly over-specified random effects because the number of correlation parameters to estimate rises quickly with the dimension of the random-effects vectors. The information in the data may not be sufficient to support estimations of such complex models and may result in singular covariance matrices, even when the LMM is identifiable in principle. In this case, we need to replace the complex LMM specification by a more parsimonious one”*

Now, we consulted three different experts in statistics (from the LUCET group at University Luxembourg, University Potsdam, and university Marseille-Aix-Provence) who all agreed about the absence of a golden standard in the model selection process and could not point to a major issue in our model selection. Nevertheless, for the sake of completeness, and to settle the question of the robustness of our results and model, we decided to run all possible models by decomposing the interaction A*B or the random terms into A + B + A:B (see Table 1). We proceeded by systematically removing one term, one correlation, and one intercept at a time. For each formula, we retrieved the convergence and singularity errors, BIC and AIC on the number reading task’s data. This procedure indicates that most of the models lead to singular fits, but we could also find a converging model with an additional term M2 compared to the model M1^[1]^ included in our previous manuscript version.

(M2) RT ~ 1 + Age * Language * Number Size + (1 + Language + Number Size| Subject) + (1 + Language | Item)

Despite that this model M2 also converged for z-scores and 4-syllable long number words, it led to singular fits in the corresponding analyses of the verbal-visual matching task’s RT. The following most complex models without singular fits are:

(M1) RT ~ 1 + Age * Language * Number Size + (1 + Language | Subject) + (1 + Language | Item)

(M3) RT ~ 1 + Age * Language * Number Size + (1 + Number Size| Subject) + (1 + Language | Item)

Both models fitted for the number naming task’s data did not significantly differ in an ANOVA comparison. However, M1 has smaller BIC, and was therefore selected as the final model. Therefore we keep M1, as it was implemented in the previous revision, since it is the maximal possible model that fits with both datasets (please note that M2 gives the same results pattern as M1 in the number reading task, see Table 2).

More generally, the results are in line with the results of all previous analyses. Indeed the significance pattern matches across the numerous analyses we conducted at the different stages of the revision of our work: from the first submitted ANOVA with the additional analyses on z-score transformed RT, and on stimuli restricted to 4 syllables-long number words, to the corresponding linear mixed model without 60’s decades and including the supplementary analyses suggested by the reviewer considering the decades as additional factors and removal of post-error slipping trials (see Table 2).

We would like to thank again the reviewer for the very thorough and constructive comments and suggestions throughout the process.

References:

Barr, D. J., Levy, R., Scheepers, C., & Tily, H. J. (2013). Random effects structure for confirmatory hypothesis testing: Keep it maximal. *Journal of Memory and Language*, *68*(3), 255–278. https://doi.org/10.1016/j.jml.2012.11.001

Bates, D., Kliegl, R., Vasishth, S., & Baayen, H. (2018). *Parsimonious Mixed Models* (arXiv:1506.04967). arXiv. http://arxiv.org/abs/1506.04967

Matuschek, H., Kliegl, R., Vasishth, S., Baayen, H., & Bates, D. (2017). Balancing Type I error and power in linear mixed models. *Journal of Memory and Language*, *94*, 305–315. https://doi.org/10.1016/j.jml.2017.01.001

| Table 1: All degradations of the maximal model that were tested with their corresponding convergence, singularity errors; AIC and BIC. | | | | |
| --- | --- | --- | --- | --- |
| formula | Not Converge | Is Singular | AIC | BIC |
| **^1^RT ~ 1 + Age * Lan * Siz + (1 + Lan + Siz \| ID) + (1 + Age + Lan + Age:Lan \| Itm)** | 1 | 1 | 28589.8 | 28923 |
| **RT ~ 0 + Age * Lan * Siz + (1 + Lan + Siz \| ID) + (1 + Age + Lan + Age:Lan \| Itm)** | 1 | 1 | 28589.8 | 28923 |
| **RT ~ 1 + Age * Lan * Siz + (0 + Lan + Siz \| ID) + (1 + Age + Lan + Age:Lan \| Itm)** | 1 | 1 | 28589.8 | 28923 |
| **RT ~ 1 + Age * Lan * Siz + (1 + Lan + Siz \| ID) + (0 + Age + Lan + Age:Lan \| Itm)** | 1 | 1 | 28589.8 | 28923 |
| **RT ~ 1 + Age * Lan * Siz + (0 + Lan + Siz \| ID) + (0 + Age + Lan + Age:Lan \| Itm)** | 1 | 1 | 28589.8 | 28923 |
| **RT ~ 0 + Age * Lan * Siz + (1 + Lan + Siz \| ID) + (0 + Age + Lan + Age:Lan \| Itm)** | 1 | 1 | 28589.8 | 28923 |
| **RT ~ 0 + Age * Lan * Siz + (0 + Lan + Siz \| ID) + (1 + Age + Lan + Age:Lan \| Itm)** | 1 | 1 | 28589.8 | 28923 |
| **RT ~ 0 + Age * Lan * Siz + (0 + Lan + Siz \| ID) + (0 + Age + Lan + Age:Lan \| Itm)** | 1 | 1 | 28589.8 | 28923 |
| **RT ~ 1 + Age * Lan * Siz + (1 + Lan + Siz + Lan:Siz \| ID) + (1 + Age + Lan \| Itm)** | 1 | 1 | 28484.9 | 28722 |
| **RT ~ 0 + Age * Lan * Siz + (1 + Lan + Siz + Lan:Siz \| ID) + (1 + Age + Lan \| Itm)** | 1 | 1 | 28484.9 | 28722 |
| **RT ~ 1 + Age * Lan * Siz + (0 + Lan + Siz + Lan:Siz \| ID) + (1 + Age + Lan \| Itm)** | 1 | 1 | 28484.9 | 28722 |
| **RT ~ 1 + Age * Lan * Siz + (1 + Lan + Siz + Lan:Siz \| ID) + (0 + Age + Lan \| Itm)** | 1 | 1 | 28484.9 | 28722 |
| **RT ~ 1 + Age * Lan * Siz + (0 + Lan + Siz + Lan:Siz \| ID) + (0 + Age + Lan \| Itm)** | 1 | 1 | 28484.9 | 28722 |
| **RT ~ 0 + Age * Lan * Siz + (1 + Lan + Siz + Lan:Siz \| ID) + (0 + Age + Lan \| Itm)** | 1 | 1 | 28484.9 | 28722 |
| **RT ~ 0 + Age * Lan * Siz + (0 + Lan + Siz + Lan:Siz \| ID) + (1 + Age + Lan \| Itm)** | 1 | 1 | 28484.9 | 28722 |
| **RT ~ 0 + Age * Lan * Siz + (0 + Lan + Siz + Lan:Siz \| ID) + (0 + Age + Lan \| Itm)** | 1 | 1 | 28484.9 | 28722 |
| **RT ~ 1 + Age * Lan * Siz + (1 + Lan + Siz \| ID) + (1 + Age + Lan \| Itm)** | 1 | 1 | 28580.3 | 28795 |
| **RT ~ 0 + Age * Lan * Siz + (1 + Lan + Siz \| ID) + (1 + Age + Lan \| Itm)** | 1 | 1 | 28580.3 | 28795 |
| **RT ~ 1 + Age * Lan * Siz + (0 + Lan + Siz \| ID) + (1 + Age + Lan \| Itm)** | 1 | 1 | 28580.3 | 28795 |
| **RT ~ 1 + Age * Lan * Siz + (1 + Lan + Siz \| ID) + (0 + Age + Lan \| Itm)** | 1 | 1 | 28580.2 | 28795 |
| **RT ~ 1 + Age * Lan * Siz + (0 + Lan + Siz \| ID) + (0 + Age + Lan \| Itm)** | 1 | 1 | 28580.3 | 28795 |
| **RT ~ 0 + Age * Lan * Siz + (1 + Lan + Siz \| ID) + (0 + Age + Lan \| Itm)** | 1 | 1 | 28580.2 | 28795 |
| **RT ~ 0 + Age * Lan * Siz + (0 + Lan + Siz \| ID) + (1 + Age + Lan \| Itm)** | 1 | 1 | 28580.3 | 28795 |
| **RT ~ 0 + Age * Lan * Siz + (0 + Lan + Siz \| ID) + (0 + Age + Lan \| Itm)** | 1 | 1 | 28580.3 | 28795 |
| **RT ~ 1 + Age * Lan * Siz + (1 + Siz \| ID) + (1 + Age + Lan \| Itm)** | 1 | 1 | 29105.5 | 29303 |
| **RT ~ 0 + Age * Lan * Siz + (1 + Siz \| ID) + (1 + Age + Lan \| Itm)** | 1 | 1 | 29105.5 | 29303 |
| **RT ~ 1 + Age * Lan * Siz + (0 + Siz \| ID) + (1 + Age + Lan \| Itm)** | 1 | 1 | 29105.5 | 29303 |
| **RT ~ 1 + Age * Lan * Siz + (1 + Siz \| ID) + (0 + Age + Lan \| Itm)** | 1 | 1 | 29105.5 | 29303 |
| **RT ~ 1 + Age * Lan * Siz + (0 + Siz \| ID) + (0 + Age + Lan \| Itm)** | 1 | 1 | 29107.7 | 29305 |
| **RT ~ 0 + Age * Lan * Siz + (1 + Siz \| ID) + (0 + Age + Lan \| Itm)** | 1 | 1 | 29105.5 | 29303 |
| **RT ~ 0 + Age * Lan * Siz + (0 + Siz \| ID) + (1 + Age + Lan \| Itm)** | 1 | 1 | 29105.5 | 29303 |
| **RT ~ 0 + Age * Lan * Siz + (0 + Siz \| ID) + (0 + Age + Lan \| Itm)** | 1 | 1 | 29107.7 | 29305 |
| **RT ~ 1 + Age * Lan * Siz + (1 + Lan \| ID) + (1 + Age + Lan \| Itm)** | 1 | 1 | 28599.3 | 28797 |
| **RT ~ 0 + Age * Lan * Siz + (1 + Lan \| ID) + (1 + Age + Lan \| Itm)** | 1 | 1 | 28599.3 | 28797 |
| **RT ~ 1 + Age * Lan * Siz + (0 + Lan \| ID) + (1 + Age + Lan \| Itm)** | 1 | 1 | 28599.3 | 28797 |
| **RT ~ 1 + Age * Lan * Siz + (1 + Lan \| ID) + (0 + Age + Lan \| Itm)** | 1 | 1 | 28599.3 | 28797 |
| **RT ~ 1 + Age * Lan * Siz + (0 + Lan \| ID) + (0 + Age + Lan \| Itm)** | 1 | 1 | 28603.1 | 28800 |
| **RT ~ 0 + Age * Lan * Siz + (1 + Lan \| ID) + (0 + Age + Lan \| Itm)** | 1 | 1 | 28599.3 | 28797 |
| **RT ~ 0 + Age * Lan * Siz + (0 + Lan \| ID) + (1 + Age + Lan \| Itm)** | 1 | 1 | 28599.3 | 28797 |
| **RT ~ 0 + Age * Lan * Siz + (0 + Lan \| ID) + (0 + Age + Lan \| Itm)** | 1 | 1 | 28603.1 | 28800 |
| **^2^RT ~ 1 + Age * Lan * Siz + (1 + Lan + Siz \| ID) + (1 + Lan \| Itm)** | 0 | 0 | 28576.5 | 28723 |
| **RT ~ 0 + Age * Lan * Siz + (1 + Lan + Siz \| ID) + (1 + Lan \| Itm)** | 0 | 0 | 28576.5 | 28723 |
| **RT ~ 1 + Age * Lan * Siz + (0 + Lan + Siz \| ID) + (1 + Lan \| Itm)** | 0 | 0 | 28576.5 | 28723 |
| **RT ~ 1 + Age * Lan * Siz + (1 + Lan + Siz \| ID) + (0 + Lan \| Itm)** | 0 | 0 | 28576.5 | 28723 |
| **RT ~ 1 + Age * Lan * Siz + (0 + Lan + Siz \| ID) + (0 + Lan \| Itm)** | 0 | 0 | 28576.5 | 28723 |
| **RT ~ 0 + Age * Lan * Siz + (1 + Lan + Siz \| ID) + (0 + Lan \| Itm)** | 1 | 0 | 28576.5 | 28723 |
| **RT ~ 0 + Age * Lan * Siz + (0 + Lan + Siz \| ID) + (1 + Lan \| Itm)** | 0 | 0 | 28576.5 | 28723 |
| **RT ~ 0 + Age * Lan * Siz + (0 + Lan + Siz \| ID) + (0 + Lan \| Itm)** | 0 | 0 | 28576.5 | 28723 |
| **RT ~ 1 + Age * Lan * Siz + (1 + Lan + Siz \| ID) + (1 + Age \| Itm)** | 1 | 1 | 28600 | 28786 |
| **RT ~ 0 + Age * Lan * Siz + (1 + Lan + Siz \| ID) + (1 + Age \| Itm)** | 1 | 1 | 28600 | 28786 |
| **RT ~ 1 + Age * Lan * Siz + (0 + Lan + Siz \| ID) + (1 + Age \| Itm)** | 1 | 1 | 28600 | 28786 |
| **RT ~ 1 + Age * Lan * Siz + (1 + Lan + Siz \| ID) + (0 + Age \| Itm)** | 1 | 1 | 28600 | 28786 |
| **RT ~ 1 + Age * Lan * Siz + (0 + Lan + Siz \| ID) + (0 + Age \| Itm)** | 1 | 1 | 28607.4 | 28794 |
| **RT ~ 0 + Age * Lan * Siz + (1 + Lan + Siz \| ID) + (0 + Age \| Itm)** | 1 | 1 | 28600 | 28786 |
| **RT ~ 1 + Age * Lan * Siz + (0 + Lan + Siz \| ID) + (1 + Age \| Itm)** | 1 | 1 | 28600 | 28786 |
| **RT ~ 0 + Age * Lan * Siz + (0 + Lan + Siz \| ID) + (0 + Age \| Itm)** | 1 | 1 | 28607.4 | 28794 |
| **^3^RT ~ 1 + Age * Lan * Siz + (1 + Siz \| ID) + (1 + Lan \| Itm)** | 0 | 0 | 29103.8 | 29233 |
| **RT ~ 0 + Age * Lan * Siz + (1 + Siz \| ID) + (1 + Lan \| Itm)** | 0 | 0 | 29103.8 | 29233 |
| **RT ~ 1 + Age * Lan * Siz + (0 + Siz \| ID) + (1 + Lan \| Itm)** | 0 | 0 | 29103.8 | 29233 |
| **RT ~ 1 + Age * Lan * Siz + (1 + Siz \| ID) + (0 + Lan \| Itm)** | 0 | 0 | 29103.8 | 29233 |
| **RT ~ 1 + Age * Lan * Siz + (0 + Siz \| ID) + (0 + Lan \| Itm)** | 0 | 0 | 29103.8 | 29233 |
| **RT ~ 0 + Age * Lan * Siz + (1 + Siz \| ID) + (0 + Lan \| Itm)** | 0 | 0 | 29103.8 | 29233 |
| **RT ~ 0 + Age * Lan * Siz + (0 + Siz \| ID) + (1 + Lan \| Itm)** | 0 | 0 | 29103.8 | 29233 |
| **RT ~ 0 + Age * Lan * Siz + (0 + Siz \| ID) + (0 + Lan \| Itm)** | 0 | 0 | 29103.8 | 29233 |
| **RT ~ 1 + Age * Lan * Siz + (1 + Siz \| ID) + (1 + Age \| Itm)** | 1 | 1 | 29124.3 | 29293 |
| **RT ~ 0 + Age * Lan * Siz + (1 + Siz \| ID) + (1 + Age \| Itm)** | 1 | 1 | 29124.3 | 29293 |
| **RT ~ 1 + Age * Lan * Siz + (0 + Siz \| ID) + (1 + Age \| Itm)** | 1 | 1 | 29124.3 | 29293 |
| **RT ~ 1 + Age * Lan * Siz + (1 + Siz \| ID) + (0 + Age \| Itm)** | 1 | 1 | 29124.3 | 29293 |
| **RT ~ 1 + Age * Lan * Siz + (0 + Siz \| ID) + (0 + Age \| Itm)** | 1 | 1 | 29124.3 | 29293 |
| **RT ~ 0 + Age * Lan * Siz + (1 + Siz \| ID) + (0 + Age \| Itm)** | 1 | 1 | 29124.3 | 29293 |
| **RT ~ 0 + Age * Lan * Siz + (0 + Siz \| ID) + (1 + Age \| Itm)** | 1 | 1 | 29124.3 | 29293 |
| **RT ~ 0 + Age * Lan * Siz + (0 + Siz \| ID) + (0 + Age \| Itm)** | 1 | 1 | 29136.6 | 29306 |
| **RT ~ 1 + Age * Lan * Siz + (1 + Lan \| ID) + (1 + Age \| Itm)** | 1 | 1 | 28619 | 28788 |
| **RT ~ 0 + Age * Lan * Siz + (1 + Lan \| ID) + (1 + Age \| Itm)** | 1 | 1 | 28619 | 28788 |
| **RT ~ 1 + Age * Lan * Siz + (0 + Lan \| ID) + (1 + Age \| Itm)** | 1 | 1 | 28619 | 28788 |
| **RT ~ 1 + Age * Lan * Siz + (1 + Lan \| ID) + (0 + Age \| Itm)** | 1 | 1 | 28619 | 28788 |
| **RT ~ 1 + Age * Lan * Siz + (0 + Lan \| ID) + (0 + Age \| Itm)** | 1 | 1 | 28619 | 28788 |
| **RT ~ 0 + Age * Lan * Siz + (1 + Lan \| ID) + (0 + Age \| Itm)** | 1 | 1 | 28619 | 28788 |
| **RT ~ 0 + Age * Lan * Siz + (0 + Lan \| ID) + (1 + Age \| Itm)** | 1 | 1 | 28619 | 28788 |
| **RT ~ 0 + Age * Lan * Siz + (0 + Lan \| ID) + (0 + Age \| Itm)** | 1 | 1 | 28619 | 28788 |
| **^4^RT ~ 1 + Age * Lan * Siz + (1 + Lan \| ID) + (1 + Lan \| Itm)** | 0 | 0 | 28594.1 | 28724 |
| **RT ~ 0 + Age * Lan * Siz + (1 + Lan \| ID) + (1 + Lan \| Itm)** | 0 | 0 | 28594.1 | 28724 |
| **RT ~ 1 + Age * Lan * Siz + (0 + Lan \| ID) + (1 + Lan \| Itm)** | 0 | 0 | 28594.1 | 28724 |
| **RT ~ 1 + Age * Lan * Siz + (1 + Lan \| ID) + (0 + Lan \| Itm)** | 0 | 0 | 28594.1 | 28724 |
| **RT ~ 1 + Age * Lan * Siz + (0 + Lan \| ID) + (0 + Lan \| Itm)** | 0 | 0 | 28594.1 | 28724 |
| **RT ~ 0 + Age * Lan * Siz + (1 + Lan \| ID) + (0 + Lan \| Itm)** | 0 | 0 | 28594.1 | 28724 |
| **RT ~ 0 + Age * Lan * Siz + (0 + Lan \| ID) + (1 + Lan \| Itm)** | 0 | 0 | 28594.1 | 28724 |
| **RT ~ 0 + Age * Lan * Siz + (0 + Lan \| ID) + (0 + Lan \| Itm)** | 0 | 0 | 28594.1 | 28724 |
| **RT ~ 1 + Age * Lan * Siz + (1 + Lan + Siz \|\| ID) + (1 + Age + Lan + Age:Lan \| Itm)** | 1 | 1 | 28603 | 28942 |
| **RT ~ 0 + Age * Lan * Siz + (1 + Lan + Siz \|\| ID) + (1 + Age + Lan + Age:Lan \| Itm)** | 1 | 1 | 28603 | 28942 |
| **RT ~ 1 + Age * Lan * Siz + (0 + Lan + Siz \|\| ID) + (1 + Age + Lan + Age:Lan \| Itm)** | 1 | 1 | 28601 | 28934 |
| **RT ~ 1 + Age * Lan * Siz + (1 + Lan + Siz \|\| ID) + (0 + Age + Lan + Age:Lan \| Itm)** | 1 | 1 | 28612 | 28950 |
| **RT ~ 1 + Age * Lan * Siz + (0 + Lan + Siz \|\| ID) + (0 + Age + Lan + Age:Lan \| Itm)** | 1 | 1 | 28601 | 28934 |
| **RT ~ 0 + Age * Lan * Siz + (1 + Lan + Siz \|\| ID) + (0 + Age + Lan + Age:Lan \| Itm)** | 1 | 1 | 28612 | 28950 |
| **RT ~ 0 + Age * Lan * Siz + (0 + Lan + Siz \|\| ID) + (1 + Age + Lan + Age:Lan \| Itm)** | 1 | 1 | 28601 | 28934 |
| **RT ~ 0 + Age * Lan * Siz + (0 + Lan + Siz \|\| ID) + (0 + Age + Lan + Age:Lan \| Itm)** | 1 | 1 | 28601 | 28934 |
| **RT ~ 1 + Age * Lan * Siz + (1 + Lan + Siz + Lan:Siz \|\| ID) + (1 + Age + Lan \| Itm)** | 1 | 1 | 28499 | 28775 |
| **RT ~ 0 + Age * Lan * Siz + (1 + Lan + Siz + Lan:Siz \|\| ID) + (1 + Age + Lan \| Itm)** | 1 | 1 | 28499 | 28775 |
| **RT ~ 1 + Age * Lan * Siz + (0 + Lan + Siz + Lan:Siz \|\| ID) + (1 + Age + Lan \| Itm)** | 1 | 1 | 28497 | 28768 |
| **RT ~ 1 + Age * Lan * Siz + (1 + Lan + Siz + Lan:Siz \|\| ID) + (0 + Age + Lan \| Itm)** | 1 | 1 | 28499 | 28775 |
| **RT ~ 1 + Age * Lan * Siz + (0 + Lan + Siz + Lan:Siz \|\| ID) + (0 + Age + Lan \| Itm)** | 1 | 1 | 28497 | 28768 |
| **RT ~ 0 + Age * Lan * Siz + (1 + Lan + Siz + Lan:Siz \|\| ID) + (0 + Age + Lan \| Itm)** | 1 | 1 | 28499 | 28775 |
| **RT ~ 0 + Age * Lan * Siz + (0 + Lan + Siz + Lan:Siz \|\| ID) + (1 + Age + Lan \| Itm)** | 1 | 1 | 28497 | 28768 |
| **RT ~ 0 + Age * Lan * Siz + (0 + Lan + Siz + Lan:Siz \|\| ID) + (0 + Age + Lan \| Itm)** | 1 | 1 | 28497 | 28768 |
| **RT ~ 1 + Age * Lan * Siz + (1 + Lan + Siz \|\| ID) + (1 + Age + Lan \| Itm)** | 1 | 1 | 28594 | 28814 |
| **RT ~ 0 + Age * Lan * Siz + (1 + Lan + Siz \|\| ID) + (1 + Age + Lan \| Itm)** | 1 | 1 | 28594 | 28814 |
| **RT ~ 1 + Age * Lan * Siz + (0 + Lan + Siz \|\| ID) + (1 + Age + Lan \| Itm)** | 1 | 1 | 28592 | 28807 |
| **RT ~ 1 + Age * Lan * Siz + (1 + Lan + Siz \|\| ID) + (0 + Age + Lan \| Itm)** | 1 | 1 | 28603 | 28823 |
| **RT ~ 1 + Age * Lan * Siz + (0 + Lan + Siz \|\| ID) + (0 + Age + Lan \| Itm)** | 1 | 1 | 28592 | 28807 |
| **RT ~ 0 + Age * Lan * Siz + (1 + Lan + Siz \|\| ID) + (0 + Age + Lan \| Itm)** | 1 | 1 | 28603 | 28823 |
| **RT ~ 0 + Age * Lan * Siz + (0 + Lan + Siz \|\| ID) + (1 + Age + Lan \| Itm)** | 1 | 0 | 28592 | 28807 |
| **RT ~ 0 + Age * Lan * Siz + (0 + Lan + Siz \|\| ID) + (0 + Age + Lan \| Itm)** | 1 | 1 | 28592 | 28807 |
| **RT ~ 1 + Age * Lan * Siz + (1 + Siz \|\| ID) + (1 + Age + Lan \| Itm)** | 1 | 1 | 29108 | 29311 |
| **RT ~ 0 + Age * Lan * Siz + (1 + Siz \|\| ID) + (1 + Age + Lan \| Itm)** | 1 | 1 | 29108 | 29311 |
| **RT ~ 1 + Age * Lan * Siz + (0 + Siz \|\| ID) + (1 + Age + Lan \| Itm)** | 1 | 1 | 29106 | 29303 |
| **RT ~ 1 + Age * Lan * Siz + (1 + Siz \|\| ID) + (0 + Age + Lan \| Itm)** | 1 | 1 | 29108 | 29311 |
| **RT ~ 1 + Age * Lan * Siz + (0 + Siz \|\| ID) + (0 + Age + Lan \| Itm)** | 1 | 1 | 29108 | 29305 |
| **RT ~ 0 + Age * Lan * Siz + (1 + Siz \|\| ID) + (0 + Age + Lan \| Itm)** | 1 | 1 | 29108 | 29311 |
| **RT ~ 0 + Age * Lan * Siz + (0 + Siz \|\| ID) + (1 + Age + Lan \| Itm)** | 1 | 1 | 29106 | 29303 |
| **RT ~ 0 + Age * Lan * Siz + (0 + Siz \|\| ID) + (0 + Age + Lan \| Itm)** | 1 | 1 | 29108 | 29305 |
| **RT ~ 1 + Age * Lan * Siz + (1 + Lan \|\| ID) + (1 + Age + Lan \| Itm)** | 1 | 1 | 28601 | 28804 |
| **RT ~ 0 + Age * Lan * Siz + (1 + Lan \|\| ID) + (1 + Age + Lan \| Itm)** | 1 | 1 | 28601 | 28804 |
| **RT ~ 1 + Age * Lan * Siz + (0 + Lan \|\| ID) + (1 + Age + Lan \| Itm)** | 1 | 1 | 28599 | 28797 |
| **RT ~ 1 + Age * Lan * Siz + (1 + Lan \|\| ID) + (0 + Age + Lan \| Itm)** | 1 | 1 | 28601 | 28804 |
| **RT ~ 1 + Age * Lan * Siz + (0 + Lan \|\| ID) + (0 + Age + Lan \| Itm)** | 1 | 1 | 28603 | 28800 |
| **RT ~ 0 + Age * Lan * Siz + (1 + Lan \|\| ID) + (0 + Age + Lan \| Itm)** | 1 | 1 | 28601 | 28804 |
| **RT ~ 0 + Age * Lan * Siz + (0 + Lan \|\| ID) + (1 + Age + Lan \| Itm)** | 1 | 1 | 28599 | 28797 |
| **RT ~ 0 + Age * Lan * Siz + (0 + Lan \|\| ID) + (0 + Age + Lan \| Itm)** | 1 | 1 | 28603 | 28800 |
| **RT ~ 1 + Age * Lan * Siz + (1 + Lan + Siz \|\| ID) + (1 + Lan \| Itm)** | 1 | 0 | 28591 | 28743 |
| **RT ~ 0 + Age * Lan * Siz + (1 + Lan + Siz \|\| ID) + (1 + Lan \| Itm)** | 1 | 0 | 28591 | 28743 |
| **RT ~ 1 + Age * Lan * Siz + (0 + Lan + Siz \|\| ID) + (1 + Lan \| Itm)** | 0 | 0 | 28589 | 28735 |
| **RT ~ 1 + Age * Lan * Siz + (1 + Lan + Siz \|\| ID) + (0 + Lan \| Itm)** | 1 | 0 | 28591 | 28743 |
| **RT ~ 1 + Age * Lan * Siz + (0 + Lan + Siz \|\| ID) + (0 + Lan \| Itm)** | 1 | 0 | 28589 | 28735 |
| **RT ~ 0 + Age * Lan * Siz + (1 + Lan + Siz \|\| ID) + (0 + Lan \| Itm)** | 1 | 0 | 28591 | 28743 |
| **RT ~ 0 + Age * Lan * Siz + (0 + Lan + Siz \|\| ID) + (1 + Lan \| Itm)** | 1 | 0 | 28589 | 28735 |
| **RT ~ 0 + Age * Lan * Siz + (0 + Lan + Siz \|\| ID) + (0 + Lan \| Itm)** | 1 | 0 | 28589 | 28735 |
| **RT ~ 1 + Age * Lan * Siz + (1 + Lan + Siz \|\| ID) + (1 + Age \| Itm)** | 1 | 1 | 28615 | 28807 |
| **RT ~ 0 + Age * Lan * Siz + (1 + Lan + Siz \|\| ID) + (1 + Age \| Itm)** | 1 | 1 | 28615 | 28807 |
| **RT ~ 1 + Age * Lan * Siz + (0 + Lan + Siz \|\| ID) + (1 + Age \| Itm)** | 1 | 1 | 28613 | 28799 |
| **RT ~ 1 + Age * Lan * Siz + (1 + Lan + Siz \|\| ID) + (0 + Age \| Itm)** | 1 | 1 | 28615 | 28807 |
| **RT ~ 1 + Age * Lan * Siz + (0 + Lan + Siz \|\| ID) + (0 + Age \| Itm)** | 1 | 1 | 28620 | 28806 |
| **RT ~ 0 + Age * Lan * Siz + (1 + Lan + Siz \|\| ID) + (0 + Age \| Itm)** | 1 | 1 | 28615 | 28807 |
| **RT ~ 1 + Age * Lan * Siz + (0 + Lan + Siz \|\| ID) + (1 + Age \| Itm)** | 1 | 1 | 28613 | 28799 |
| **RT ~ 0 + Age * Lan * Siz + (0 + Lan + Siz \|\| ID) + (0 + Age \| Itm)** | 1 | 1 | 28620 | 28806 |
| **RT ~ 1 + Age * Lan * Siz + (1 + Siz \|\| ID) + (1 + Lan \| Itm)** | 1 | 0 | 29106 | 29241 |
| **RT ~ 0 + Age * Lan * Siz + (1 + Siz \|\| ID) + (1 + Lan \| Itm)** | 0 | 0 | 29106 | 29241 |
| **RT ~ 1 + Age * Lan * Siz + (0 + Siz \|\| ID) + (1 + Lan \| Itm)** | 0 | 0 | 29104 | 29233 |
| **RT ~ 1 + Age * Lan * Siz + (1 + Siz \|\| ID) + (0 + Lan \| Itm)** | 0 | 0 | 29106 | 29241 |
| **RT ~ 1 + Age * Lan * Siz + (0 + Siz \|\| ID) + (0 + Lan \| Itm)** | 0 | 0 | 29104 | 29233 |
| **RT ~ 0 + Age * Lan * Siz + (1 + Siz \|\| ID) + (0 + Lan \| Itm)** | 0 | 0 | 29106 | 29241 |
| **RT ~ 0 + Age * Lan * Siz + (0 + Siz \|\| ID) + (1 + Lan \| Itm)** | 0 | 0 | 29104 | 29233 |
| **RT ~ 0 + Age * Lan * Siz + (0 + Siz \|\| ID) + (0 + Lan \| Itm)** | 0 | 0 | 29104 | 29233 |
| **RT ~ 1 + Age * Lan * Siz + (1 + Siz \|\| ID) + (1 + Age \| Itm)** | 1 | 1 | 29126 | 29301 |
| **RT ~ 0 + Age * Lan * Siz + (1 + Siz \|\| ID) + (1 + Age \| Itm)** | 1 | 1 | 29126 | 29301 |
| **RT ~ 1 + Age * Lan * Siz + (0 + Siz \|\| ID) + (1 + Age \| Itm)** | 1 | 1 | 29124 | 29293 |
| **RT ~ 1 + Age * Lan * Siz + (1 + Siz \|\| ID) + (0 + Age \| Itm)** | 1 | 1 | 29126 | 29301 |
| **RT ~ 1 + Age * Lan * Siz + (0 + Siz \|\| ID) + (0 + Age \| Itm)** | 1 | 1 | 29124 | 29293 |
| **RT ~ 0 + Age * Lan * Siz + (1 + Siz \|\| ID) + (0 + Age \| Itm)** | 1 | 1 | 29126 | 29301 |
| **RT ~ 0 + Age * Lan * Siz + (0 + Siz \|\| ID) + (1 + Age \| Itm)** | 1 | 1 | 29124 | 29293 |
| **RT ~ 0 + Age * Lan * Siz + (0 + Siz \|\| ID) + (0 + Age \| Itm)** | 1 | 1 | 29137 | 29306 |
| **RT ~ 1 + Age * Lan * Siz + (1 + Lan \|\| ID) + (1 + Age \| Itm)** | 1 | 1 | 28627 | 28802 |
| **RT ~ 0 + Age * Lan * Siz + (1 + Lan \|\| ID) + (1 + Age \| Itm)** | 1 | 1 | 28627 | 28802 |
| **RT ~ 1 + Age * Lan * Siz + (0 + Lan \|\| ID) + (1 + Age \| Itm)** | 1 | 1 | 28619 | 28788 |
| **RT ~ 1 + Age * Lan * Siz + (1 + Lan \|\| ID) + (0 + Age \| Itm)** | 1 | 1 | 28621 | 28796 |
| **RT ~ 1 + Age * Lan * Siz + (0 + Lan \|\| ID) + (0 + Age \| Itm)** | 1 | 1 | 28619 | 28788 |
| **RT ~ 0 + Age * Lan * Siz + (1 + Lan \|\| ID) + (0 + Age \| Itm)** | 1 | 1 | 28621 | 28796 |
| **RT ~ 0 + Age * Lan * Siz + (0 + Lan \|\| ID) + (1 + Age \| Itm)** | 1 | 1 | 28619 | 28788 |
| **RT ~ 0 + Age * Lan * Siz + (0 + Lan \|\| ID) + (0 + Age \| Itm)** | 1 | 1 | 28619 | 28788 |
| **RT ~ 1 + Age * Lan * Siz + (1 + Lan \|\| ID) + (1 + Lan \| Itm)** | 1 | 1 | 28623 | 28758 |
| **RT ~ 0 + Age * Lan * Siz + (1 + Lan \|\| ID) + (1 + Lan \| Itm)** | 1 | 1 | 28623 | 28758 |
| **RT ~ 1 + Age * Lan * Siz + (0 + Lan \|\| ID) + (1 + Lan \| Itm)** | 0 | 0 | 28594 | 28724 |
| **RT ~ 1 + Age * Lan * Siz + (1 + Lan \|\| ID) + (0 + Lan \| Itm)** | 0 | 0 | 28596 | 28731 |
| **RT ~ 1 + Age * Lan * Siz + (0 + Lan \|\| ID) + (0 + Lan \| Itm)** | 0 | 0 | 28594 | 28724 |
| **RT ~ 0 + Age * Lan * Siz + (1 + Lan \|\| ID) + (0 + Lan \| Itm)** | 1 | 0 | 28596 | 28731 |
| **RT ~ 0 + Age * Lan * Siz + (0 + Lan \|\| ID) + (1 + Lan \| Itm)** | 0 | 0 | 28594 | 28724 |
| **RT ~ 0 + Age * Lan * Siz + (0 + Lan \|\| ID) + (0 + Lan \| Itm)** | 0 | 0 | 28594 | 28724 |
| **RT ~ 1 + Age * Lan * Siz + (1 + Lan + Siz \| ID) + (1 + Age + Lan + Age:Lan \|\| Itm)** | 1 | 1 | 28618 | 29030 |
| **RT ~ 0 + Age * Lan * Siz + (1 + Lan + Siz \| ID) + (1 + Age + Lan + Age:Lan \|\| Itm)** | 1 | 1 | 28618 | 29030 |
| **RT ~ 1 + Age * Lan * Siz + (0 + Lan + Siz \| ID) + (1 + Age + Lan + Age:Lan \|\| Itm)** | 1 | 1 | 28618 | 29030 |
| **RT ~ 1 + Age * Lan * Siz + (1 + Lan + Siz \| ID) + (0 + Age + Lan + Age:Lan \|\| Itm)** | 1 | 1 | 28616 | 29022 |
| **RT ~ 1 + Age * Lan * Siz + (0 + Lan + Siz \| ID) + (0 + Age + Lan + Age:Lan \|\| Itm)** | 1 | 1 | 28616 | 29022 |
| **RT ~ 0 + Age * Lan * Siz + (1 + Lan + Siz \| ID) + (0 + Age + Lan + Age:Lan \|\| Itm)** | 1 | 1 | 28616 | 29022 |
| **RT ~ 0 + Age * Lan * Siz + (0 + Lan + Siz \| ID) + (1 + Age + Lan + Age:Lan \|\| Itm)** | 1 | 1 | 28618 | 29030 |
| **RT ~ 0 + Age * Lan * Siz + (0 + Lan + Siz \| ID) + (0 + Age + Lan + Age:Lan \|\| Itm)** | 1 | 1 | 28616 | 29022 |
| **RT ~ 1 + Age * Lan * Siz + (1 + Lan + Siz + Lan:Siz \| ID) + (1 + Age + Lan \|\| Itm)** | 1 | 1 | 28492 | 28723 |
| **RT ~ 0 + Age * Lan * Siz + (1 + Lan + Siz + Lan:Siz \| ID) + (1 + Age + Lan \|\| Itm)** | 1 | 1 | 28492 | 28723 |
| **RT ~ 1 + Age * Lan * Siz + (0 + Lan + Siz + Lan:Siz \| ID) + (1 + Age + Lan \|\| Itm)** | 1 | 1 | 28492 | 28723 |
| **RT ~ 1 + Age * Lan * Siz + (1 + Lan + Siz + Lan:Siz \| ID) + (0 + Age + Lan \|\| Itm)** | 1 | 1 | 28490 | 28716 |
| **RT ~ 1 + Age * Lan * Siz + (0 + Lan + Siz + Lan:Siz \| ID) + (0 + Age + Lan \|\| Itm)** | 1 | 1 | 28490 | 28716 |
| **RT ~ 0 + Age * Lan * Siz + (1 + Lan + Siz + Lan:Siz \| ID) + (0 + Age + Lan \|\| Itm)** | 1 | 1 | 28490 | 28716 |
| **RT ~ 0 + Age * Lan * Siz + (0 + Lan + Siz + Lan:Siz \| ID) + (1 + Age + Lan \|\| Itm)** | 1 | 1 | 28492 | 28723 |
| **RT ~ 0 + Age * Lan * Siz + (0 + Lan + Siz + Lan:Siz \| ID) + (0 + Age + Lan \|\| Itm)** | 1 | 1 | 28490 | 28716 |
| **RT ~ 1 + Age * Lan * Siz + (1 + Lan + Siz \| ID) + (1 + Age + Lan \|\| Itm)** | 1 | 1 | 28586 | 28794 |
| **RT ~ 0 + Age * Lan * Siz + (1 + Lan + Siz \| ID) + (1 + Age + Lan \|\| Itm)** | 1 | 1 | 28586 | 28794 |
| **RT ~ 1 + Age * Lan * Siz + (0 + Lan + Siz \| ID) + (1 + Age + Lan \|\| Itm)** | 1 | 1 | 28586 | 28794 |
| **RT ~ 1 + Age * Lan * Siz + (1 + Lan + Siz \| ID) + (0 + Age + Lan \|\| Itm)** | 1 | 1 | 28589 | 28792 |
| **RT ~ 1 + Age * Lan * Siz + (0 + Lan + Siz \| ID) + (0 + Age + Lan \|\| Itm)** | 1 | 1 | 28584 | 28787 |
| **RT ~ 0 + Age * Lan * Siz + (1 + Lan + Siz \| ID) + (0 + Age + Lan \|\| Itm)** | 1 | 1 | 28589 | 28792 |
| **RT ~ 0 + Age * Lan * Siz + (0 + Lan + Siz \| ID) + (1 + Age + Lan \|\| Itm)** | 1 | 1 | 28586 | 28794 |
| **RT ~ 0 + Age * Lan * Siz + (0 + Lan + Siz \| ID) + (0 + Age + Lan \|\| Itm)** | 1 | 1 | 28584 | 28787 |
| **RT ~ 1 + Age * Lan * Siz + (1 + Siz \| ID) + (1 + Age + Lan \|\| Itm)** | 1 | 1 | 29115 | 29307 |
| **RT ~ 0 + Age * Lan * Siz + (1 + Siz \| ID) + (1 + Age + Lan \|\| Itm)** | 1 | 1 | 29115 | 29307 |
| **RT ~ 1 + Age * Lan * Siz + (0 + Siz \| ID) + (1 + Age + Lan \|\| Itm)** | 1 | 1 | 29116 | 29308 |
| **RT ~ 1 + Age * Lan * Siz + (1 + Siz \| ID) + (0 + Age + Lan \|\| Itm)** | 1 | 1 | 29113 | 29300 |
| **RT ~ 1 + Age * Lan * Siz + (0 + Siz \| ID) + (0 + Age + Lan \|\| Itm)** | 1 | 1 | 29113 | 29300 |
| **RT ~ 0 + Age * Lan * Siz + (1 + Siz \| ID) + (0 + Age + Lan \|\| Itm)** | 1 | 1 | 29113 | 29300 |
| **RT ~ 0 + Age * Lan * Siz + (0 + Siz \| ID) + (1 + Age + Lan \|\| Itm)** | 1 | 1 | 29116 | 29308 |
| **RT ~ 0 + Age * Lan * Siz + (0 + Siz \| ID) + (0 + Age + Lan \|\| Itm)** | 1 | 1 | 29113 | 29300 |
| **RT ~ 1 + Age * Lan * Siz + (1 + Lan \| ID) + (1 + Age + Lan \|\| Itm)** | 1 | 1 | 28610 | 28802 |
| **RT ~ 0 + Age * Lan * Siz + (1 + Lan \| ID) + (1 + Age + Lan \|\| Itm)** | 1 | 1 | 28610 | 28802 |
| **RT ~ 1 + Age * Lan * Siz + (0 + Lan \| ID) + (1 + Age + Lan \|\| Itm)** | 1 | 1 | 28612 | 28804 |
| **RT ~ 1 + Age * Lan * Siz + (1 + Lan \| ID) + (0 + Age + Lan \|\| Itm)** | 1 | 1 | 28603 | 28789 |
| **RT ~ 1 + Age * Lan * Siz + (0 + Lan \| ID) + (0 + Age + Lan \|\| Itm)** | 1 | 1 | 28603 | 28789 |
| **RT ~ 0 + Age * Lan * Siz + (1 + Lan \| ID) + (0 + Age + Lan \|\| Itm)** | 1 | 1 | 28603 | 28789 |
| **RT ~ 0 + Age * Lan * Siz + (0 + Lan \| ID) + (1 + Age + Lan \|\| Itm)** | 1 | 1 | 28612 | 28804 |
| **RT ~ 0 + Age * Lan * Siz + (0 + Lan \| ID) + (0 + Age + Lan \|\| Itm)** | 1 | 1 | 28603 | 28789 |
| **RT ~ 1 + Age * Lan * Siz + (1 + Lan + Siz \| ID) + (1 + Lan \|\| Itm)** | 1 | 0 | 28578 | 28731 |
| **RT ~ 0 + Age * Lan * Siz + (1 + Lan + Siz \| ID) + (1 + Lan \|\| Itm)** | 1 | 0 | 28578 | 28731 |
| **RT ~ 1 + Age * Lan * Siz + (0 + Lan + Siz \| ID) + (1 + Lan \|\| Itm)** | 1 | 0 | 28578 | 28731 |
| **RT ~ 1 + Age * Lan * Siz + (1 + Lan + Siz \| ID) + (0 + Lan \|\| Itm)** | 0 | 0 | 28576 | 28723 |
| **RT ~ 1 + Age * Lan * Siz + (0 + Lan + Siz \| ID) + (0 + Lan \|\| Itm)** | 0 | 0 | 28576 | 28723 |
| **RT ~ 0 + Age * Lan * Siz + (1 + Lan + Siz \| ID) + (0 + Lan \|\| Itm)** | 1 | 0 | 28576 | 28723 |
| **RT ~ 0 + Age * Lan * Siz + (0 + Lan + Siz \| ID) + (1 + Lan \|\| Itm)** | 1 | 0 | 28578 | 28731 |
| **RT ~ 0 + Age * Lan * Siz + (0 + Lan + Siz \| ID) + (0 + Lan \|\| Itm)** | 0 | 0 | 28576 | 28723 |
| **RT ~ 1 + Age * Lan * Siz + (1 + Lan + Siz \| ID) + (1 + Age \|\| Itm)** | 1 | 1 | 28602 | 28794 |
| **RT ~ 0 + Age * Lan * Siz + (1 + Lan + Siz \| ID) + (1 + Age \|\| Itm)** | 1 | 1 | 28602 | 28794 |
| **RT ~ 1 + Age * Lan * Siz + (0 + Lan + Siz \| ID) + (1 + Age \|\| Itm)** | 1 | 1 | 28602 | 28794 |
| **RT ~ 1 + Age * Lan * Siz + (1 + Lan + Siz \| ID) + (0 + Age \|\| Itm)** | 1 | 1 | 28600 | 28786 |
| **RT ~ 1 + Age * Lan * Siz + (0 + Lan + Siz \| ID) + (0 + Age \|\| Itm)** | 1 | 1 | 28607 | 28794 |
| **RT ~ 0 + Age * Lan * Siz + (1 + Lan + Siz \| ID) + (0 + Age \|\| Itm)** | 1 | 1 | 28600 | 28786 |
| **RT ~ 1 + Age * Lan * Siz + (0 + Lan + Siz \| ID) + (1 + Age \|\| Itm)** | 1 | 1 | 28602 | 28794 |
| **RT ~ 0 + Age * Lan * Siz + (0 + Lan + Siz \| ID) + (0 + Age \|\| Itm)** | 1 | 1 | 28607 | 28794 |
| **RT ~ 1 + Age * Lan * Siz + (1 + Siz \| ID) + (1 + Lan \|\| Itm)** | 1 | 0 | 29106 | 29241 |
| **RT ~ 0 + Age * Lan * Siz + (1 + Siz \| ID) + (1 + Lan \|\| Itm)** | 0 | 0 | 29106 | 29241 |
| **RT ~ 1 + Age * Lan * Siz + (0 + Siz \| ID) + (1 + Lan \|\| Itm)** | 0 | 0 | 29106 | 29241 |
| **RT ~ 1 + Age * Lan * Siz + (1 + Siz \| ID) + (0 + Lan \|\| Itm)** | 0 | 0 | 29104 | 29233 |
| **RT ~ 1 + Age * Lan * Siz + (0 + Siz \| ID) + (0 + Lan \|\| Itm)** | 0 | 0 | 29104 | 29233 |
| **RT ~ 0 + Age * Lan * Siz + (1 + Siz \| ID) + (0 + Lan \|\| Itm)** | 0 | 0 | 29104 | 29233 |
| **RT ~ 0 + Age * Lan * Siz + (0 + Siz \| ID) + (1 + Lan \|\| Itm)** | 1 | 0 | 29106 | 29241 |
| **RT ~ 0 + Age * Lan * Siz + (0 + Siz \| ID) + (0 + Lan \|\| Itm)** | 0 | 0 | 29104 | 29233 |
| **RT ~ 1 + Age * Lan * Siz + (1 + Siz \| ID) + (1 + Age \|\| Itm)** | 1 | 1 | 29126 | 29301 |
| **RT ~ 0 + Age * Lan * Siz + (1 + Siz \| ID) + (1 + Age \|\| Itm)** | 1 | 1 | 29126 | 29301 |
| **RT ~ 1 + Age * Lan * Siz + (0 + Siz \| ID) + (1 + Age \|\| Itm)** | 1 | 1 | 29126 | 29301 |
| **RT ~ 1 + Age * Lan * Siz + (1 + Siz \| ID) + (0 + Age \|\| Itm)** | 1 | 1 | 29124 | 29293 |
| **RT ~ 1 + Age * Lan * Siz + (0 + Siz \| ID) + (0 + Age \|\| Itm)** | 1 | 1 | 29124 | 29293 |
| **RT ~ 0 + Age * Lan * Siz + (1 + Siz \| ID) + (0 + Age \|\| Itm)** | 1 | 1 | 29124 | 29293 |
| **RT ~ 0 + Age * Lan * Siz + (0 + Siz \| ID) + (1 + Age \|\| Itm)** | 1 | 1 | 29126 | 29301 |
| **RT ~ 0 + Age * Lan * Siz + (0 + Siz \| ID) + (0 + Age \|\| Itm)** | 1 | 1 | 29137 | 29306 |
| **RT ~ 1 + Age * Lan * Siz + (1 + Lan \| ID) + (1 + Age \|\| Itm)** | 1 | 1 | 28627 | 28802 |
| **RT ~ 0 + Age * Lan * Siz + (1 + Lan \| ID) + (1 + Age \|\| Itm)** | 1 | 1 | 28627 | 28802 |
| **RT ~ 1 + Age * Lan * Siz + (0 + Lan \| ID) + (1 + Age \|\| Itm)** | 1 | 1 | 28621 | 28796 |
| **RT ~ 1 + Age * Lan * Siz + (1 + Lan \| ID) + (0 + Age \|\| Itm)** | 1 | 1 | 28619 | 28788 |
| **RT ~ 1 + Age * Lan * Siz + (0 + Lan \| ID) + (0 + Age \|\| Itm)** | 1 | 1 | 28619 | 28788 |
| **RT ~ 0 + Age * Lan * Siz + (1 + Lan \| ID) + (0 + Age \|\| Itm)** | 1 | 1 | 28619 | 28788 |
| **RT ~ 0 + Age * Lan * Siz + (0 + Lan \| ID) + (1 + Age \|\| Itm)** | 1 | 1 | 28621 | 28796 |
| **RT ~ 0 + Age * Lan * Siz + (0 + Lan \| ID) + (0 + Age \|\| Itm)** | 1 | 1 | 28619 | 28788 |
| **RT ~ 1 + Age * Lan * Siz + (1 + Lan \| ID) + (1 + Lan \|\| Itm)** | 1 | 0 | 28596 | 28731 |
| **RT ~ 0 + Age * Lan * Siz + (1 + Lan \| ID) + (1 + Lan \|\| Itm)** | 1 | 0 | 28596 | 28731 |
| **RT ~ 1 + Age * Lan * Siz + (0 + Lan \| ID) + (1 + Lan \|\| Itm)** | 1 | 0 | 28596 | 28731 |
| **RT ~ 1 + Age * Lan * Siz + (1 + Lan \| ID) + (0 + Lan \|\| Itm)** | 0 | 0 | 28594 | 28724 |
| **RT ~ 1 + Age * Lan * Siz + (0 + Lan \| ID) + (0 + Lan \|\| Itm)** | 0 | 0 | 28594 | 28724 |
| **RT ~ 0 + Age * Lan * Siz + (1 + Lan \| ID) + (0 + Lan \|\| Itm)** | 0 | 0 | 28594 | 28724 |
| **RT ~ 0 + Age * Lan * Siz + (0 + Lan \| ID) + (1 + Lan \|\| Itm)** | 1 | 0 | 28596 | 28731 |
| **RT ~ 0 + Age * Lan * Siz + (0 + Lan \| ID) + (0 + Lan \|\| Itm)** | 0 | 0 | 28594 | 28724 |
| Notes: Lan = Language (German vs French), Size = Number Size (30’s, 40’s, 50’s vs 70’s 80’s, 90’s), Age = 5^th^, 8^th^, 11^th^ and adults. ID = Subject, Itm = Item. Further degradation of the model were tested, but not shown here. The main models with all parameter, intercepts and random correlations (i.e. the “.0” models in the excel file), were tested using all_fit = TRUE and augmenting the iterations to 100000, leading to singularity and non convergence.  ^1^ Maximum Model (MM)  ^2^ Model however leads to singular fit with the auditory-visual matching task (M2)  ^3^ ( M3 )  ^4^ Model from the previous manuscript (M1) | | | | |

| Table 2: Comparison of the significance across the different analysis, sub-analysis and task datasets. | | | | | | | | | | | | | | |
| --- | --- | --- | --- | --- | --- | --- | --- | --- | --- | --- | --- | --- | --- | --- |
| ANOVA: | | | | | | | | | | | | | | |
|  |  | Lan * Size |  | Age * Lan * Size |  | 5th | 8th | 11th | Adult |  | 5th | 8th | 11th | Adult |
|  |  |  |  |  |  | Transparency of power | | | |  | LM2 cost | | | |
| Number reading | RT | <0.001 |  | .03 |  | <.001 | <.001 | <.001 | <.001 |  | <.001 | <.05 | 0.1 | 0.33 |
|  | zsRT | <.0001 |  | n.s. |  | **<.001** |  |  |  |  | **<.001** |  |  |  |
|  | 4Syll | <0.001 |  | n.s. |  | <.002 |  |  |  |  | <.002 |  |  |  |
| Visual-verbal matching | RT | <0.001 |  | <0.001 |  | <.001 | <.001 | <.01 | <.01 |  | <.001 | n.s. | n.s. | n.s. |
|  | zsRT | <0.001 |  | n.s. |  | **<.001** |  |  |  |  | **<.001** |  |  |  |
|  | 4Syll | <0.001 |  | <0.001 |  | <.001 | <.01 | .09 | .24 |  | <.001 | n.s. | n.s. | n.s. |
| Notes: Lan = Language (German vs French), Size = Number Size (30’s, 40’s, 50’s vs 70’s 80’s, 90’s), Age = 5^th^, 8^th^, 11^th^ and adults. Transparency of power = hypothesis on the cost for 70’s 80’s, 90’s in French. LM2 cost = hypothesis for a cost in French. RT = reaction time. zRT = per-age group individual z-score transformation. 4Syll = data restricted to 4 syllable long number words. | | | | | | | | | | | | | | |
|  |  |  |  |  |  |  |  |  |  |  |  |  |  |  |
| M1: (RT ~ 1 + Age * Lan * Siz + (1 + Lan \| ID) + (1 + Lan \| Itm)) | | | | | | | | | | | | | | |
|  |  | Lan * Size |  | Age * Lan * Size |  | 5th | 8th | 11th | Adult |  | 5th | 8th | 11th | Adult |
|  |  |  |  |  |  | Transparency of power | | | |  | LM2 cost | | | |
| Number reading | RT | <0.001 |  | <.003 |  | <.001 | <.001 | <.001 | <.001 |  | <.001 | .05 | .10 | .32 |
|  | zRT | <.0001 |  | n.s. |  | **<.001** |  |  |  |  | **<.001** |  |  |  |
|  | 4Syll | <0.001 |  | n.s. |  | <.002 |  |  |  |  | <.002 |  |  |  |
| Visual-verbal matching | RT | <0.001 |  | <0.001 |  | <.001 | <.001 | <.01 | <.01 |  | <.001 | n.s. | n.s. | n.s. |
|  | zRT | <0.001 |  | n.s. |  | **<.001** |  |  |  |  | **<.001** |  |  |  |
|  | 4Syll | <0.001 |  | <0.001 |  | <.001 | <.01 | .07 | .15 |  | <.001 | n.s. | n.s. | n.s. |
| Notes: items with post-error slipping are removed.  Lan = Language (German vs French), Size = Number Size (30’s, 40’s, 50’s vs 70’s 80’s, 90’s), Age = 5^th^, 8^th^, 11^th^ and adults. ID = Subject, Itm = Item. Transparency of power = hypothesis on the cost for 70’s 80’s, 90’s in French. LM2 cost = hypothesis for a cost in French. RT = reaction time. zRT = per-age group individual z-score transformation. 4Syll = data restricted to 4 syllable long number words. | | | | | | | | | | | | | | |
| M2: (RT ~ 1 + Age * Lan * Siz + (1 + Lan + Siz \| ID) + (1 + Lan \| Itm)) | | | | | | | | | | | | | | |
|  |  | Lan * Size |  | Age * Lan * Size |  | 5th | 8th | 11th | Adult |  | 5th | 8th | 11th | Adult |
|  |  |  |  |  |  | Transparency | | | |  | LM2 cost | | | |
| Number reading | RT | <.001 |  | 0.006 |  | <.001 | <.001 | <.001 | <.001 |  | <.001 | <.05 | .10 | n.s. |
|  | zRT | <.001 |  | .33 |  | <.001 |  |  |  |  | <.001 |  |  |  |
|  | 4Syll | <.001 |  | .36 |  | <.001 |  |  |  |  | <.001 |  |  |  |
| Visual-verbal matching | **Singular fit error** | | | | | | | | | | | | | |
| Notes: items following an error were removed.  Lan = Language (German vs French), Size = Number Size (30’s, 40’s, 50’s vs 70’s 80’s, 90’s), Age = 5^th^, 8^th^, 11^th^ and adults. ID = Subject, Itm = Item. Transparency of power = hypothesis on the cost for 70’s 80’s, 90’s in French. LM2 cost = hypothesis for a cost in French. RT = reaction time. zRT = per-age group individual z-score transformation. 4Syll = data restricted to 4 syllable long number words. | | | | | | | | | | | | | | |

1. (M1) RT ~ 1 + Age * Language * Number Size + (1 + Language | Subject) + (1 + Language | Item)
